# Engineered upconversion nanoparticles for breast cancer theranostics

**DOI:** 10.7150/thno.116153

**Published:** 2025-07-25

**Authors:** Shijing Wang, Lei Zhang, Minghao Wang, Xiumei Yin, Xinyao Dong, Xingyu Wu, Weijie Li, Wen Xu, Xiaoyun Mao

**Affiliations:** 1 Department of Breast Surgery, The First Affiliated Hospital of China Medical University, Shenyang, Liaoning Province, 110000, China.; 2 Key Laboratory of New Energy and Rare Earth Resource Utilization of State Ethnic Affairs Commission, School of Physics and Materials Engineering, Dalian Minzu University, Dalian, Liaoning Province, 116600, China.; 3 Department of Neurosurgery, The First Affiliated Hospital of China Medical University, Shenyang, Liaoning Province, 110000, China.; 4 Dalian Maritime University, Dalian, Liaoning Province, 116600, China.

**Keywords:** upconversion nanoparticles, breast cancer, theranostics, molecular imaging, biomarker detection, phototherapy, delivery, immunotherapy

## Abstract

Breast cancer (BC) remains the most prevalent cancer among women and a leading cause of cancer-related mortality worldwide, posing a significant threat to public health. Rare earth (RE)-doped upconversion nanoparticles (UCNPs) have emerged as a promising nanoplatform for BC management, owing to their exceptional photophysical properties and design flexibility. Unlike conventional fluorescent probes, engineered UCNPs absorb near-infrared (NIR) light, enabling deep tissue penetration while mitigating tissue damage and spontaneous fluorescence interference. Furthermore, through core-shell structure engineering and functionalization, multiple diagnostic and therapeutic modules can be integrated within a single NP, enabling theranostic applications for BC. This review comprehensively summarizes recent advances in engineered UCNPs for BC theranostics. It begins by introducing the luminescence mechanisms, controllable synthesis methods, and surface modification strategies of UCNPs. Next, it explores the fundamental biological effects of UCNPs, including biodistribution, metabolic pathways, and biotoxicity. Subsequently, we systematically review applications of engineered UCNPs in BC molecular imaging, biomarker detection, phototherapy, smart drug/gene delivery, and immunotherapy. Finally, current challenges and clinical translation prospects of UCNPs are discussed.

## 1. Introduction

**Figure 1:**
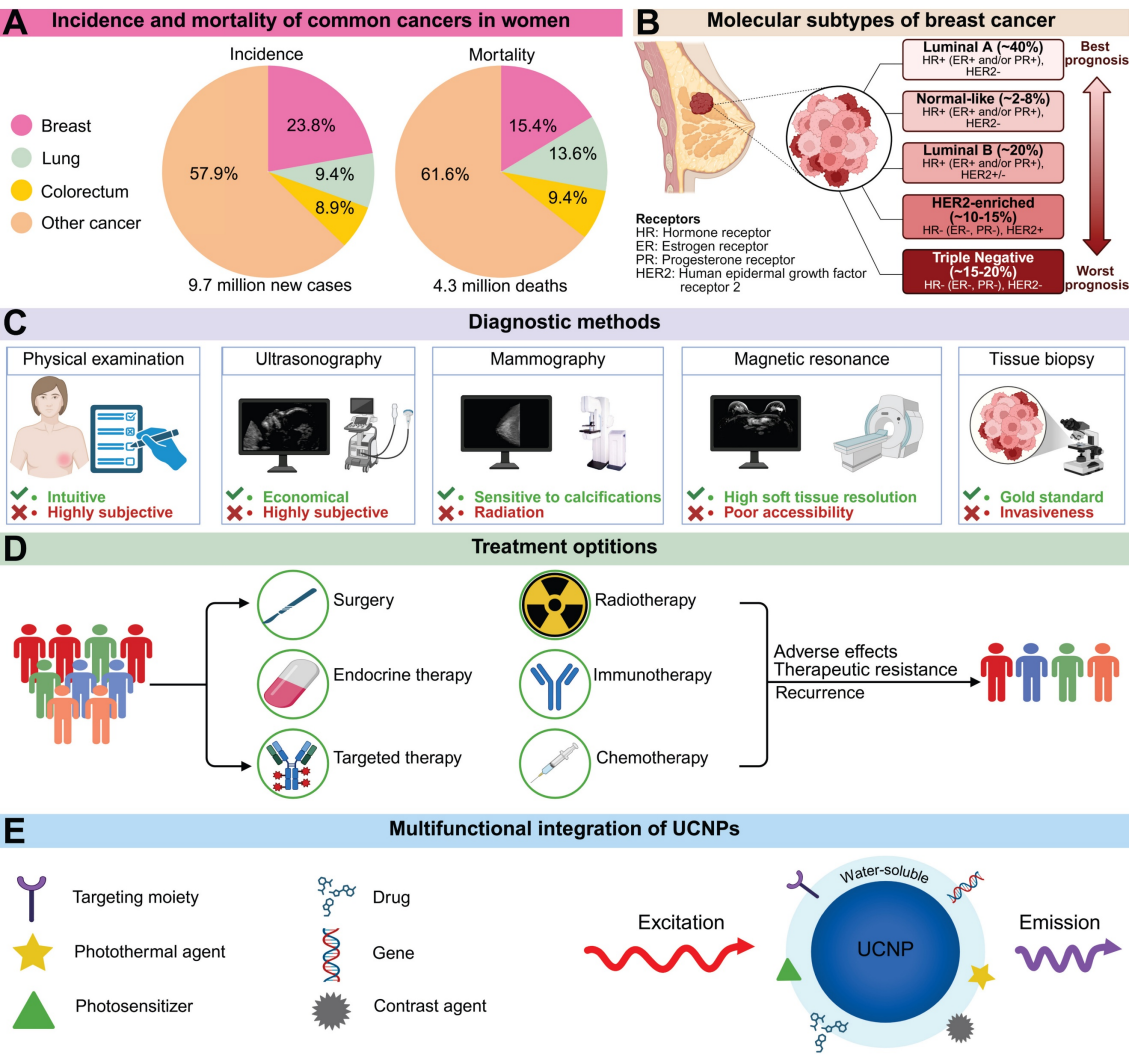
(A) Pie charts show the distribution of cases and deaths among the three top cancers affecting females in 2022. The size of each segment precisely reflects the proportion of the total number of cases or deaths. (B) Molecular subtypes of BC. Clinical status of strategies for BC diagnosis (C) and treatment (D). (E) Different types of UCNP-based designs for strategies in the diagnosis and treatment of cancer. Created with BioRender.com.

BC comprises a diverse spectrum of malignancies originating in the mammary glands and remains the most prevalent cancer among women. According to global cancer burden data, over 2.3 million new BC cases were diagnosed worldwide in 2022, causing approximately 670,000 deaths (Figure 1A) 1. Projections indicate that by 2050, global new BC cases will reach 3.2 million, with 1.1 million deaths, representing increases of 38% and 68%, respectively, from 2022 levels 2. The estimated lifetime risk of developing BC is 8-12%, while modifiable risk factors remain limited 3,4. Characterized by high heterogeneity, BC is classified into luminal A, normal-like, luminal B, HER2-enriched, and triple-negative breast cancer (TNBC) subtypes based on hormone receptor expression (estrogen receptor [ER], progesterone receptor [PR]), the proliferation marker Ki-67, and human epidermal growth factor receptor 2 (HER2) status. Each subtype exhibits distinct epidemiological characteristics, treatment responses, and prognostic outcomes (Figure 1B).

Current BC diagnosis relies on physical examination, imaging, and histopathology (Figure 1C). Physical examination includes thorough evaluation of the breast, lymph nodes, and potential distant metastases. However, early-stage BC patients often lack specific symptoms or signs, complicating diagnosis. Imaging modalities such as mammography and ultrasonography play crucial roles in the diagnostic workflow. Mammography clearly visualizes breast masses and calcifications; owing to its affordability and accessibility, it is considered the optimal screening modality when combined with ultrasonography 5. Nevertheless, mammography involves ionizing radiation exposure, while ultrasonography exhibits high operator dependence. Emerging technologies like AI-assisted analysis and high-throughput molecular staining demonstrate transformative potential, though technical standardization and clinical validation remain significant barriers to widespread adoption 6. Furthermore, BC treatment has advanced dramatically over the past century, evolving from purely surgical interventions to comprehensive, coordinated strategies integrating local and systemic therapies (Figure 1D). However, these approaches face limitations including surgical morbidity, non-selective chemotherapy toxicity, radiotherapy-related side effects, and restricted applicability of immunotherapy, endocrine therapy, and targeted agents. Therefore, integrating multidisciplinary approaches to develop flexible, tailored management strategies is essential for BC care.

In recent years, nanomaterials have demonstrated significant potential in biomedicine due to their unique size effects, high specific surface area, and tunable physicochemical properties 7. Among these, RE-doped UCNPs exhibit exceptional optical characteristics. Unlike conventional luminescent materials that emit longer-wavelength light under shorter-wavelength excitation, UCNPs convert NIR excitation into shorter-wavelength emissions through photon upconversion 8. This anti-Stokes process, termed upconversion luminescence (UCL), overcomes deep-tissue signal attenuation inherent to short-wavelength light while minimizing interference from endogenous fluorophores and reducing phototoxicity risks 9. Specifically, light penetration in tissues is fundamentally limited by absorption and scattering, with longer wavelengths enabling greater depth penetration 10,11. For instance, visible light exhibits shallow penetration in biological tissues, typically reaching only about 1 mm 12. The optical penetration depth increases from 0.19 mm for 632.8 nm red light to 0.51 mm for 835 nm light in blood, and from 2.59 mm to 3.54 mm in mammary tissue 13. Moreover, endogenous fluorophores predominantly absorb UV/visible light, reducing the signal-to-noise ratio, while short-wavelength excitation can trigger photochemical reactions that damage biomolecules 14. Compared with other fluorescent probes, UCNPs show superiority in multiple dimensions (Table 1). Compared with fluorescent proteins, UCNPs do not require complex genetic encoding and have stronger resistance to photobleaching. In contrast to NIR organic dyes, UCNPs have distinct emission spectra and large anti-Stokes shifts, effectively avoiding overlap between excitation and detection signals. In complex physiological environments, the chemical inertness of UCNPs enables them to maintain long-term stability, whereas organic-inorganic hybrid fluorophores are vulnerable to the effects of pH and enzymes. Notably, the abundant ligand-binding sites on the surface of UCNPs provide favorable conditions for integrating multifunctional modules such as targeting molecules and therapeutic payloads (Figure 1E) 15. The resulting nanocomposites have shown tremendous promise in fields including tumor therapy, small-animal *in vivo* imaging, and molecular detection.

As a superficial organ, the breast exhibits optical properties characterized by moderate light scattering, highly forward-directed scattering, and low NIR absorption, making it ideally suited for applications of UCNPs 16-19. Meanwhile, as programmable nanoplatforms, UCNPs enable precise intervention in different molecularly stratified BC through modular design, which is highly congruent with clinical requirements 20. Recent advancements in engineered UCNPs have shown remarkable progress across multiple domains of BC management, including subtype-specific biomarker detection, molecular phenotype-adapted multimodal imaging, personalized phototherapy, smart drug/gene delivery, and immunotherapy. In this context, a systematic review of literature on UCNP-based BC theranostics is particularly necessary. However, most existing reviews focus on oncology at large, which have the drawbacks of broad research scopes and vague disease characteristics. Specifically, most reviews treat all malignant tumors as a single pathological entity, ignoring tumor heterogeneity and especially lacking systematic analysis of BC. While such integrative research helps explore common principles, it hinders the development and clinical translation of nanomedicine platforms tailored for BC. In this review, we first introduce UCNPs, covering their optical properties, advanced synthesis methods, and diverse functionalization strategies. We then discuss the *in vivo* biodistribution, clearance pathways, and potential biosafety issues of UCNPs. Building on this, we provide a comprehensive review of the applications of engineered UCNPs in BC diagnosis and therapy. Finally, we critically evaluate current limitations and propose strategic directions for future research, aiming to bridge the gap between basic science and clinical practice.

## 2. Introduction of UCNPs

**Figure 2:**
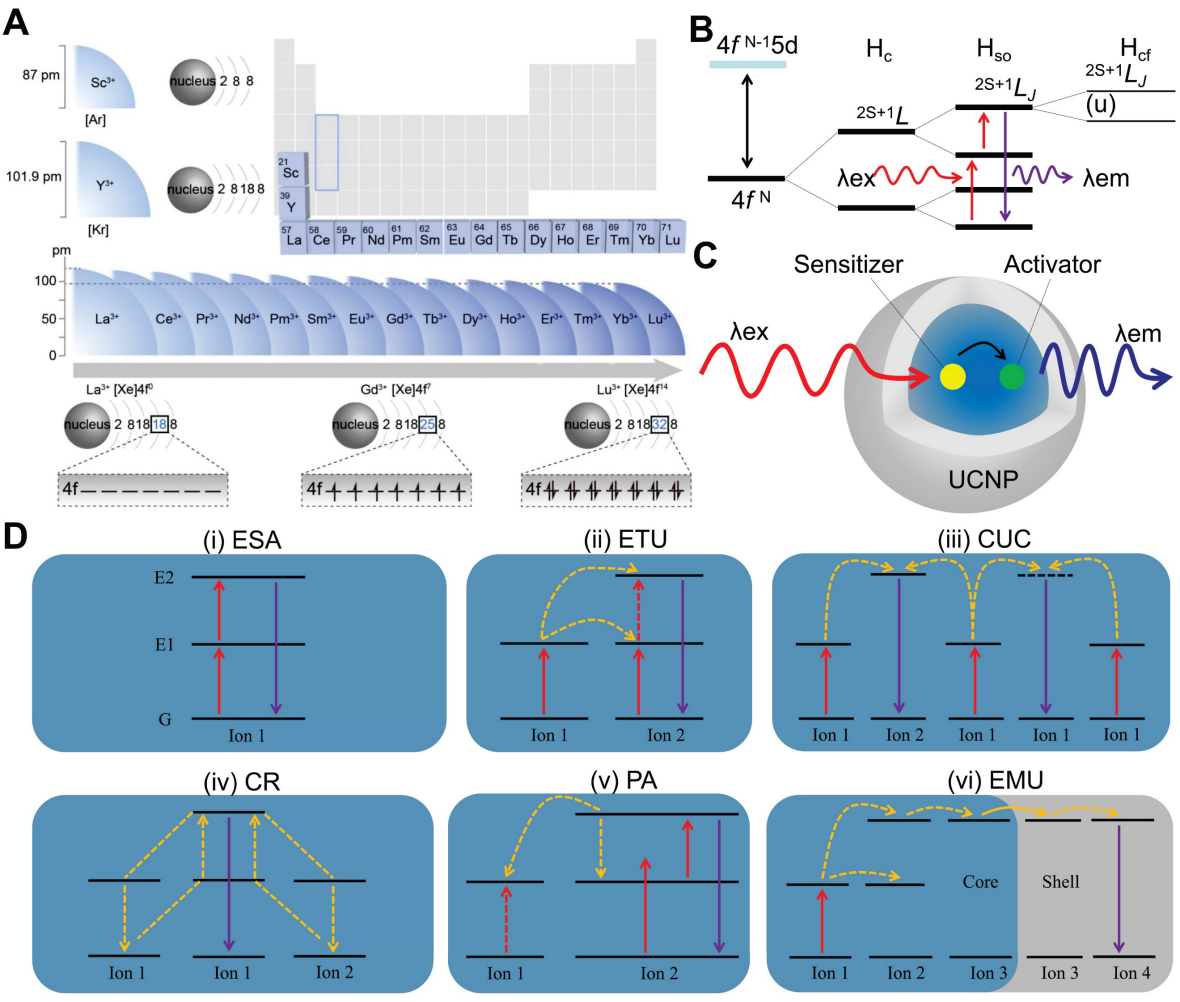
(A) Ionic radius and valence configuration of RE. From La^3+^ to Lu^3+^, the number of electrons on the 4f orbitals increases with increasing atomic number. The electron configurations of La^3+^, Gd^3+^, and Lu^3+^ show empty, half-filled, and completely filled 4f orbitals, respectively. Adapted with permission from 21, copyright 2022, American Chemical Society. (B) Simplified energy level diagrams of lanthanide ions for a basic upconversion process. The 4*f* ^N^ configuration of lanthanide ions splits into multiple energy sublevels due to the effects of the coulombic (H_c_), spin-orbit (H_so_) and crystal-field (H_cf_) interactions. The energy levels are denoted as *^2S+1^L*_J_, where *S*, *L*, and *J* stand for total spin, orbital, and total angular momentum quantum numbers. (C) Schematic illustrations for UCNPs and the tipical energy transfer process. (D) Schematic diagrams of six upconversion processes. Created with BioRender.com. λex: excitation spectrum; λem: emission spectrum; ESA: excited state absorption; ETU: energy transfer upconversion; CUC: cooperative upconversion; CR: cross-relaxation; PA: photon avalanche; EMU: energy migration upconversion.

RE elements, comprising Sc, Y and the fifteen lanthanides, are renowned as the "vitamin" of modern industry due to their superior physicochemical properties. Doping RE ions into inorganic materials not only modulates the crystal phase, morphology, size, and electronic structure of materials, but also endows the doped materials with rich optical, electrical, magnetic, and catalytic properties. Notably, RE elements exhibit a systematic ionic radius contraction and share similar electronic configurations (Figure 2A) 21. Except for Sc^3+^, Y^3+^, La^3+^, and Lu^3+^, the 4f electrons of other RE ions follow quantum mechanical principles, populating seven degenerate orbitals and generating intricate energy-level systems. These long-lived metastable states undergo fine splittings under spin-orbit coupling and crystal field effects, constituting the energy-level foundation necessary for UCL (Figure 2B) 22. The concept of UCL was first proposed by Nobel laureate Nicolaas Bloembergen in 1959, who envisioned using the multi-level energy structure of RE ions for NIR light detection 23. In 1966, Auzel, Ovsyankin, and Feofilov independently observed and interpreted this phenomenon in RE-doped bulk materials, marking the official commencement of research on upconversion luminescent materials 24,25.

In the following decades, upconversion luminescent materials were confined to bulk materials such as glasses and ceramics, suffering from issues including low energy conversion efficiency, high excitation power thresholds, and the difficulty in controlling complex multi-ion cooperative mechanisms 26. The emergence of nanoscience and various nanomanufacturing technologies in the 1990s inspired researchers to miniaturize upconversion luminescent materials to the nanoscale. One of the earliest attempts was reported in 1999 by Zijlmans *et al.*, who prepared 0.2-0.4 μm Y_2_O_2_S:Yb,Er and Y_2_O_2_S:Yb,Tm microparticles via superheating as upconversion luminescent biolabels for immunoassays 27. Nevertheless, this approach was constrained to producing particles with minimum sizes of several hundred nanometers, which hindered their broader utilization. It was not until the early 21st century that novel synthetic methodologies, such as co-precipitation and hydrothermal/solvothermal synthesis, emerged for preparing high-quality crystals with sizes below 100 nm, thereby driving significant advancements in this field 28. These techniques have enabled the reproducible fabrication of monodisperse UCNPs with precisely tunable size, morphology, and crystallinity. Leveraging their unique nanoscale properties and structural tunability, these UCNPs offer not only tailored optical characteristics but also serve as versatile platforms for multifunctional design. Today, UCNPs have become a highly interdisciplinary research field at the intersection of materials science and biomedicine 29,30.

### 2.1. Mechanism

Typically, UCNPs consist of an inorganic matrix, activator, and sensitizer 31. The matrix (e.g., phosphate, oxide, or fluoride) provides an optically inert framework with low phonon energy, thereby modulating crystal field splitting of RE ions and suppressing non-radiative decay 32. Activator ions serve as luminescent centers, populating high-energy excited states through energy harvesting and subsequently emitting anti-Stokes radiation. Sensitizer ions, characterized by broad absorption cross-sections, efficiently harvest low-energy photons and transfer energy to activators via dipole-dipole interactions or exchange mechanisms. As illustrated in Figure 2C, the upconversion process initiates with photon absorption by sensitizers, followed by non-radiative energy migration to activators, which are excited to higher energy states for emitting high-energy photons. Specific mechanisms include excited state absorption, energy transfer upconversion, cooperative upconversion, cross-relaxation, photon avalanche, and energy migration upconversion.

#### 2.1.1. Excited state absorption

Excited state absorption, proposed by Bloembergen in 1959, involves a single RE ion absorbing photons sequentially, transitioning stepwise from the ground state to higher energy levels via intermediate excited states, and then decaying to the ground state with emission of a higher-energy photon (Figure 2D [i]) 23. This necessitates RE ions to possess energy levels arranged in a ladder-like fashion with near-even spacing, thus restricting this process to a limited number of ions (e.g., Er^3+^, Ho^3+^, Tm^3+^, and Nd^3+^). Additionally, efficiency is typically constrained by factors including short excited-state lifetimes, energy level configurations distinct from ground states, and complex multiphoton absorption mechanisms, all of which reduce absorption probabilities 33. Recently, Dong *et al.* achieved a breakthrough by employing layered ternary RE sulfides as host matrices. By leveraging the high absorption cross-section of Er^3+^ during the ^4^I_15/2_→^4^I_13/2_ transition and optimizing the prolonged lifetime in the ^4^I_9/2_ state, they achieved enhanced excited state absorption upconversion in NaYF_4_:Er, yielding a visible upconversion efficiency of 2.6% 34.

#### 2.1.2. Energy transfer upconversion

Energy transfer upconversion is the most common upconversion mechanism in UCNPs, differing from excited state absorption by involving energy transfer between multiple ions (Figure 2D [ii]). This mechanism necessitates a close spatial proximity between sensitizers and activators to enable efficient energy transfer via dipole-dipole interactions, along with precise energy level alignment to minimize non-radiative losses. Additionally, sensitizers should exhibit a larger absorption cross-section than activators to maximize energy harvesting efficiency under low intensity excitation 35. Yb^3+^ is frequently employed as a sensitizer owing to its well-matched 980 nm absorption cross-section with commercial diode lasers. NaYF_4_:Yb,Er has served as a highly efficient 980 nm-to-visible upconversion phosphor since the 1970s 36.

#### 2.1.3. Cooperative upconversion

Cooperative upconversion involves interactions among at least three ions, categorized into cooperative sensitization and cooperative luminescence (Figure 2D [iii]). In cooperative sensitization, two sensitizer ions jointly transfer energy to a single activator ion, enabling its transition to a higher excited state for upconversion. Cooperative luminescence differs by eliminating intermediate states, instead coupling emission directly to paired ion interactions in which identical RE ions (e.g., Yb^3+^-Yb^3+^ pairs) serve as both energy donors and acceptors 37. The involvement of virtual pair-level transitions in these processes imposes inherent quantum mechanical constraints, leading to significantly lower efficiency than that of excited state absorption and energy transfer upconversion.

#### 2.1.4. Cross-relaxation

Cross-relaxation is an energy transfer process occurring between excited ions, either identical or distinct (Figure 2D [iv]). It is generally regarded as detrimental to upconversion efficiency and constitutes the primary cause of concentration quenching 38. This process proceeds via two pathways dictated by energy level configurations. Energy migration entails resonant energy transfer between adjacent ions with equivalent energy levels, preserving the system's energy equilibrium. In contrast, cross-energy-level cross-relaxation induces energy redistribution between mismatched electronic states, leading to spectral alterations or non-radiative losses that manifest as self-quenching effects, particularly in heavily doped systems 39. Nevertheless, cross-relaxation can be strategically harnessed to modulate emission profiles in specific contexts. For example, Chen *et al.* realized single-band red UCL of Ho^3+^ by incorporating Ce^3+^ into Yb^3+^-Ho^3+^-codoped UCNPs, exploiting cross-relaxation between Ce^3+^ and Ho^3+^ 40.

#### 2.1.5. Photon avalanche

Photon avalanche, first conceptualized by Chivian *et al.* in 1979, is a process involving a cycle of excited state absorption and cross-relaxation (Figure 2D [v]) 41. Initially, ground-state electrons are promoted to higher energy levels through excited state absorption. Subsequently, these excited electrons undergo cross-relaxation with neighboring sensitizer ions, causing activator ions to transition to an intermediate energy level while sensitizers move to an excited state. This interaction establishes a positive feedback loop via energy back-transfer, resulting in exponential electron population at intermediate levels and intense emission even at low activator concentrations. However, this process demands high excitation power densities, confining its applicability to systems doped with Pr^3+^, Tm^3+^, or Nd^3+^ ions 42,43. Notably, photon avalanche exhibits exceptionally high optical nonlinearity, wherein luminescence intensity increases exponentially once the excitation intensity surpasses a critical threshold. This property facilitates applications in super-resolution imaging, where the ultrahigh nonlinearity circumvents the traditional diffraction limit, enabling single-molecule or single-ion detection.

#### 2.1.6. Energy migration upconversion

Energy migration upconversion refers to a luminescence mechanism where RE ions are classified into sensitizers, energy-storage mediators, migration ions, and activators based on their roles in the energy transfer cascade. Core-shell engineering spatially segregates RE ions into distinct layers, thereby minimizing non-radiative losses and enabling directional energy transfer. As illustrated in Figure 2D (vi), sensitizer ions absorb pump photons and funnel energy to activator ions via energy-storage and migration ions. Activator ions subsequently emit photons as their excited electrons relax from high-energy states to the ground state. This mechanism utilizes energy-storage and migration ions to enable efficient upconversion in ions lacking suitable intermediate energy levels or exhibiting ultrashort-lived metastable states, such as Ce^3+^, Gd^3+^, Tb^3+^, Dy^3+^, Eu^3+^, Sm^3+^, and Sm^2+^ 44. It enables high upconversion efficiency under low-power excitation, thereby promoting the applications of UCNPs.

### 2.2. Optical tuning

In biomedical applications, achieving high-contrast imaging, multiplexed biosensing, and spatiotemporally controlled therapy requires precisely tunable luminescence intensity and color. However, UCNPs face challenges due to significant non-radiative surface quenching caused by their high surface-to-volume ratio, as well as the limitations of RE elements, such as narrow excitation spectra and fixed emission wavelengths. Initial studies focused on regulating size and dopant concentration to modulate emission intensity, but these approaches couldn't generate new emission peaks or broaden the excitation bandwidth 45-47. Further advancements have been made by utilizing energy transfer between multiple dopants to enhance tuning, but uncontrolled cross-relaxation processes between different RE ions cause substantial quenching effects 48. Emerging research now prioritizes structural engineering approaches, particularly core-shell structures, which demonstrate multidimensional regulatory capabilities surpassing conventional single-parameter optimization methods. Unlike morphology regulation or component optimization, these hierarchical nanostructures enable synergistic control over luminescence efficiency, spectral tunability, and functional integration through precise interfacial engineering.

#### 2.2.1. Enhancement of emission

**Figure 3:**
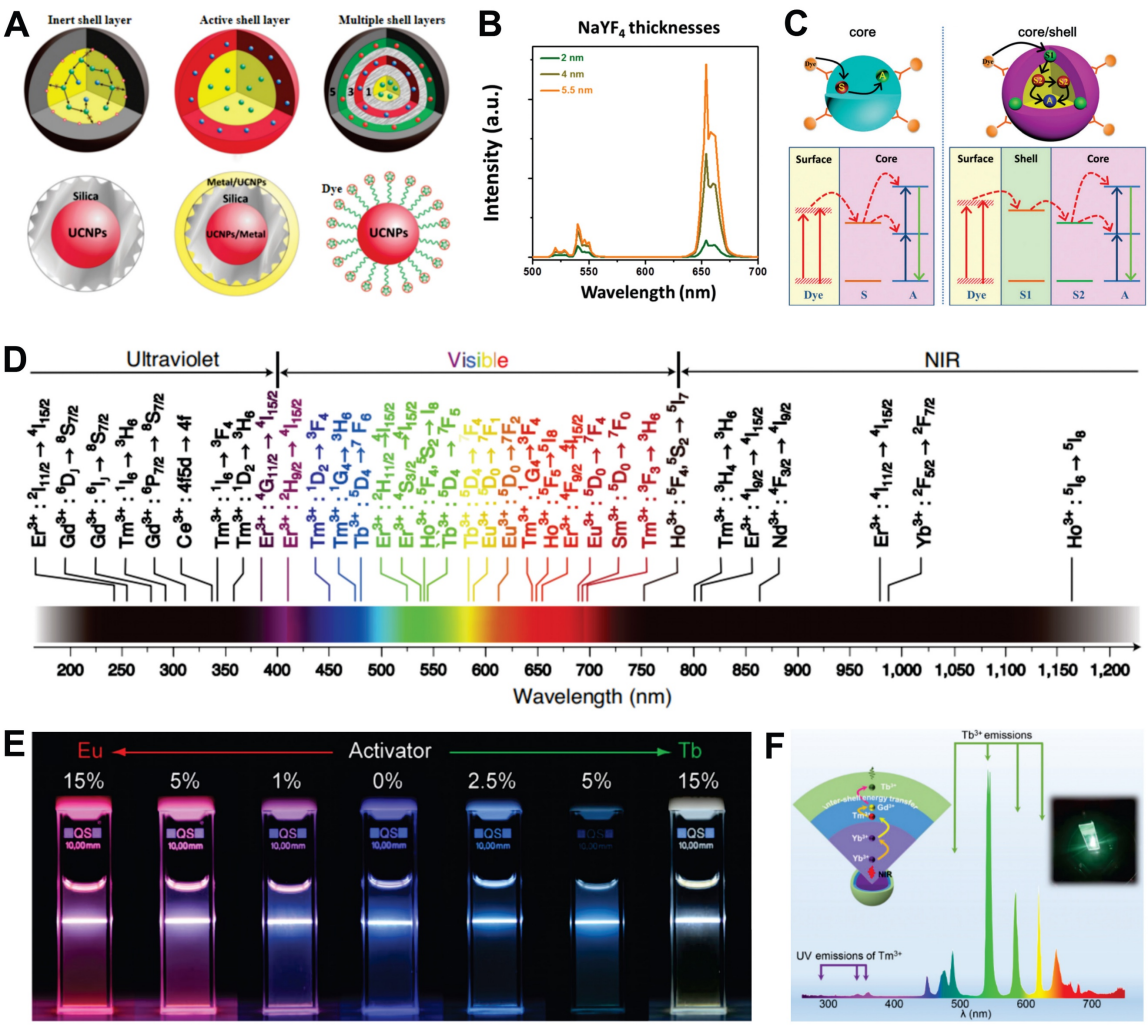
(A) Schematic diagram of the core-shell structures. Adapted with permission from 49, copyright 2015, Royal Society of Chemistry. (B) The shell-thicknesses dependent upconversion emission spectra of NaErF_4_@NaYF_4_ NPs. Reproduced with permission 52, copyright 2021, under a Creative Commons CC BY license. (C) Schematic illustrations of dye sensitized upconversion in the core (S, sensitizer; A, activator) (left) and the core-shell structure (right). Adapted with permission from 55, copyright 2021, American Chemical Society. (D) A summary of the upconversion transitions of RE ion, covering a broad range of wavelengths from the UV to the NIR. Created with BioRender.com. (E) UCL of core-shell NPs with different Eu^3+^/Tb^3+^ doping concentrations under 980 nm excitation. Adapted with permission from 57, copyright 2011, Springer Nature Limited. (F) Schematic illustration of the sandwich core-shell nanostructure. Adapted with permission from 59, copyright 2021, Chinese Chemical Society.

Core-shell structures can be categorized into epitaxial and nonepitaxial shell layers based on the growth process. Epitaxial shell layers, which include inert, active, and multiple shells, have a composition and structure similar to the matrix, providing a robust crystal field and preventing surface quenching. In contrast, nonepitaxial shell layers typically consist of materials like SiO_2_, metal, and organic dyes (Figure 3A) 49. The active core-inert shell structure involve a UCNP-containing core encapsulated by an inert matrix-material shell. This design isolates the core from the external environment and passivates surface defects, significantly enhancing UCL. Examples of such systems include NaYF_4_@NaYF_4_, SrF_2_@SrF_2_, and NaYF_4_@CaF_2_ 50,51. Within a specific range, increasing the shell thickness reduces ion cross-relaxation, leading to enhanced luminescence intensity. For instance, coating an inert NaYF_4_ shell on an active NaErF_4_ core resulted in stronger emission as the shell thickness increased from 2 nm to 5.5 nm (Figure 3B) 52. On the basis of the inert shell, an active shell can be fabricated by introducing sensitizer into the shell layer. The spatial separation between sensitizers in the core and shell mitigates concentration quenching, thereby enhancing the absorption efficiency of excitation light. For example, Fu *et al.* achieved a 1643-fold enhancement in visible light emission and a 33-fold enhancement in NIR emission by incorporating Yb^3+^ as an energy transfer mediator in the shell 53. Moreover, in comparison with single layer shell structures, multilayer core-shell nanostructures exhibit enhanced flexibility and multifunctionality in terms of their structural and surface properties.

Nonepitaxial shells enhance UCL by reducing concentration quenching, utilizing dye sensitization, and exploiting local surface plasmon resonance (SPR). For example, NaErF_4_@SiO_2_ core-shell structures exhibit significantly enhanced UCL compared to NaErF_4_ cores, despite minimal changes in morphology and structure 54. Coupling UCNPs with organic dyes improves light-harvesting efficiency due to the low extinction coefficients and narrow absorption bands of RE ions. Building on this approach, energy cascade upconversion via dye sensitization involves organic dyes absorbing excitation light, transferring energy to shell ions, and then to core ions, thereby enhancing UCL (Figure 3C) 55. Moreover, the resonance phenomenon enhances the local electric field around the metal shell, which to some extent enhances the absorption efficiency of the sensitizer and the radiative decay rate of the activator in UCNPs. However, UCL enhancement occurs only when an appropriate distance is maintained between UCNPs and the metal shell, necessitating an isolation layer with optimal thickness 56.

#### 2.2.2. Color modulation of emissions

The UCL emission spectra of UCNPs exhibit remarkably broad emission bands, spanning from the UV to NIR spectral region (Figure 3D). However, the diversity of RE types and multiple energy levels within ions pose challenges for precise color control. The spatial confinement effect of core-shell structures provides a systematic solution to address this challenge. A prime example is the NaGdF_4_:Yb,Tm@NaGdF_4_:Eu,Tb,Dy,Sm system, which precisely manipulates complex energy transfer pathways among RE dopants through controlled doping concentration adjustments (Figure 3E) 57. In a subsequent study, Qin *et al.* engineered a multi-layer core-shell structured UCNPs by incorporating Tm^3+^, Ho^3+^, and Er^3+^ ions into the NaYF_4_ matrix, enabling single-NP-level tricolor UCL across visible to NIR spectral regions 58. Recently, Chen *et al.* developed a sandwich-type core-shell nanostructure with Yb^3+^ in the core, Tm^3+^ in the intermediate shell, and Tb^3+^ in the outer shell, significantly reducing background fluorescence interference from Tm^3+^ (Figure 3F) 59. Furthermore, shell-structured orthogonal upconversion for emission color adjustment has opened new avenues for various applications.

## 3. Synthesis Methods of UCNPs

Significant advancements have been made in UCNP synthesis over the past two decades, enabling precise control over structural parameters such as crystallographic phase, size, and morphology. Diverse synthetic methods have been developed, including thermal decomposition, co-precipitation, hydrothermal/solvothermal synthesis, sol-gel processes, microwave-assisted synthesis, microemulsion techniques, and hybrid approaches. Among these, thermal decomposition, co-precipitation, and hydrothermal/solvothermal synthesis are the dominant strategies for fabricating high-quality nanocrystals by precisely regulating crystal growth kinetics. Furthermore, near-atomic-scale material engineering has enabled the design of core-shell architectures, enhancing functional capabilities through optimized interfacial energy transfer modulation.

### 3.1. Synthesis of core

#### 3.1.1. Thermal decomposition

**Figure 4:**
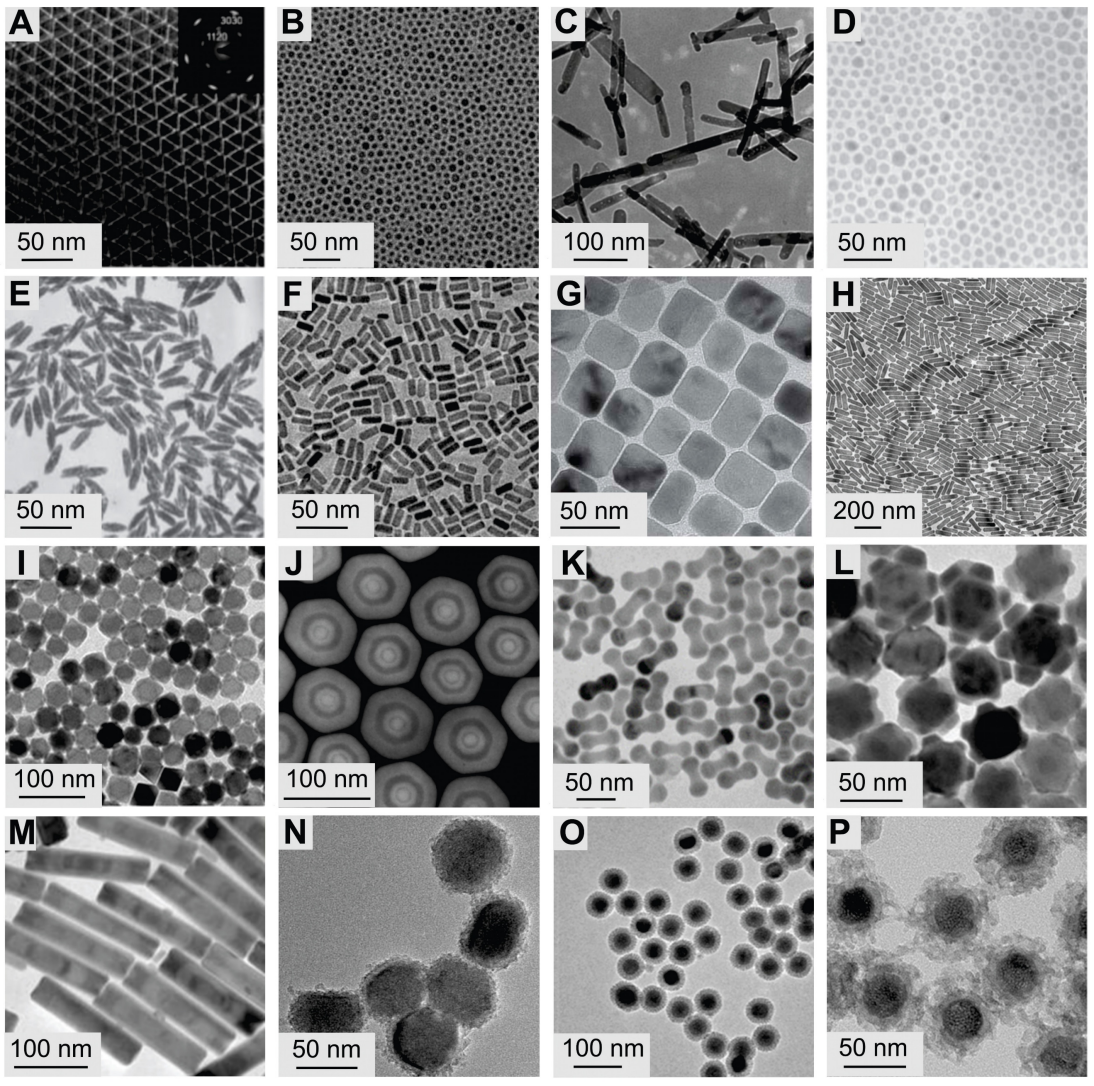
Typical transmission electron microscope images of UCNPs. (A) LaF_3_ (Adapted with permission from 61, copyright 2005, American Chemical Society), (B) NaYF_4_ (Adapted with permission from 62, copyright 2006, WILEY-VCH Verlag GmbH & Co. KGaA, Weinheim) and (C) Gd_2_O_3_ (Adapted with permission from 66, copyright 2012, Elsevier Ltd. All rights reserved) NPs synthesized by thermal decomposition. (D) NaYF_4_ (Adapted with permission from 73, copyright 2005, Springer Nature Limited), (E) YF_3_ (Adapted with permission from 73, copyright 2005, Springer Nature Limited), and (F) NaGdF_4_ (Adapted with permission from 72, copyright 2016, American Chemical Society) nanocrystals synthesized by hydrothermal/solvothermal strategy. (G) NaScF_4_ (Adapted with permission from 76, copyright 2013, Royal Society of Chemistry) and (H) KYb_2_F_7_ (Adapted with permission from 77, copyright 2013, Springer Nature Limited) nanocrystals synthesized by coprecipitation. (I) LiLuF_4_@LiLuF_4_ (Adapted with permission from^81^, copyright 2014, WILEY-VCH Verlag GmbH & Co. KGaA, Weinheim) NPs synthesized by seed-mediated heat-up. (J) GdF_4_@NaYF_4_@NaGdF_4_@NaYF_4_@NaGdF_4_ (Adapted with permission from 83, copyright 2016, WILEY-VCH Verlag GmbH & Co. KGaA, Weinheim) nanocrystals synthesized by successive layer-by-layer method. (K) NaGdF_4_@NaYF_4_ nano-dumbbells, (L) NaYF_4_@NaGdF_4_@NaNdF_4_ flower-shaped nanocrystals, (M) NaYF_4_@NaGdF_4_ bamboo-like nanorods (Adapted with permission from 65, copyright 2016, under a Creative Commons CC BY license). (N) NaYF_4_@TiO_2_ (Adapted with permission from 89, copyright 2020, Elsevier Ltd. All rights reserved), (O) NaYF_4_@NaYF_4_@SiO_2_ (Adapted with permission from 87, copyright 2020, The Society of Powder Technology Japan. Published by Elsevier B.V. and The Society of Powder Technology Japan. All rights reserved), and (P) NaYF_4_@mSiO_2_ (Adapted with permission from 86, copyright 2022, American Chemical Society) NPs synthesized by nonepitaxial growth.

In thermal decomposition, metal-organic precursors are introduced into solvents under anhydrous and oxygen-free conditions, with decomposition occurring at elevated temperatures 60. Reaction solvents are typically categorized into non-coordinating and coordinating types. Non-coordinating solvents provide a high-temperature environment for rapid NP nucleation and crystal transformation, while coordinating solvents adsorb onto the surfaces of particles to inhibit growth and agglomeration. In 2005, Yan *et al.* synthesized highly monodisperse LaF_3_ NPs via thermally decomposing trifluoroacetate at 280 °C (Figure 4A) 61. Subsequently, Chow *et al.* used oleylamine as both the reaction solvent and surface ligand to synthesize NaYF_4_ NPs, enabling the phase transition from cubic to hexagonal structure (Figure 4B) 62. Subsequent studies synthesized various fluoride crystals by adjusting the precursor concentration ratio, reaction temperature, reaction duration, and solvent type 63-65. This method has also been applied to the synthesis of lanthanide oxides, oxyfluorides, and oxychlorides (Figure 4C) 66-68. Thermally decomposed nanocrystals are high-quality, featuring pure crystal phases and uniform morphology. However, this method requires stringent synthesis conditions and incurs relatively high costs. Moreover, metal-organic precursors are prone to oxidation, potentially leading to the generation of toxic by-products.

#### 3.1.2. Hydrothermal/solvothermal synthesis

Hydrothermal/solvothermal synthesis is a method to produce high-quality UCNPs at relatively low temperatures using water or organic solvents. Precursors typically include nitrates, chlorides, and oxides of RE ions. For LnF_3_ nanocrystal synthesis, fluoride compounds such as HF, NH_4_F, or NH_4_HF_2_ are often used, while NaF or KF is preferred for synthesizing MLnF_4_ nanocrystals. Common organic solvents, such as oleic acid, polyethylenediamine, and ethylenediaminetetraacetic acid, are used 69. Based on this method, Li *et al.* utilized the "liquid-solid-solution" strategy to synthesize various high-quality fluoride matrices, such as NaYF_4_, NaGdF_4_, and YF_3_ (Figure 4D-F) 70-73. This method has also been successfully used to prepare lanthanide-doped oxide UCNPs with size-controllable and morphology-tunable features. Hydrothermal/solvothermal synthesis is an environmentally benign approach, but it is time-consuming and highly reliant on reaction vessels, thereby challenging real-time observation and monitoring of the reaction process.

#### 3.1.3. Coprecipitation

Coprecipitation is a process where multiple metal ions precipitate and crystallize simultaneously in the presence of a precipitating agent. Veggel *et al.* pioneered this method via coprecipitation of trivalent RE ions and F^-^ in an ethanol-water solution at 75 °C 74. However, the initial nanocrystals produced by this method had low luminescence intensity. To address this, researchers dissolved precursors at low temperatures and used heat treatment to transform amorphous seed crystals into well-crystallized nanocrystals. For example, Guo *et al.* synthesized NaYF_4_ NPs by mixing ethylene-diamine-tetraacetic acid with RE ions and rapidly adding the mixture to a NaF solution. Subsequent thermal treatment increased fluorescence intensity by 40 times compared to pre-annealing 75. Currently, via post-heat treatment and surfactant addition, a wide range of NPs, such as NaScF_4_ and KYb_2_F_7_, can be synthesized (Figure 4G-H) 76,77. This method is simple to operate, low-cost, and highly reproducible, but it requires calcination (post-annealing) of precipitates, which may induce nanocrystal aggregation and overgrowth.

### 3.2. Synthesis of core-shell

With advances in materials engineering, core-shell UCNPs exhibit greater tunability in morphology, size, composition, and surface properties. To date, various fabrication strategies have been employed to construct core-shell UCNPs. The seed-mediated heat-up method involves preparing core UCNPs first, which then serve as seeds for epitaxial shell growth. This method has successfully synthesized diverse core-shell nanostructures such as EuF_3_@GdF_3_, NaYbF_4_@CaF_2_, NaGdF_4_@NaGdF_4_, NaYF_4_@NaYF_4_, and LiLuF_4_@LiLuF_4_, though it may lead to non-uniform shell thickness (Figure 4I) 78-82. The successive layer-by-layer method is used for fabricating uniform multilayered structures with precise shell thickness control (Figure 4J) 83, as demonstrated by Li *et al.* who adjusted shell precursor concentrations to achieve shell thicknesses from 0.36 to 8 nm 84. Diverse morphologies can also be achieved through ion migration, as shown by Jin *et al.* who controlled the ratio of oleate anions to cations to form 3D morphologies such as dumbbells, flowers, and bamboo-like structures (Figure 4K-M) 65. Ostwald ripening, which involves the dissolution of small particles and growth of larger ones, also leads to core-shell formation. Cation exchange, pioneered by Veggel *et al.* in 2009, involves exchanging RE ions on the UCNPs' surface with cations in the reaction solution to form a distinct shell, though it is unsuitable for multilayer structures 85. Nonepitaxial growth refers to the deposition of shell materials (e.g., noble metals or SiO_2_) on UCNPs without crystallographic alignment with the core, resulting in an amorphous or polycrystalline shell structure (Figure 4N-P) 86-90. This structural characteristic not only preserves the intrinsic optical properties of UCNPs but also offers a versatile platform for subsequent surface modifications.

## 4. Surface Engineering

**Figure 5:**
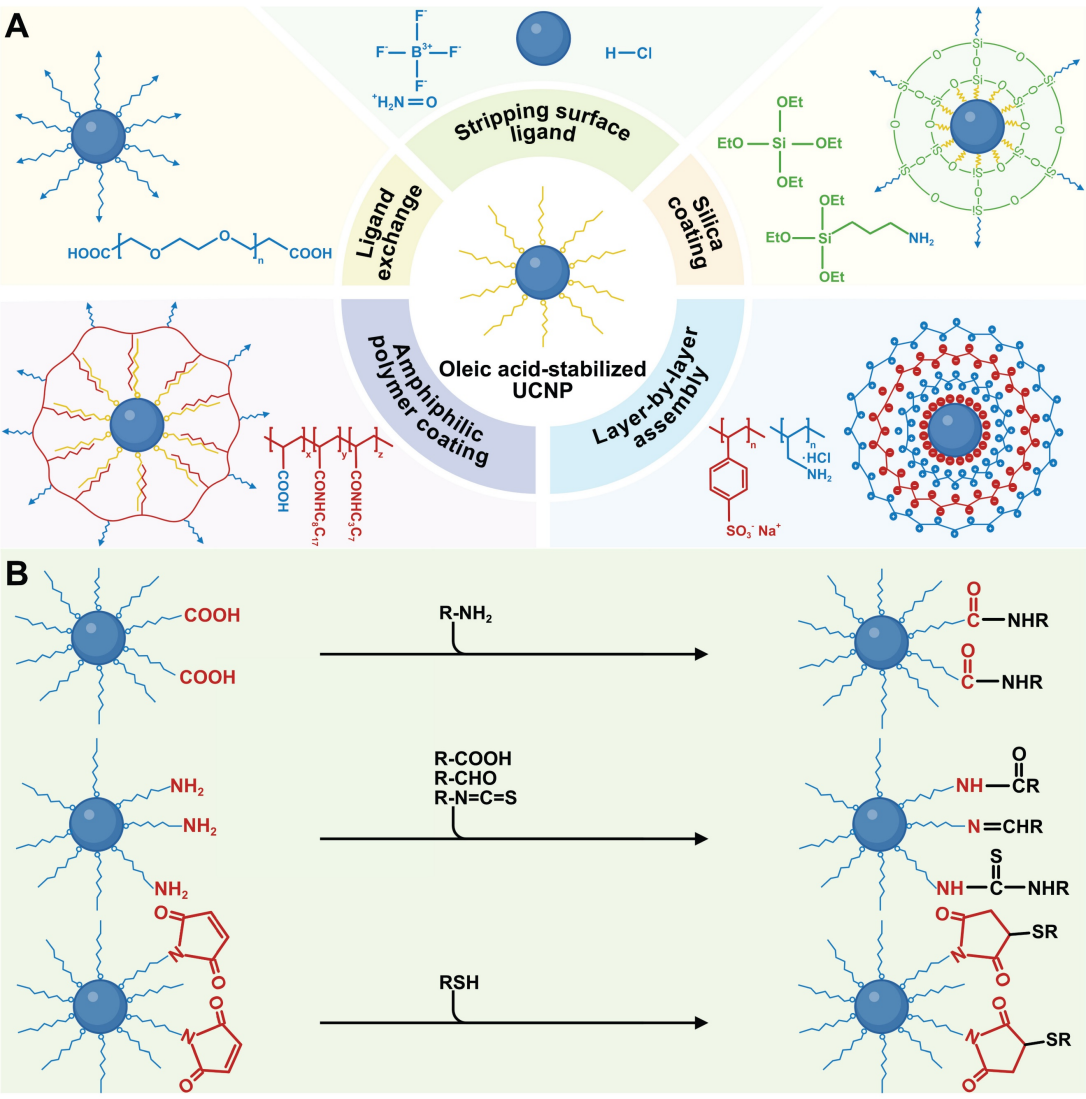
(A) Typical surface modification strategies applicable to UCNPs. (B) Scheme of covalent conjugation of UCNPs with biomolecules. Created with BioRender.com.

The mainstream synthesis methods of UCNPs often use hydrophobic surface ligands, making them soluble in non-polar organic solvents but insoluble in aqueous media, thus restricting their biomedical applications. Surface modification is crucial for enabling UCNPs to disperse in physiological solutions, achievable through techniques such as ligand exchange, stripping surface ligand, silanization, layer-by-layer assembly, and amphiphilic polymer coating (Figure 5A). In these processes, hydrophobic UCNPs are synthesized and modified with hydrophilic functional groups, reducing aggregation in biological environments by altering composition and charge. Additionally, the functional groups provide reactive sites for conjugating with biomolecules, enabling advanced functionalities like active targeting and drug/gene delivery.

### 4.1. Surface modification of UCNPs

#### 4.1.1. Ligand exchange

Ligand exchange improves the water dispersibility of UCNPs by replacing original hydrophobic ligands with functional hydrophilic ones. This process relies on the differing coordination capacities of organic ligands on the material surface, where stronger ligands displace weaker ones. Direct ligand replacement on the particle surface minimizes size changes in UCNPs. For effective exchange in oleic acid-coated UCNPs, multidentate or excess monodentate ligands are required, whereas oleylamine-coated UCNPs allow easier exchange due to weaker ligand interactions 91. Common ligands include small molecules (e.g., citric acid, mercaptopropionic acid, phosphoethanolamine) and polymers (e.g., polyethylene glycol [PEG], polyacrylic acid [PAA], polyethylenimine [PEI]). For *in vivo* applications, bisphosphonate/tetraphosphonate-modified PEG is particularly advantageous, as its hydrophilicity, biocompatibility, and high binding affinity avoid biomolecule interference (e.g., DNA, proteins) 92.

#### 4.1.2. Stripping surface ligand

Stripping surface ligand is a straightforward yet effective strategy for modifying hydrophobic UCNPs. Under ultrasonic conditions, oleate ligands on UCNPs can be removed using NOBF_4_ or HCl. NOBF_4_ substitutes oleate ligands with inorganic BF_4_^-^ anions, enabling UCNPs to disperse in solvents such as acetonitrile, DMSO, and DMF, but not in water 93. Although HCl treatment protonates oleate, causing ligand detachment and leaving UCNPs with bare, positively charged surfaces, it may disrupt the crystal lattice and weaken luminescence 94. The colloidal stability achieved via these two methods is easily disrupted, as the electrostatic balance of the system is sensitive to concentration changes or other ions. Therefore, further modification with hydrophilic molecules or electronegative functional groups (e.g., carboxyl, sulfonic acid, phosphonic acid) is often required, which enhances UCNP stability and luminescence intensity through coordination with RE ions.

#### 4.1.3. Silica coating

Silica exhibits several advantageous properties, including exceptional optical transparency, outstanding chemical stability, and excellent biocompatibility. These attributes have rendered it a Food and Drug Administration (FDA)-approved material for biomedical applications, positioning it as an ideal candidate for the surface modification of UCNPs 95. As previously discussed, depositing SiO_2_ on UCNPs via nonepitaxial growth not only allows precise modulation of emission but also provides abundant functionalizable groups for further modification. The primary silica coating methods are sol-gel nanochemistry in reverse micelles for hydrophobic UCNPs and the modified Stöber method for hydrophilic UCNPs. For example, Veggel *et al.* used the modified Stöber method to synthesize SiO_2_-coated UCNPs and subsequently aminated the silica surface for biological labeling 96. A particularly advantageous variant of SiO_2_ is mesoporous silica (mSiO_2_), which features a high specific surface area and substantial pore volume. These properties enable efficient adsorption and afford high loading capacities for therapeutic agents 97. Notably, silanization may increase particle size and polydispersity, and structural verification of ultrathin silica shells remains technically challenging.

#### 4.1.4. Other methods

Layer-by-layer assembly and amphiphilic polymer coating are additional surface modification methods for UCNPs. This strategy uses electrostatic interactions to sequentially adsorb oppositely charged ligands, enabling the preparation of coated colloids with diverse shapes, sizes, and compositions 98. Amphiphilic polymer coating encapsulates hydrophobic UCNPs with molecules containing hydrophobic alkyl chains and hydrophilic groups, enhancing aqueous dispersion 99. Various amphiphilic polymers have been employed for surface modification, including poly(maleic anhydride-alt-1-octadecene)-bis(hexamethylene)triamine, D-α-tocopheryl polyethylene glycol succinate, block copolymers like poly(ethylene glycol)-block-poly(caprolactone) and poly(ethylene glycol)-block-poly(lactic-co-glycolic acid), as well as non-immunogenic phospholipid membranes 100-102. It is crucial to note that maintaining the colloidal stability of UCNPs in high-ionic-strength biological environments remains challenging, necessitating comprehensive surface modification strategies.

### 4.2. Bioconjugation

Historically, UCNP surface engineering focused primarily on enhancing aqueous dispersion and stability. For theranostic applications, however, bioconjugation with biomolecules is essential. This process is categorized into non-covalent and covalent bioconjugation based on interaction thermodynamics and conjugate stability. Non-covalent approaches, such as electrostatic adsorption, π-π stacking, bioaffinity interactions, or physical entrapment, enable rapid functionalization. Yet relying on weak intermolecular forces (e.g., van der Waals, hydrogen bonding, ionic pairing), they risk dissociation in dynamic biological environments where pH fluctuations, competitive protein adsorption, or shear stress can destabilize complexes, causing premature payload release and off-target effects.

Covalent bioconjugation is a key functionalization strategy ensuring durable, site-specific bioactivity. This approach uses engineered surface groups to form strong covalent bonds with biomolecular functional groups, enabling ordered orientation and robust stability (Figure 5B). Specifically, during the hydrophilic phase transfer of UCNPs, introducing reactive groups (e.g., carboxyl, amino, maleimide) creates a versatile platform for biomolecular conjugation. Carboxyl-based conjugation systems typically employ 1-ethyl-3-(3-dimethylaminopropyl)carbodiimide (EDC)/N-hydroxysuccinimide (NHS) activation chemistry. When ligands like PAA or azelaic acid are anchored to UCNPs, their exposed carboxyl groups are activated by EDC/NHS to generate reactive ester intermediates. These intermediates efficiently couple with primary amine-containing biomolecules such as antibodies and amino-PEG derivatives, forming stable amide bonds 103,104. Amine-functionalized UCNPs prepared with ligands such as PEI or aminoundecanoic acid exhibit dual functionality. The amino groups enable carbodiimide-mediated condensation with carboxylated biomolecules such as folic acid (FA) and aptamer, while the positive surface charge facilitates electrostatic binding to negatively charged nucleic acid 105,106. Maleimide groups, as thiol-specific reactive sites, can be introduced via direct conjugation with maleimide-terminated polymers or secondary modification of amine-functionalized UCNPs with NHS-maleimide derivatives. These groups undergo rapid Michael addition with thiol-containing biomolecules such as cysteine residues or thiolated peptides, forming stable thioether bonds. This strategy is particularly advantageous for site-specific antibody conjugation and protein modifications that preserve structural integrity. Recent advances have integrated novel coupling methods to enhance UCNPs functionalization. For example, copper-free strain-promoted azide-alkyne cycloaddition overcomes the cytotoxicity limitations of traditional click chemistry. Azide-modified UCNPs rapidly conjugate dibenzocyclooctyne-tagged small molecules under physiological conditions, establishing biocompatible fluorescence imaging platforms 107.

## 5. Biological Effects of UCNPs

Compared with bulk materials, nanomaterials exhibit a higher specific surface area and enhanced surface reactivity due to their nanoscale dimensions. These microscopic structural disparities give rise to distinct *in vivo* biological responses 108. Specifically, nanomaterials demonstrate more complex pathways for entering the circulatory system, spatiotemporal tissue accumulation patterns, cellular uptake mechanisms, metabolic clearance kinetics, and biotoxicity profiles compared with conventional materials. The issue of biological effects becomes particularly pronounced in inorganic nanoplatforms such as UCNPs. These particles often accumulate and are poorly degradable *in vivo*, raising concerns about long-term risks. Such risks are not only associated with the materials themselves but also involve nano-biological interactions, including protein corona formation, immune recognition, and biotransformation processes, which remain largely uncharacterized. Therefore, a deep understanding of the biological effects of UCNPs is essential for promoting their safe and efficient translation into practical applications.

### 5.1. Biodistribution

The biodistribution of UCNPs involves a dynamic sequence of events beginning with translocation from administration sites into systemic circulation, followed by tissue accumulation and eventual cellular internalization. This process is influenced by both the administration route and the physicochemical properties of UCNPs. The former critically determines initial deposition and overcomes biological barriers during systemic dissemination, while the latter precisely regulates NP-cell interface interactions, determining endocytic pathway selection, lysosomal escape efficiency, and subcellular localization. Current analytical methods such as inductively coupled plasma optical emission spectroscopy and *in vivo* luminescence imaging have enabled preliminary biodistribution mapping.

#### 5.1.1. Tissue accumulation

**Figure 6:**
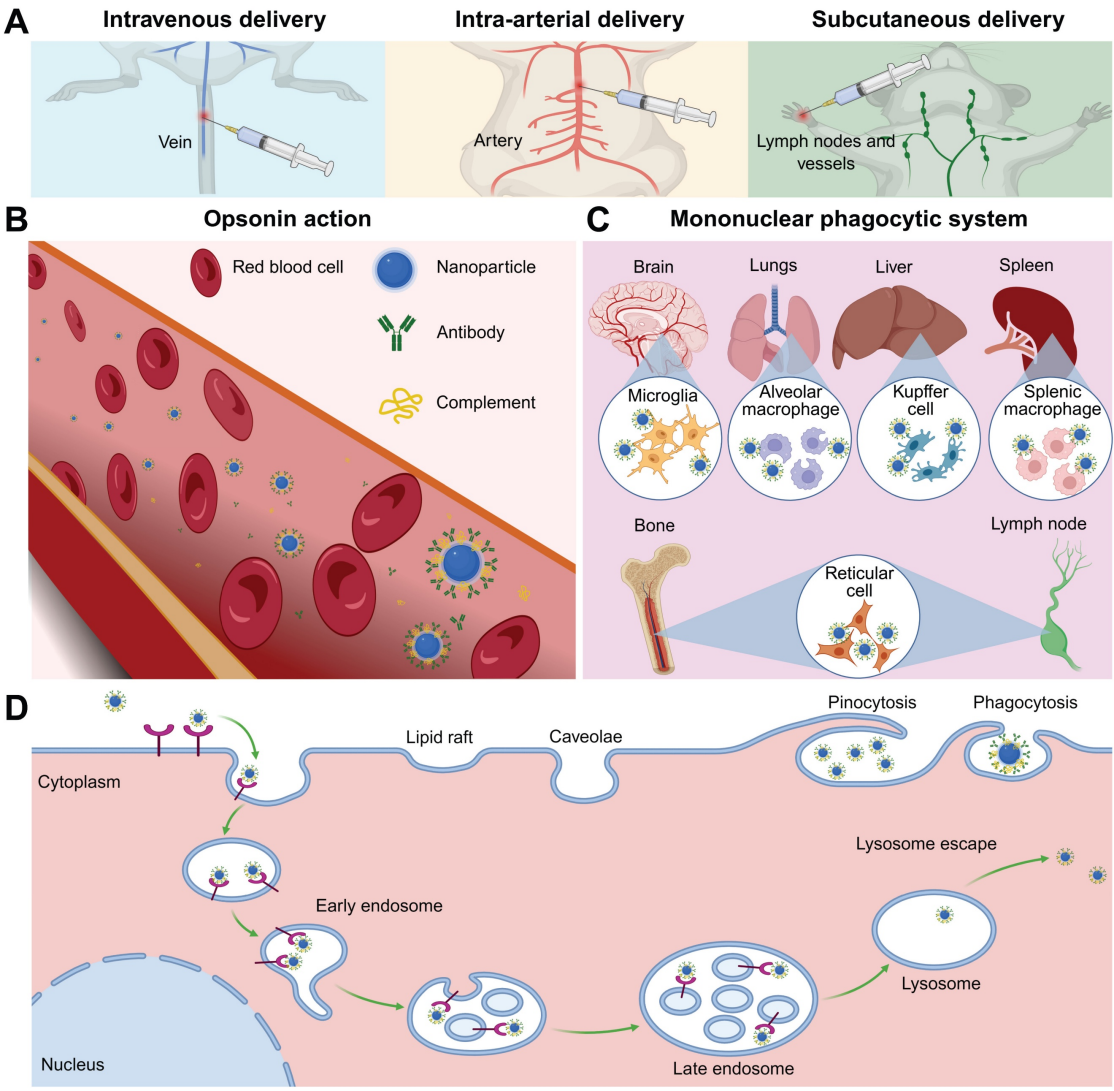
(A) Common delivery strategies. (B) Opsonin action and (C) phagocytosed by mononuclear phagocytic system. (D) Schematic diagram of endocytosis. Created with BioRender.com.

Intravenous administration of UCNPs is the primary method for systemic delivery, allowing direct entry into the bloodstream (Figure 6A). NPs smaller than 500 nm are more suitable for intravenous injection due to their superior circulatory stability and tumor-targeting efficiency 109. Once in the systemic circulation, UCNPs rapidly adsorb plasma proteins to form a dynamic protein corona, which masks surface ligands and redefines NP-receptor binding patterns 110-112. The protein corona consists of opsonins and dysopsonins, affecting NP clearance. Opsonins activate pattern recognition receptors on phagocytes, triggering rapid clearance via the mononuclear phagocytic system, while dysopsonins may delay this process (Figure 6B-C) 113,114. Strategies such as size optimization, charge modulation, and functional coatings can reduce immunogenicity and prolong blood residence time 115-117. For example, ethylenediamine tetramethylenephosphonic acid-modified UCNPs remain in circulation for 60 minutes postinjection, doubling the retention time of citrate-coated counterparts 118. However, repeated administration of polymer-coated NPs may induce adaptive immune responses, accelerating subsequent clearance 119. The tissue accumulation of intravenously administered NPs exhibits distinct organ-specific distribution patterns, which are governed by vascular physiological characteristics and biological filtration mechanisms. The heart and lungs, with high blood perfusion rates, exhibit rapid UCNPs accumulation 120. The blood-brain barrier restricts most NPs from entering neural parenchyma, and muscle tissue penetration is limited by endothelial fenestration size 121,122. The liver and spleen, with discontinuous sinusoidal capillary networks, demonstrate superior NP retention. Quantitative analysis revealed that approximately 87% of UCNPs accumulated in the liver following tail vein injection, highlighting off-target accumulation risks and potential hepatosplenic toxicity at high doses 123.

Intra-arterial delivery is a promising alternative to intravenous delivery for improving tumor therapy outcomes. Following intra-arterial administration, UCNPs enter systemic circulation rapidly, permeate capillary beds, and penetrate targets, increasing their likelihood of reaching the tumor site before hepatic or splenic retention. Preclinical studies in breast and hepatocellular carcinoma models demonstrate 3- to 5-fold increases in tumor accumulation with intra-arterial delivery compared to intravenous routes, coupled with reduced hepatic sequestration 124,125. Unlike intravenous or intra-arterial delivery, subcutaneous administration enables NPs to diffuse through connective tissue and intercellular spaces between capillary or lymphatic endothelial cells, entering systemic circulation via membrane pores. This route enables UCNPs to reach local lymph nodes before entering systemic circulation, prolongs absorption, and reduces the need for repeated administration. Particles sized 10-100 nm and negatively charged are optimal due to weak interactions with blood/lymphatic components and reduced interstitial transport hindrance 126-128. Beyond previously discussed delivery routes, novel approaches such as intraperitoneal injection, oral ingestion, inhalation, or targeted mucosal delivery demonstrate unique potential in ongoing investigations.

#### 5.1.2. Cellular internalization

The initial interaction between NPs and target cells, which involves cellular uptake, is of critical importance for achieving the desired effect. A few NPs can enter cells through simple diffusion or translocation, driven by their concentration and lipophilicity. However, most NPs are internalized via receptor-mediated endocytosis, an energy-dependent process which can be categorized into phagocytosis and pinocytosis (for smaller particles) (Figure 6D) 129. This process initiates with ligand-receptor binding that induces membrane curvature and vesicle encapsulation. Post-internalization, vesicles undergo uncoating and fuse with early endosomes. These compartments employ adenosine triphosphate-driven proton pumps to acidify their lumen, facilitating maturation into late endosomes 130. The late endosome fuses with lysosomes containing specific digestive enzymes, and the interaction between the nanocarrier and the digestive enzymes leads to the degradation of the loaded drugs or nucleic acids. Therefore, the ability of nanocarriers to escape lysosomal degradation is crucial for the delivery of their cargoes. The escape strategies primarily destabilize endosomal membranes by enhancing interactions between the membrane and endosomal solvents. These include using endosomolytic enhancers for direct lysis, pore-forming agents to create transmembrane channels, high pH-buffering agents that mediate the proton sponge effect via protonation-induced swelling, photosensitizers for photochemical disruption, and fusogenic agents to promote membrane fusion by disturbing lipid arrangements 131. Subsequently, the released NPs reach subcellular targets such as the cytoplasm, mitochondria, and nucleus through passive diffusion, membrane potential gradients, targeted signaling, or cytoskeletal transport. Notably, as mitochondrial dysfunction represents a key mechanism of nanomaterial toxicity, many mitochondria-targeting nanoplatforms have been developed to exert antitumor therapeutic effects.

The cellular dynamics of UCNPs adhere to universal principles and are primarily governed by their specific physicochemical parameters. Hydrodynamic diameter dictates endocytic route selection: smaller UCNPs prefer clathrin-mediated pathways, while larger aggregates may activate phagocytic mechanisms 132,133. Surface charge emerges as a pivotal determinant of uptake efficiency, where positively charged UCNPs exhibit enhanced membrane affinity through electrostatic interactions with the anionic glycocalyx, facilitating rapid receptor clustering and accelerated vesicle formation compared to their neutral or negatively charged counterparts 134-136. In addition, Li *et al.* reported that positively or neutrally charged UCNPs were internalized by most cell lines, whereas negatively charged UCNPs were primarily internalized by cancer cell 137. The size- and charge-dependent cellular uptake can be further modulated by surface ligands. For example, PEG functionalization sterically hinders nonspecific interactions, whereas targeted ligands promote receptor-mediated trafficking, thereby enhancing the precision of cellular entry. Furthermore, concentration-dependent uptake kinetics demonstrate a linear correlation between UCNPs dosage and intracellular accumulation within biocompatible ranges, though supraoptimal concentrations risk lysosomal overload and subsequent organelle stress. Post-internalization fate studies emphasize the key role of lysosome escape efficiency, with UCNPs engineered with pH-responsive polymer coatings, hydroxychloroquine, or cell penetrating peptides enhancing cytosolic delivery by destabilizing the lysosome membrane 138-140.

### 5.2. Excretion

Pharmaceutical agents must meet strict clearance criteria, especially diagnostic agents, which require complete elimination within defined timeframes. This is because systemic clearance kinetics directly affect drug exposure duration, a key factor in determining toxicological risks. Similar to conventional pharmaceuticals, preclinical studies show that UCNPs are primarily cleared via hepatobiliary and renal pathways. Their excretion routes are primarily determined by hydrodynamic diameter due to the size-selective filtration mechanisms inherent in hepatic and renal tissues. Consequently, contemporary engineering strategies emphasize the development of biodegradable UCNPs, which facilitate size reduction from their original dimensions to clearance-compatible fragments.

#### 5.2.1. Renal excretion

**Figure 7:**
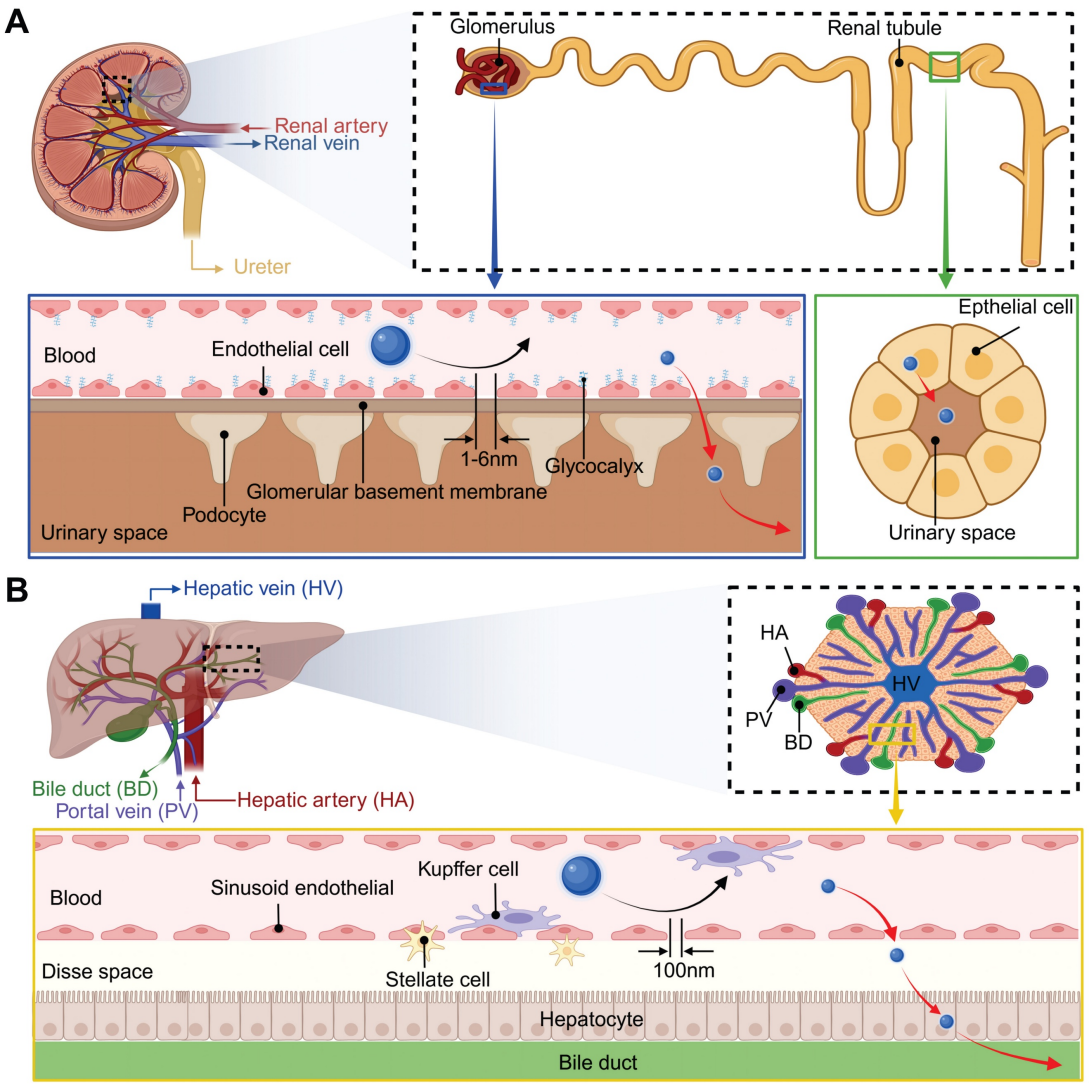
Size-dependent excretion of NPs in the kidneys and liver. (A) Kidney structures and excretion of NPs in the kidney. (B) Liver structures and excretion of NPs in the liver. Created with BioRender.com.

Renal excretion is the optimal route for NPs clearance, as it minimizes retention and cytotoxicity by avoiding intracellular catabolism. The fundamental structural and functional unit of the kidney is the renal unit, which is primarily composed of glomeruli and tubules. As illustrated in Figure 7A, the kidney filtration threshold for hard NPs is approximately 6 nm or smaller, taking into account the cumulative effect of the multilayer structures of the glomeruli 141-143. Experimental evidence indicates that rigid NPs exceeding 100 nm are largely excluded from urinary excretion due to their inability to penetrate the endothelial fenestrae. NPs in the 6-100 nm range may traverse the endothelial layer but are subsequently retained by the glomerular basement membrane, while those between 1-6 nm demonstrate progressive filtration efficiency inversely proportional to size. Utilizing gamma counter detection, PEG-modified, ^153^Sm-labeled UCNPs (<10 nm), administered intravenously, were observed to accumulate in the bladder at a concentration of 5.28±0.2% ID g^-1^ from 0.5 to 6 hours post-injection 144. In another study, Cao *et al.* found that within 48 hours, approximately 6.32±0.51% of the injected UCNPs were excreted in the urine 145. Notably, NPs smaller than 1 nm may exhibit delayed clearance due to transient interactions with the glomerular glycocalyx. Soft NPs exhibit enhanced filtration capacity owing to structural deformability, elevating the renal filtration threshold to approximately 10 nm 146. Moreover, aside from being filtered through the glomerulus, a small number of NPs are secreted and reabsorbed by the renal tubules 147.

#### 5.2.2. Hepatobiliary excretion

In addition to renal excretion, the hepatobiliary system constitutes an alternative pathway for the elimination of NPs, albeit with a comparatively slower rate. The liver is supplied with blood by the hepatic artery and portal vein, which then circulate the blood internally before releasing it into the systemic circulation via the hepatic vein (Figure 7B). Metabolic waste and secreted substances from liver cells are transformed into bile, which is subsequently stored in the gallbladder and ultimately eliminated via the digestive tract. Key metabolic activities and substance exchanges occur within the hepatic sinusoids, a critical region where hepatocytes, Kupffer cells, and liver sinusoidal endothelial cells collaborate closely to maintain liver function 148. During hepatic filtration, liver sinusoidal endothelial cells form the principal size-selective barrier through their characteristic 100-150 nm fenestrations. NPs larger than 100 nm typically cannot traverse these fenestrae to reach hepatocytes for hepatobiliary clearance, leading to prolonged retention by Kupffer cells and impaired fecal excretion 149. Conversely, NPs with a size ranging between 6 and 100 nm are able to pass through the fenestrae of sinusoidal endothelial cells, interact with liver cells, and be excreted into the feces 148. Many studies have demonstrated that UCNPs exceeding kidney filtration thresholds predominantly accumulate in hepatic and splenic tissues post-intravenous administration, followed by gradual hepatobiliary excretion. For instance, tracking of 11.5 nm PAA-coated UCNPs revealed delayed intestinal signals emerging at 7 days post-injection, peaking at 90 days, and diminishing to near-background levels by day 115 120. Longitudinal metabolic investigations further identified extended biliary excretion cycles lasting up to 214 days for certain NPs, with excretion efficiency markedly lower than that of renal clearance pathways 150.

#### 5.2.3. Biodegradable UCNPs

Considering that the elimination of NPs is highly size-dependent, biodegradable UCNPs have attracted increasing research attention. For example, Hong *et al.* fabricated biodegradable red-emitting K_3_ZrF_7_:Yb,Er NPs using an enhanced high-temperature coprecipitation approach. These NPs can be fully converted into approximately 5 nm residues within 10 hours when exposed to water, with a pH-dependent degradation rate that is faster in strong acids or alkalis and slower in weakly acidic biological solutions. *In vivo* experiments revealed that subcutaneously injected UCNPs were biodegraded within 120 minutes in both normal and 4T1 tumor-bearing mice, with a slower degradation rate in the tumor microenvironment (TME) than in normal physiological conditions, providing a time window for imaging. Additionally, these NPs showed low *in vitro* toxicity to human embryonic lung fibroblasts and no significant *in vivo* toxicity to muscles and major organs, with degradation products quickly excreted from rats 151. Analogously, Lv *et al.* synthesized degradable peptide-modified UCNPs via a double emulsion method, which degrade into NPs smaller than 6 nm under weakly acidic conditions 152. Furthermore, engineered surface shell modifications have emerged as a novel approach to regulate UCNPs degradation dynamics. For instance, Lin *et al.* constructed TME-responsive silica shells through Mn-O bond integration, where this stimuli-degradable architecture enables not only precision drug release but also enhances intratumoral penetration via progressive size diminution, substantially improving the spatial delivery efficiency of chemotherapeutic agents 153.

### 5.3. Biotoxicity

As early as 2003, Service conducted a comprehensive analysis of the biotoxicity of nanomaterials and their potential environmental impact in the journal Science 154. This topic has subsequently attracted significant attention and in-depth discussion within the academic community. However, a 2018 study by Oliveira *et al.* revealed that only 18% of 1,811 publications focusing on biomedical applications of UCNPs addressed toxicity concerns, thereby underscoring a gap in risk assessment research 155. Available studies indicate that the biotoxicity of UCNPs is governed by their physicochemical properties and exposure conditions. Most UCNPs exhibit acceptable biosafety profiles within specified concentration ranges but may induce dose-dependent organ-specific toxicity under high-dose or prolonged exposure conditions. Given the complexity of nanomaterials, related assessments should be conducted on a case-by-case basis.

#### 5.3.1. Multilevel biotoxicity manifestations of UCNPs

The biotoxicity of UCNPs can be systematically evaluated through a hierarchical research framework spanning multiple biological complexity levels. At the cellular level, UCNPs exhibit dose- and time-dependent cytotoxicity, primarily mediated by mitochondrial dysfunction and oxidative stress. Standardized cytotoxicity assays, including the methyl thiazolyl tetrazolium, 3-(4,5-dimethylthiazol-2-yl)-5-(3-carboxymethoxyphenyl)-2-(4-sulfophenyl)-2H-tetrazolium inner salt, and CCK-8 assays, demonstrate that engineered UCNPs maintain high cellular viability under acute exposure conditions. Metabolic impairment becomes statistically significant only beyond critical dose thresholds, as summarized in Table 2. Caenorhabditis elegans exhibit remarkable tolerance to UCNPs, showing no significant physiological or reproductive impairments following direct ingestion 156-158. In contrast, zebrafish embryos exposed to high concentrations of uncoated UCNPs develop teratogenic effects, including delayed hatching, axial malformations, and gut dysbiosis, highlighting developmental stage-specific vulnerabilities 159-161. In rodent studies, UCNPs administered via various administration routes have demonstrated acceptable safety profiles across diverse sizes, charge, and surface modifications (Table 3). Extended observation periods, ranging from minutes to months, and multiple dose levels reveal no significant toxic effects in behavioral parameters, body weight fluctuations, tissue morphology, or blood biochemical indices 162-166. Transmission electron microscopy analysis of PEGylated UCNPs in mouse feces reveals no alterations in morphology, size, or distribution, suggesting that these NPs do not undergo significant *in vivo* transformation 167. However, high-dose administration may induce target-organ toxicity, manifesting as hepatic steatosis, pulmonary inflammation, and renal tubular necrosis 168. Regarding RE derivatives, chloride and nitrate salts exhibit reduced acute toxicity but pose risks of pneumonia and acute inflammatory responses with prolonged exposure 169,170. Lanthanide chlorides enhance vascular permeability and influence blood components and enzymatic activities, though they have minimal impact on spermatogenesis. Notably, offspring of animals exposed to lanthanide citrates during lactation or pregnancy exhibit reduced body weight and excretory dysfunction, albeit without observable teratogenic effects 171.

#### 5.3.2. Mechanism and influencing factors of biotoxicity

The biotoxicity of UCNPs arises from their interactions with cellular components, triggering pathological cascades through distinct molecular pathways. A principal cytotoxic mechanism involves reactive oxygen species (ROS) generation mediated by surface redox activity and lanthanide ion leaching 172,173. Acute ROS mediate oxidative damage via DNA strand breaks, lipid peroxidation, and protein denaturation, while chronic overproduction triggers apoptosis/autophagy. Lysosomal dysfunction constitutes another pivotal toxicity pathway. For instance, UCNPs suppress autophagic lysosome reformation through dual disruption of phosphatidylinositol signaling and clathrin-mediated membrane recruitment, causing cellular dysfunction and inflammatory activation 174. Furthermore, lysosomes provide critical microenvironmental conditions triggering degradation-dependent nanotoxicity. Under lysosomal acidity, progressive UCNPs dissociation releases lanthanide ions that form needle-like crystalline precipitates with phosphates. These rigid nanostructures mechanically pierce lysosomal membranes and adjacent organelles, initiating cascading cellular damage. It should be noted that appropriate coatings, such as block copolymers, phosphonates with multiple phosphonic groups, silica, and phosphate, can mitigate the risks of chemical dissolution 155. Furthermore, Gd^3+^-doping systems, commonly used for imaging contrast, are associated with phosphate-associated nephrogenic systemic fibrosis through renal ion retention 175.

The biotoxicity of UCNPs is primarily determined by three interconnected physicochemical factors: size dynamics, surface charge regulation, and ligand-mediated interfacial interactions. Size significantly influences cellular uptake efficiency and elimination kinetics. UCNPs with diameters above 55 nm exhibit minimal interference with cell proliferation but may induce prolonged inflammatory responses due to extended tissue retention 176. Conversely, smaller UCNPs demonstrate enhanced cellular penetration and pronounced suppression of tumor cells 177. Clearance studies reveal that 35 nm UCNPs are eliminated faster than 55 nm counterparts, despite higher cellular internalization rates 172. Surface charge plays a critical role in nano-biological interactions. Positively charged UCNPs exhibit superior cellular internalization via clathrin-mediated endocytosis, driven by electrostatic attraction to anionic cell membranes 157. However, such charge-driven uptake often correlates with membrane destabilization, as evidenced by the greater membrane disruption caused by cationic PEI-coated UCNPs compared to anionic variants 178. Ligand chemistry significantly impacts biocompatibility by modulating surface charge, hydrodynamic stability, and targeting specificity. For instance, PEG-based coatings may induce sub-lethal cellular stress, which manifests as nuclear morphological alterations and nucleolar protein dysregulation, while PEI or polymaleic anhydride-olefin modifications can trigger calcium signaling disruption and cell death 179,180. Chitosan reduces lipid emulsification and enhances platelet adhesion, while silica interacts with cells to potentially contribute to autoimmune diseases such as rheumatoid arthritis, lupus, or chronic renal conditions 181-183. Additionally, ligand degradation byproducts, such as acidic PEG derivatives, may lead to acidosis and hypercalcemia 184.

## 6. Tumor Targeting

The accumulation of therapeutic agents in tumors represents a key to achieving theranostic objectives. However, the intrinsic physicochemical properties of pharmaceutical agents and biological barriers often significantly restrict their biodistribution 185. Non-specific drug deposition in normal tissues may lead to severe side effects. In this context, nanocarrier-based targeting strategies have emerged as promising approaches to significantly improve tumor-specific drug accumulation, enhance pharmaceutical stability, and facilitate cellular uptake, thereby providing safer and more effective options. Based on different mechanisms, targeting strategies can be broadly categorized into passive targeting and active targeting.

### 6.1. Passive targeting

**Figure 8:**
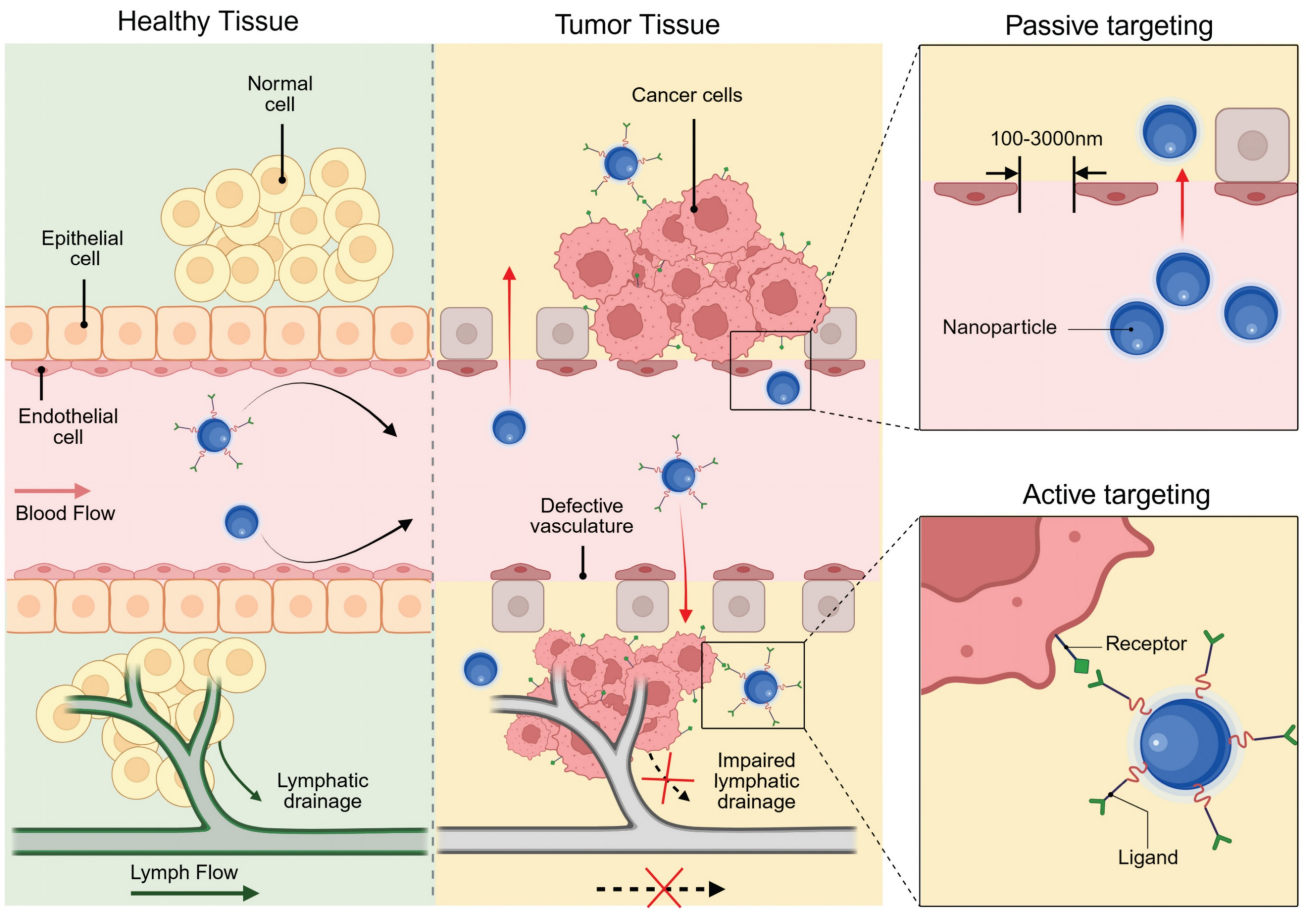
Passive and active targeting mechanism of NP on cancer cells. Created with BioRender.com.

The tumor-targeting ability of nanomedicine has traditionally been ascribed to the enhanced permeability and retention (EPR) effect. First described by Yasuhiro Matsumura and Hiroshi Maeda in 1986, the EPR effect refers to the phenomenon whereby drugs accumulate in tumors solely through the utilization of tumor pathophysiological characteristics 186. Specifically, in contrast to normal tissues, tumors exhibit more abundant blood vessels with distinct structural characteristics, which result from rapid cell proliferation and heightened demands for oxygen and nutrients. As shown in Figure 8, these abnormal blood vessels feature a discontinuous endothelial lining, which generates relatively large pores (0.1-3 μm) compared to those in normal vasculature 187. Additionally, the lack of lymphatic vessels within tumors disrupts the normal drainage of lymphatic fluid. Consequently, liposomes, NPs, and macromolecular drugs smaller than the pore diameter can penetrate the vessel wall, infiltrate the tissue, and accumulate in tumors. The above process is termed passive targeting because it does not rely on active recognition motifs for targeting.

Although the EPR effect is a universal pathophysiological phenomenon observed in rodent, rabbit, canine, and human solid tumors, only a few anticancer drugs using passive targeting have been approved clinically in 30 years 188,189. Notably, Stefan Wilhelm *et al.*'s 2016 meta-analysis revealed that NPs accumulation in the tumor constitutes only 0.7% of the injected dose 190. In fact, tumor cells are embedded within a complex TME, which is composed of cancer-associated fibroblasts, immune cells, endothelial cells, extracellular matrix and other components. These components are highly variable, and each of them poses a potential barrier to the targeting 185. For example, rapid tumor cell proliferation, hyperpermeable vasculature, and impaired lymphatic drainage collectively elevate interstitial pressure, creating hydrodynamic resistance that limits NPs penetration into high-pressure regions. Compounding this barrier, the fibrous extracellular matrix architecture physically obstructs particle diffusion while its abnormal proliferation compresses blood and lymphatic vessels. This compression establishes a self-reinforcing cycle of increasing interstitial hypertension that progressively restricts NPs transport across tumor 191,192. Notably, physical and pharmacological strategies have emerged to improve passive tumor targeting. Physical approaches, such as hyperthermia, ultrasound, and microbubbles, can enhance the accumulation of nanomedicines in tumors 193. Pharmacological approaches include increasing blood pressure via angiotensin, promoting vascular permeability using tumor necrosis factor and NO/CO-generating agents, and inhibiting cancer-associated fibroblasts with drugs like losartan 194-198. Physical priming allows local control of treatment parameters but is only suitable for locally confined diseases. Pharmacological priming is more applicable to systemic diseases, yet has suboptimal control over spatial and temporal treatment parameters.

### 6.2. Active targeting

Active tumor targeting represents an approach that leverages specific molecular recognition mechanisms to precisely deliver theranostic agents. The core principle involves leveraging overexpressed biomarkers on tumor cells or in the TME for localized nanomedicine enrichment via ligand-receptor interactions (Figure 8) 199. This strategy dates back to 1980, when Lee D. Leserman *et al.* covalently conjugated liposomes with functional proteins (monoclonal antibodies and Staphylococcus aureus protein A) for specific cell binding 200. Subsequent studies further expanded the repertoire of ligand-receptor pairs and diversified the nanocarriers. The surface chemistry programmability of UCNPs provides a versatile platform for active targeting through ligand bioconjugation, which can be implemented via non-covalent adsorption or covalent binding mechanisms.

The heterogeneity of BC necessitates tailored targeting strategies aligned with distinct receptors in luminal, HER2-positive, and TNBC subtypes. For HER2-enriched BC, full-length antibodies (e.g., trastuzumab) or peptides functionalize UCNPs, leveraging high-affinity binding to facilitate tumor accumulation 201,202. However, conjugating macromolecular antibodies to comparably-sized NPs compromises pharmacokinetics and colloidal stability 203. Alternative strategies using smaller antibody fragments, like single-chain variable fragments, offer improved pharmacokinetics and tumor penetration while retaining high affinity 204-206. Similarly, designed ankyrin repeat proteins provide comparable affinity with greater compactness and cost-effectiveness for HER2-targeted UCNPs. For instance, UCNPs doped with radioactive ^90^Y and conjugated to the HER2-targeting toxin DARPin-PE40 have demonstrated approximately 2200-fold enhanced synergistic cytotoxicity in HER2-positive BC cells 207. In luminal subtypes with elevated neuropeptide Y_1_ receptor expression, Yu *et al.* developed UCNPs functionalized with Y_1_-specific ligands for selective accumulation 168. For target-lacking TNBC, small-molecule ligands exploit dysregulated metabolic pathways or membrane transporter activation to achieve targeted delivery. For instance, folate receptor (FR) overexpression in certain TNBC cases enables FA-conjugated UCNPs to efficiently accumulate in tumor cells through receptor-mediated endocytosis 208. Moreover, peptide-functionalized nanoprobes enable active TNBC targeting through specific binding to oncogene product receptors 152. In addition to the traditional single-level targeting approach, hierarchical targeting systems based on different ligands can enhance tumor specificity and therapeutic efficacy by binding to cell surface receptors and organelles 209. Furthermore, considering the dynamic nature of the TME, static ligand design often fails to maintain sustained binding ability, prompting researchers to shift from single ligand optimization to systematic engineering of ligand-carrier-microenvironment interactions 210.

## 7. UCNPs for BC Molecular Imaging

Early diagnosis is critical for improving the prognosis of BC patients. Clinical data show that patients diagnosed early achieve five-year survival rates exceeding 99%, compared to less than 30% for advanced-stage disease 211. This stark disparity underscores the urgent need to overcome diagnostic technology bottlenecks. Conventional imaging primarily detects anatomical discrepancies but remains limited in identifying submillimeter early-stage lesions due to restricted spatial resolution. Furthermore, these techniques require acquisition times from minutes to hours and cannot provide real-time feedback on dynamic disease progression. In contrast, molecular imaging synergizes structural visualization with real-time mapping of molecular and cellular processes, offering high sensitivity, rapid response kinetics, and superior spatiotemporal resolution 212. Among the emerging tools in this field, UCNPs have emerged as promising agents due to their unique optical properties and nanoscale dimensions. The following will respectively elaborate on the development of molecular imaging based on UCNPs in BC diagnosis (Table 4).

### 7.1. Fluorescence imaging

Fluorescence imaging is an optical imaging technique that detects fluorescence emitted by objects under illumination with light of specific wavelengths. Engineered UCNPs utilizing NIR excitation sources exhibit exceptional potential in fluorescence imaging due to their strong tissue penetration, minimal autofluorescence interference from biological tissues, and negligible phototoxicity.

In 2008, Chatterjee *et al.* first achieved *in vivo* animal imaging using PEI-coated NaYF_4_:Yb,Er NPs, demonstrating the superiority of UCNPs over quantum dots in deep-tissue imaging 213. Subsequent engineering advancements, including optimized surface modifications and targeted ligand conjugation strategies, have further enhanced their utility for precise BC imaging applications. For instance, HER2 represents a pivotal biomarker and therapeutic target associated with tumor aggressiveness and poor prognosis, with overexpression observed in 10% to 30% of BC 214,215. Grebenik *et al.* developed a HER2-specific imaging probe by conjugating UCNPs with mini-antibodies, achieving tenfold greater signal intensity in SK-BR-3 cells than control groups. This system demonstrated tumor visualization at 1.6 mm depth in simulated tissues with a 4.5 signal-to-noise ratio, while theoretical models predicted detection capabilities extending to 4 mm depths 206. In two additional studies, Panikar *et al.* and García *et al.* respectively employed confocal microscopy to visualize anti-HER2 ligand-conjugated NaYF_4_:Yb,Er NPs, demonstrating specific intracellular red fluorescence in SK-BR-3 cells under 980 nm excitation 202,216.

The FR, a cell membrane receptor for FA, is often overexpressed in ovarian, lung, breast, and hematopoietic myeloid cancers. Because the FR is either absent from normal tissues or localized to the apical surfaces of polarized epithelia (where it is inaccessible to circulating drugs), FA-linked drugs do not normally accumulate in healthy tissues 217. However, due to its full accessibility on tumor cells, the FR has been widely exploited as a target, particularly in TNBC 218. For example, FA-modified UCNPs can specifically target and bind to overexpressed FR on BC cell surfaces, enabling efficient internalization and exhibiting strong fluorescent signals in MCF7, MDA-MB-231, and MDA-MB-468 cells 219-222. As another critical target, integrins play a role as indicators of metastasis. Their diagnostic significance is particularly pronounced in BC bone metastasis detection, where osteoclast-specific overexpression of αvβ3 integrin within osteolytic lesions provides unique pathological signatures. Building upon this mechanism, investigators developed arginine-glycine-aspartic acid peptide-conjugated UCNPs as advanced nanotheranostic probes. These engineered NPs demonstrated rapid tumor targeting within 1 hour post-administration, maintaining a robust tumor-to-background ratio of 24 throughout the 24-hour monitoring period. Notably, their tissue penetration depth reached an unprecedented 600 μm, representing a threefold improvement over conventional visible-light imaging agents 223.

**Figure 9:**
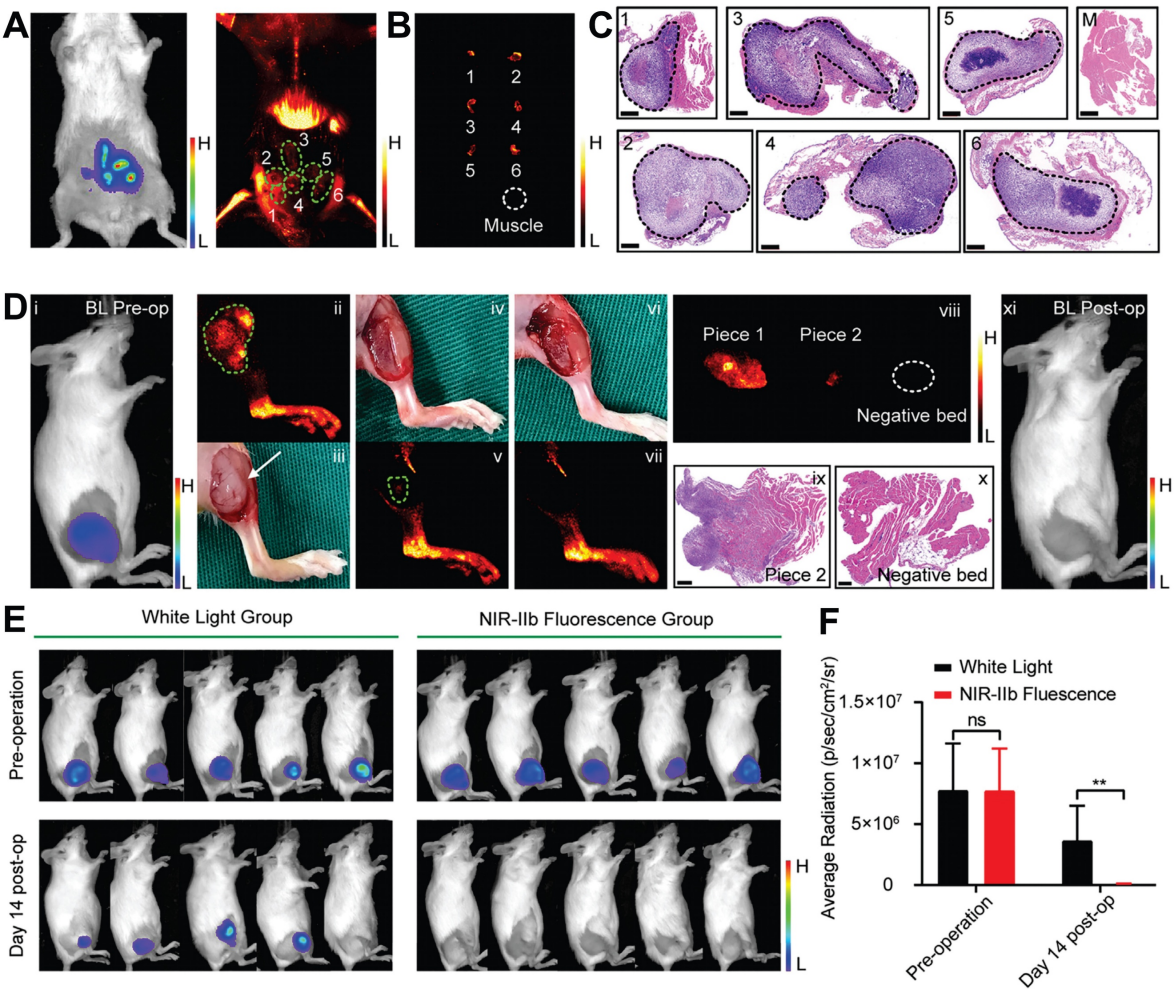
The identification and surgery of tumors in tumor-bearing mice under NIR-IIb fluorescence imaging navigation. (A) Representative bioluminescence (left) and NIR-IIb (right) images of a multiple microtumor mouse model. (B) *Ex vivo* NIR-IIb fluorescence imaging of resected pieces. (C) H&E staining of resected pieces. (D) Representative images during NIR-IIb fluorescence-guided surgery. (E) Bioluminescence images of mice before and 14 days after surgery under white-light (left) and NIR-IIb fluorescence guidance (right). (F) The corresponding average radiation of region of interest. Adapted with permission from 224, copyright 2022, under a Creative Commons CC BY license.

Furthermore, addressing the critical limitation of tissue penetration depth in current intraoperative near-infrared-I (NIR-I, 700-900 nm) fluorescence imaging for BC, Zhang *et al.* advanced the field by engineering integrin-targeted NPs optimized for near-infrared-IIb (NIR-IIb, 1500-1700 nm) emission (Figure 9). This nanoplatform demonstrated remarkable tissue penetration in the NIR-IIb region, with its signal-to-background ratio at 5 mm depth fourfold higher than that of the clinical reagent indocyanine green. Additionally, NIR-IIb imaging showed significantly enhanced signal-to-background ratio in lymph node visualization, enabling high-resolution angiography of brain and hindlimb vessels with narrower vascular profiles and superior contrast. In transgenic MMTV-PyVT mice, the nanoprobe discriminated malignant from normal tissues with an area under the receiver operating characteristic curve of 0.89, achieving 93.8% sensitivity and 79.4% specificity. Notably, across multiple tumor models, the platform precisely targeted tumors, identified microtumors as small as 2 mm, and guided complete resection. In an intramuscular tumor-invasion model, NIR-IIb imaging-guided surgery eliminated tumor recurrence at 14 days, whereas 4/5 mice in the white-light group exhibited recurrence. This technology presents a novel solution to enhance surgical precision and reduce positive margin rates in BC resection 224.

### 7.2. Dual-modal/multimodal imaging

Single-modal imaging technologies typically capture only single-type information within biological systems. To address this limitation and acquire comprehensive data, dual-modal/multimodal imaging techniques have been developed. Introducing multifunctional moieties into the lattice or surface of UCNPs extends their application beyond fluorescence imaging, encompassing modalities such as magnetic resonance imaging (MRI), computed tomography (CT), and positron emission tomography (PET). This integration leverages complementary detection principles to synergistically enhance imaging contrast while reducing the dosages of contrast agent (CA).

**Figure 10:**
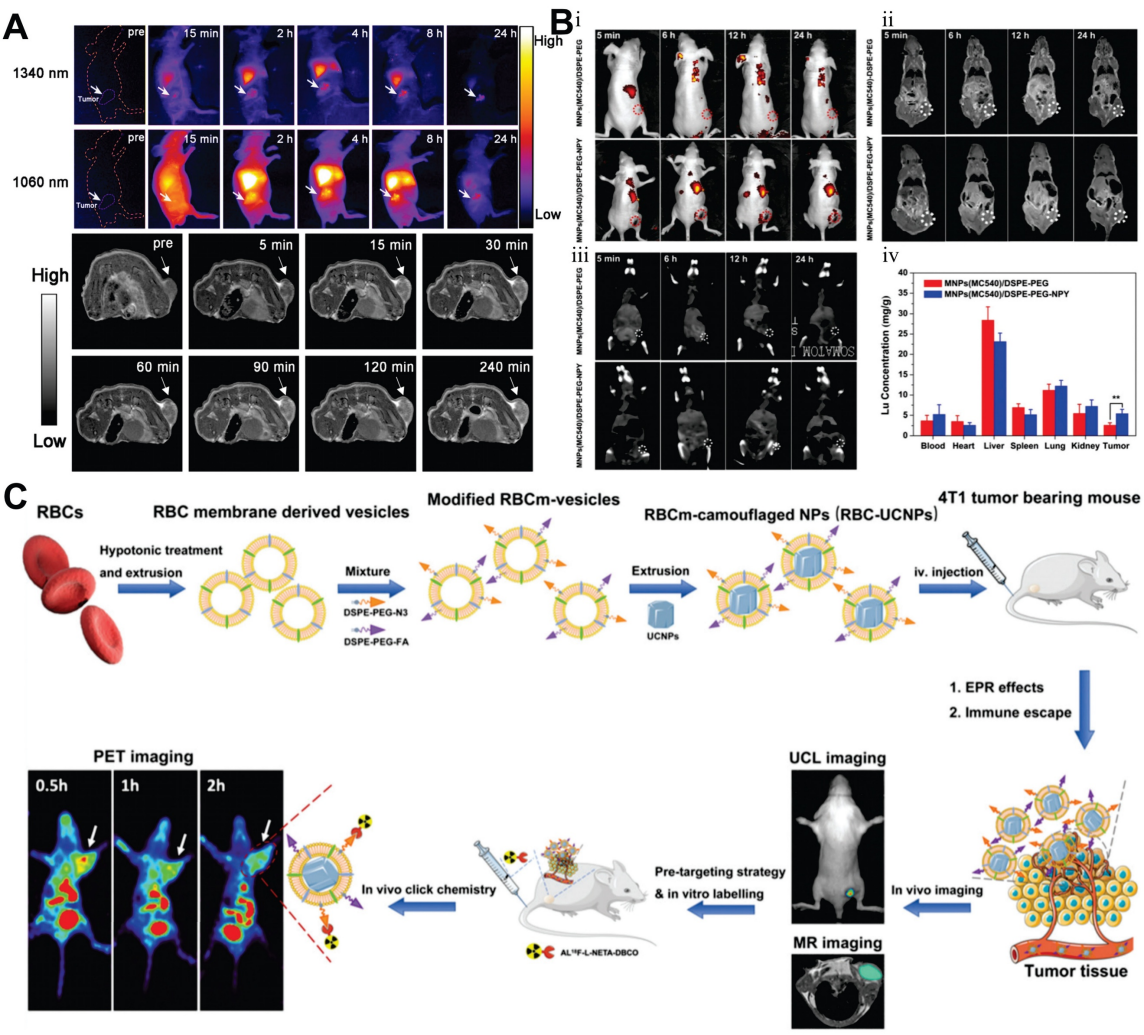
(A) *In vivo* dual-modal imaging based on the NPs. NIR-II fuorescence images of 4T1-tumor-bearing mice at 1060 and 1340 nm after NPs administration (top); T1-weighted MRI of breast tumor after injection with NPs for 0, 5, 15, 30, 60, 90, 120, and 240 min. The tumor area was marked with arrow (bottom). Adapted with permission from 226, copyright 2021, under a Creative Commons CC BY license. (B) *In vivo* multimodality imaging and biodistribution of Y_1_Rs-ligand-functionalized nanocomposites. (i) UCL, (ii) MRI, and (iii) CT imaging of MCF-7 tumor-bearing nude mice after the intravenous injection of nanocomposites at 5 min, 6 h, 12 h, and 24 h; (iv) biodistribution of nanocomposites in the main organs of MCF-7 tumor-bearing mice at 12 h post-injection. Adapted with permission from 168, copyright 2018, Royal Society of Chemistry. (C) The schematic of the preparation process of the modified RBC-UCNPs and their applications of MRI, UCL imaging and PET imaging in TNBC bearing mice. Adapted with permission from 208, copyright 2020, Royal Society of Chemistry.

MRI is one of the most sensitive modalities for BC detection, particularly for identifying occult lesions and preoperative evaluation of breast-conserving surgery. However, the commonly used MRI CA gadolinium diethylenetriamine pentaacetate suffers from rapid clearance and low relaxivity, requiring high doses that increase biological burden 225. Paramagnetic RE ions Gd^3+^ and Eu^2+^ are commonly used as T1 CA, while Dy^3+^ and Ho^3+^, due to their large magnetic moments, are typically employed as T2 CA. For example, Wei *et al.* developed NaGdF_4_:Nd@NaLuF_4_ NPs for dual-modal imaging of TNBC (Figure 10A). Under 808 nm laser excitation, the probe exhibited strong fluorescence emissions at 1060 nm and 1340 nm, with a tumor-to-background ratio of 8.2 at 1340 nm. It enabled rapid hepatobiliary clearance with a 15.8 h half-life in the liver, avoiding long-term retention in the reticuloendothelial system. *In vivo* experiments showed the fluorescence signal peaked at 4 h post-injection, and 4T1 tumor MRI signals were enhanced by 1.46-fold at 240 min, enabling precise BC detection and boundary delineation 226. However, RE incorporation into MRI may compromise magnetic field exchange efficiency, limiting image enhancement 227. This has spurred interest in transition metal ion-based nanomaterials, which exhibit paramagnetic, superparamagnetic, or ferromagnetic properties coupled with favorable biocompatibility. Additionally, the integration of magnetic materials with UCNPs yields dual-modal imaging composites, significantly enhancing T_2_-weighted imaging performance 228-231.

Recently, multimodal imaging combining UCL/MRI/CT or UCL/MRI/PET has gained traction, integrating advantages to improve spatial resolution, sensitivity, and diagnostic efficiency. CT plays a pivotal role in evaluating lymph node status and metastases in BC via high-resolution anatomical imaging based on tissue-specific X-ray attenuation 232. While iodinated CA remain clinical standards for safety and cost, their limited radiodensity has spurred the development of high-atomic-number element-based UCNPs. 233. For example, Yb demonstrates superior X-ray attenuation capabilities with an absorption coefficient of 3.88cm^2^·g^-1^ at 100 keV, significantly surpassing Gd (3.11cm^2^·g^-1^) and doubling the radiodensity of iodine-based agents (1.94 cm^2^·g^-1^). As a sensitizer, Yb^3+^ enhances the emission of UCNPs via its large NIR absorption cross-section. In 2018, Yu *et al.* synthesized Y_1_ receptor ligand-modified LiLuF_4_:Yb,Er@nLiGdF_4_@mSiO_2_ nanocomposites for trimodal BC imaging (Figure 10B). The LiLuF_4_:Yb,Er core generated UCL under 980 nm laser excitation, while Gd^3+^ ions in the shell enabled MRI with a longitudinal relaxivity of 10.24 Mm^-1^·s^-1^. The high atomic numbers of RE ions (Lu^3+^, Yb^3+^, Gd^3+^) enabled CT imaging, with CT values linearly correlating with nanocomposite concentrations. Animal studies demonstrated that Y_1_ receptor ligand modification facilitated targeting of Y_1_ receptor-overexpressing BC, with gradually enhanced UCL and MRI signals in tumor sites post intravenous injection. The accumulation of nanocomposites in MCF-7 tumors was twice that of the unmodified group 168.

PET, a molecular nuclear medicine modality, assesses distant organ metastases in BC but suffers from suboptimal spatial resolution due to limitations in conventional scintillation detectors. Moreover, existing radionuclide labeling strategies also face challenges like harsh conditions, multi-step protocols, and low yields 234. Leveraging the strong adsorption properties of RE ions toward radionuclides, the conjugation of radionuclides onto the surface of UCNPs offers a promising approach. In 2020, Li *et al.* developed FA-modified, red blood cell membrane-coated UCNPs with a pretargeting strategy for TNBC MRI/UCL/PET imaging (Figure 10C). They prepared red blood cell membrane vesicles, fused them with UCNPs, and modified the complex with 1,2-distearoyl-sn-glycero-3-phosphoethanolamine-N-[FA(PEG)-2000]. In 4T1 tumor-bearing mice, these NPs achieved efficient tumor accumulation through FA-mediated targeting and red blood cell membrane-enabled immune evasion, showing peak UCL signals at 36 h post-injection that persisted for 48 h. The Gd^3+^-containing core significantly enhanced tumor MRI contrast versus controls. For PET imaging, mice received pre-injections of 1,2-distearoyl-sn-glycero-3-phosphoethanolamine-N-[azido(PEG)-2000]-modified NPs followed by ^18^F-labeled Al^18^F-NETA-L-DBCO for *in vivo* click chemistry. The FA-modified group exhibited peak tumor radiotracer uptake at 0.5 h with minimal radiation exposure in non-targeted organs 208. Similarly, Fang *et al.* fabricated tumor cell membrane-decorated Gd^3+^-doped upconversion nanoprobes, which successfully differentiated MDA-MB-231 and MCF-7 tumor models *in vivo* through UCL/MRI/PET imaging 235.

### 7.3. Lymph node visualization

Metastasis stands as the predominant cause of therapeutic failure and mortality in BC. As lymph nodes typically represent the first site of metastasis, accurate assessment of their status and treatment of metastatic lymph nodes are of utmost importance. Current guidelines recommend dual-tracer approaches combining radionuclides with blue dyes for lymph node identification. However, this approach confronts substantial limitations, including restricted availability of radioisotopes, stringent regulatory requirements for radiopharmaceutical disposal, inherent instability of short-lived isotopes, and risks of allergic reactions or persistent cutaneous pigmentation associated with dye-based tracers 236.

**Figure 11:**
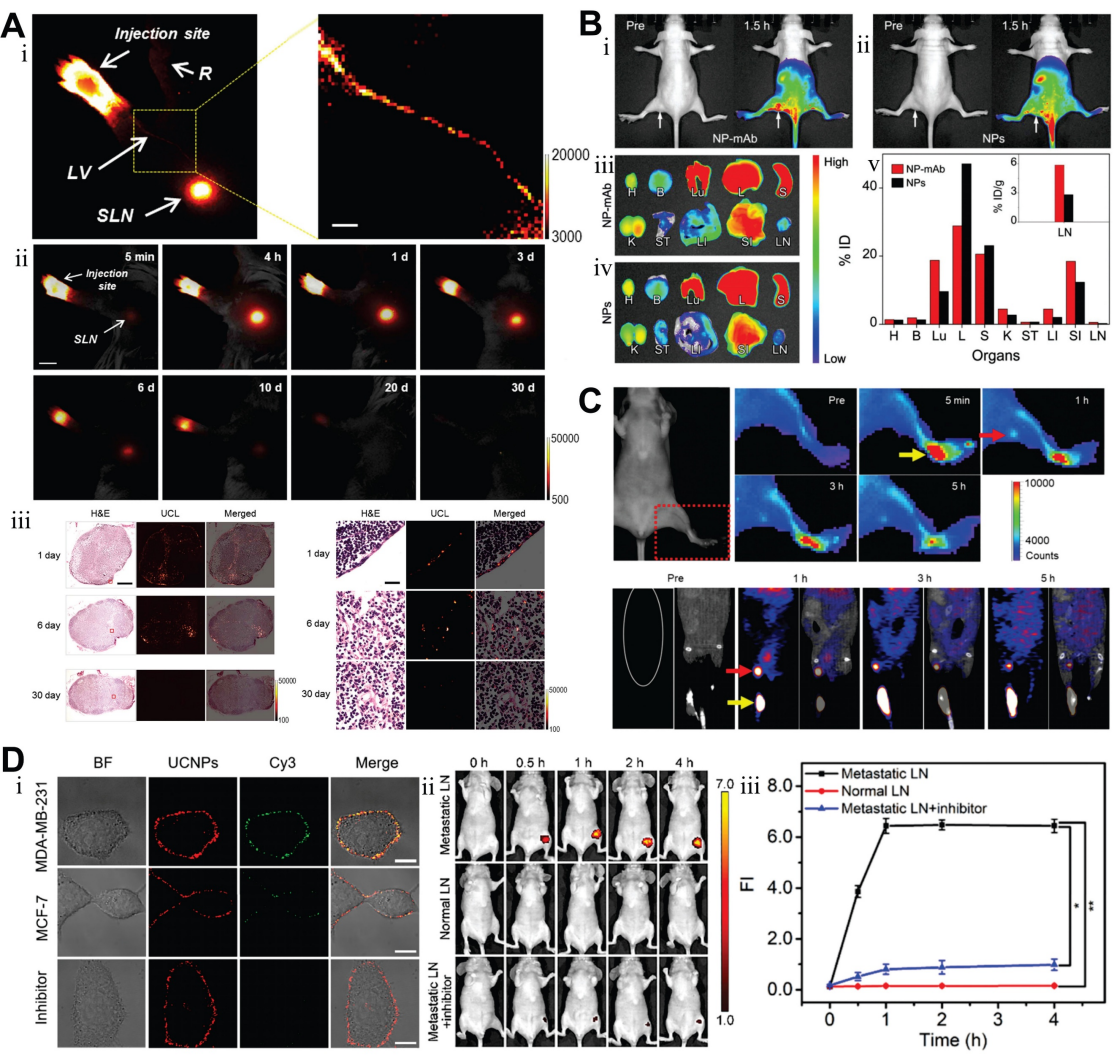
(A) Dynamic distribution of UCNPs in mice after footpad injection. (i) *In vivo* UCL image of mice after injection of the surface-modified UCNPs. LV, lymphatic vessel; SLN, sentinel lymph node; R, reflected secondary; (ii) *In vivo* UCL imaging of lymph node in the same mouse at different post-injection times. Mice were subcutaneously injected in the forepaw footpad with the UCNPs; (iii) H&E staining and corresponding UCL images of dissected axillary lymph nodes from the mice treated with UCNPs. The lymph nodes were obtained on 1, 6, and 30 days after the injections. Adapted with permission from 237, copyright 2016, under a Creative Commons CC BY license. (B) UCL images captured before and at 1.5 h after intravenously delivering the nanoprobes (i) and mother NPs (ii), respectively, into nude mice bearing metastatic lymph nodes as indicated by the white arrows; *ex vivo* UCL images of the main organs and lymph nodes captured right after acquiring the above images (iii, iv) (H, heart; B, brain; L, liver; S, spleen; LU, lung; K, kidney; ST, stomach; LI, large intestine; SI, small intestine; LN, lymph node), together with the quantified biodistribution profile (vi). Adapted with permission from 238, copyright 2018, Royal Society of Chemistry. (C) Fluorescence (top) and PET imaging (bottom) of metastatic lymph nodes (red arrow: lymphatic metastasis, yellow arrow: injection site in foot pad). Adapted with permission from 201, copyright 2022, under a Creative Commons CC BY license. (D) *In situ* protease secretion visualization and metastatic lymph nodes imaging via a cell membrane-anchored upconversion nanoprobe. (i) Confocal microscopy images of upconversion nanoprobe-anchored MDA-MB-231 cells, MCF-7 cells, and inhibitor pre-treated MDA-MB-231 cells upon lipopolysaccharide activation; (ii) Fluorescence images and (iii) average fluorescence intensities of Cyanine 3 at 580 nm from metastatic lymph node, normal lymph node, and metastatic lymph node with the inhibitor in living mice before (0 h) and 0.5, 1, 2, and 4 h post NPs injection. Adapted with permission from 240, copyright 2021, American Chemical Society.

In recent years, engineered UCNPs have emerged as a breakthrough solution for precise localisation of lymph nodes and identification of metastasis. As illustrated in Figure 11A, following subcutaneous administration, engineered UCNPs exhibit superior lymphatic system penetration and selective accumulation in draining lymph nodes, coupled with real-time imaging capabilities 237. On this basis, Qiu *et al.* developed a probe for detecting lymph node metastasis in BC, which employed anti-HER2 antibody-modified NaGdF_4_:Yb,Tm,Ca@NaLuF_4_ NPs (Figure 11B). The results showed that the characteristic emission of the nanoprobes at 804 nm achieved a penetration depth of 7.7 mm through mouse skin tissues, significantly greater than the emissions at 655 nm and 541 nm from commonly used Er-doped UCNPs, which endowed higher sensitivity for diagnosis. Cell binding assays demonstrated that the covalently attached anti-HER2 antibodies conferred excellent binding specificity to HER2-positive cancer cells *in vitro*, thereby enabling the *in vivo* detection of BC lymph node metastasis. Pharmacokinetic studies revealed that antibody conjugation prolonged the blood half-life of UCNPs, reduced liver and spleen uptake, and accelerated biliary excretion 238. In another study, Zhu et al. developed an NIR-IIb fluorescent probe to enhance imaging depth. Following subcutaneous injection, the probe drained to sentinel lymph nodes via the lymphatic system and entered BC cells through C-X-C motif chemokine receptor 4-mediated endocytosis. Under 808 nm laser excitation, the probe emitted NIR fluorescence at 1,556 nm, with a penetration depth of 7 mm. It showed significantly stronger fluorescence signals in metastatic sentinel lymph nodes. In mouse and human BC xenograft models, this probe achieved a sensitivity of over 92% and a specificity of 96% in detecting sentinel lymph node metastasis 239.

Besides, Zhang *et al.* designed UCNPs capable of chelating radionuclides, which were applied not only for lymph node imaging but also for targeted radionuclide therapy of BC lymph node metastasis (Figure 11C). Animal experiments demonstrated that this therapeutic approach significantly reduced the incidence of lymph node metastasis and the volume of metastatic foci. Notably, it showed no significant abnormalities in hematological, hepatic, and renal functions in mice, thus providing a safe and effective novel strategy for the diagnosis and treatment of BC lymphatic metastasis 201. Meanwhile, Fang *et al.* developed a cell membrane-anchored ratiometric UCNPs for monitoring matrix metalloproteinase secretion and imaging metastatic lymph nodes (Figure 11D). The nanoprobe was constructed with UCNPs modified with Cyanine 3, a monitoring matrix metalloproteinase substrate peptide, and an anti-epidermal growth factor receptor antibody. *In vivo* imaging of metastatic lymph nodes in MDA-MB-231 tumor-bearing mice revealed pronounced fluorescence signals in metastatic lymph nodes, validating the specificity and reliability of the nanoprobe 240. Additionally, Li *et al.* developed a liposome-coated UCNPs that enabled precise intraoperative localization of sentinel lymph nodes with an exceptional signal-to-background ratio. Notably, over 90% of the NPs were eliminated via the hepatobiliary pathway within 72 hours post-injection, effectively mitigating potential long-term toxicity 241.

## 8. UCNPs for Detection BC Biomarkers

The development and progression of BC involve a multilevel cascade of abnormalities across molecular, cellular, and histological levels. Unlike overt morphological alterations in advanced stages, early-stage lesions frequently exhibit abnormalities at the biomolecular level. Implementing strategies to detect these tumor biomarkers can improve the early diagnosis rate and enhance the accuracy of subsequent treatment by leveraging detailed molecular-level information. Conventional techniques such as immunohistochemistry (IHC), western blotting (WB), and enzyme-linked immunosorbent assay (ELISA) have limitations in sensitivity, operational complexity, and cost-effectiveness. UCNP-based detection technology has emerged as a promising tool for BC biomarker analysis, leveraging its NIR excitation interference resistance and capacity for multiplexed narrow-band emission. This framework mainly includes two strategic paradigms 242,243. One is homogeneous assays that rely on energy transfer mechanisms such as Förster resonance energy transfer (FRET), realizing rapid "mix-and-read" detection through the modulation of donor-acceptor distance. The other is heterogeneous assays that use high-specificity solid-phase recognition interfaces, including microarray platforms and lateral flow chromatography. This section focuses on UCNP-based detection systems for BC biomarkers, classified according to the type of biomarker being targeted (Table 5).

### 8.1. Protein

ER, PR, and HER2 serve as critical biomarkers for molecular subtyping and therapeutic decision-making in BC 244. In 2020, Farka *et al.* engineered UCNP-based nanoconjugates for HER2 quantification. When applied to HER2-positive BT-474 cells, these conjugates achieved a 50-fold enhancement in the signal-to-background ratio compared to conventional fluorescent labeling. Additionally, UCNP labeling demonstrated compatibility with H&E staining, neither interfering with signal acquisition nor compromising diagnostic accuracy 245. Similarly, Gorris *et al.* developed streptavidin-PEG-UCNP conjugates modified with alkyne-PEG-neridronate, which enabled specific binding to HER2 via biotinylated anti-HER2 antibodies. The optimized conjugates exhibited an unprecedented signal-to-background ratio of 300 or more in IHC, thereby allowing high-contrast imaging of HER2 overexpression on BC cell membranes without optical background interference 246.

**Figure 12:**
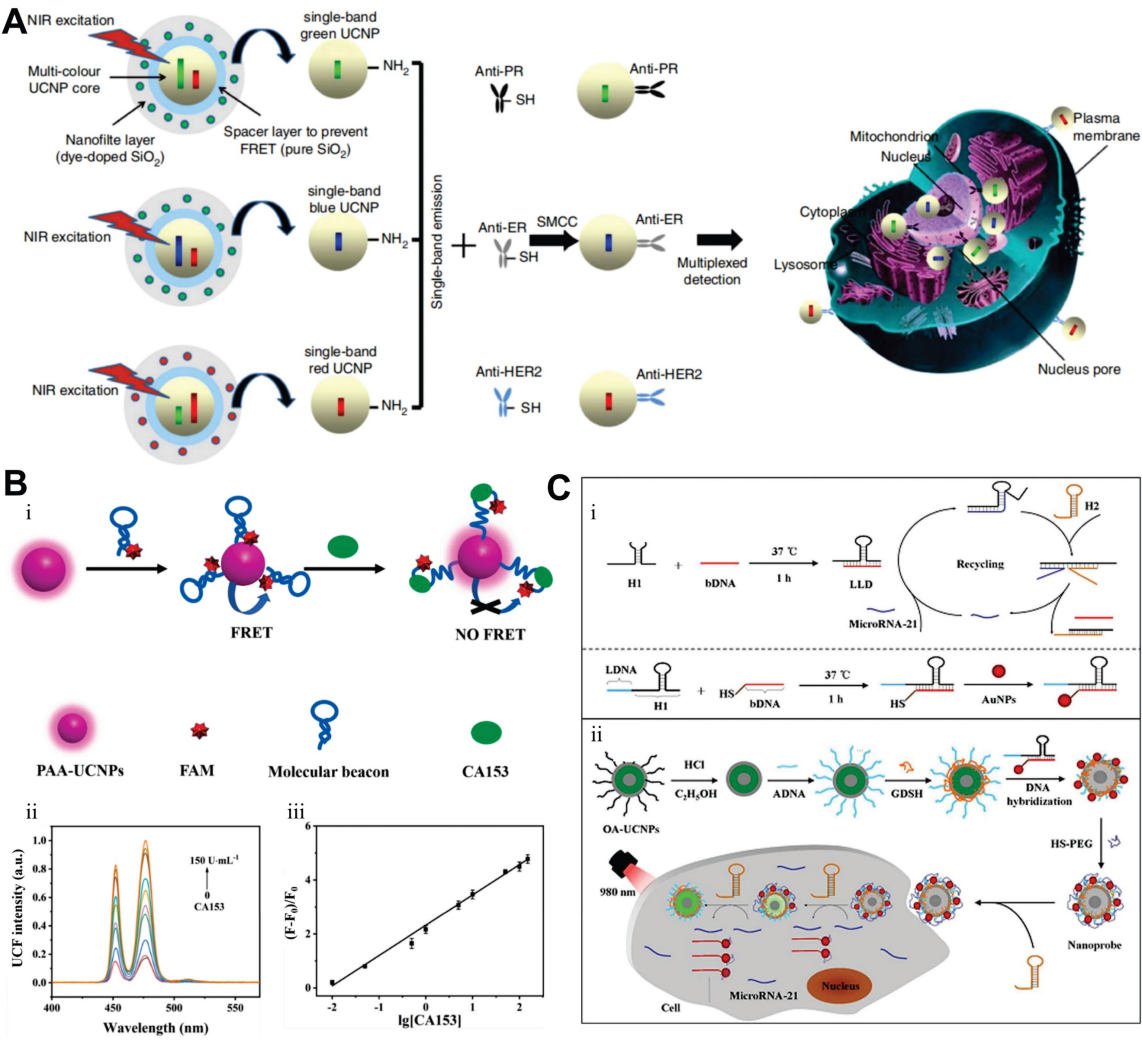
(A) Surface amino modifications of green, blue, and red single-band UCNPs and antibody conjugates for multiplexed *in situ* molecular mapping of BC biomarkers PR, ER, and HER2. Adapted with permission from 247, copyright 2015, under a Creative Commons CC BY license. (B) (i) Working principle of CA153 biosensor based on FRET; (ii) UCL spectrum and (iii) the corresponding UCL intensity at 475 nm of the nanoprobe after reaction with various concentrations of CA153 in 100-fold diluted human serum. Adapted with permission from 253, copyright 2022, under a Creative Commons CC BY license. (C) Schematic illustration of the preparation of lock-like DNA (top) and upconversion nanoprobe (bottom), and application in monitoring microRNA-21 in living cells. Adapted with permission from 269, copyright 2018, WILEY-VCH Verlag GmbH & Co. KGaA, Weinheim.

Notebly, the identification of potential diagnostic markers from a plethora of oncoproteins necessitates technologies for quantitative analysis of multiple biomarkers. For *in situ* multiplexed molecular mapping, accurate profiling can only be achieved by using single-band emission nanoprobes to eliminate crosstalk between labeling signals. In response to this need, Zhou *et al.* developed nanoprobes capable of emitting blue, green, and red single-band light, which were used to enable simultaneous *in situ* molecular imaging and quantitative detection of ER, PR, and HER2 in BC cells and tissues (Figure 12A). Multispectral confocal microscopy revealed precise subcellular localization of the probes, with ER and PR distinctly labeled in the nucleus and HER2 on the membrane of both MCF-7 and MDA-MB-231 cells. Quantitative analysis revealed ER/PR/HER2 expression levels of 76%/79%/65% in MCF-7 cells versus 5%/11%/4% in MDA-MB-231 cells, aligning with WB. In clinical BC tissues, nanoprobe quantification strongly correlated with IHC for high-abundance biomarkers. Notably, the combined nanoprobe-spectroscopic analysis improved the accuracy of detecting low-expression targets compared with IHC alone, demonstrating potential for resolving ambiguous biomarker expression cases 247.

As a key driver of BC progression, tumor-secreted vascular endothelial growth factor (VEGF) not only stimulates angiogenesis but also directly promotes tumor expansion, rendering its detection of critical clinical significance. As nucleic acid molecules, aptamers offer distinct advantages over traditional antibodies, including high chemical stability, easy modifiability, strong specificity, small molecular weight, and non-immunogenicity, and enable the functionalization of UCNPs via a simple one-step exchange strategy. In 2016, Lan *et al.* synthesized NaYF_4_:Yb,Er NPs, replacing the surface oleic acid with aptamer DNA to construct a microplate-based detection system. The capture probe immobilized VEGF, which then bound to the UCNP-labeled auxiliary probe. This method exhibited a linear relationship between UCL intensity at 541 nm and VEGF concentration in the range of 50-2000 pM, with a limit of detection (LOD) of 6 pM. In spiked serum samples, the recovery of 500 pM VEGF ranged from 98% to 113%, with relative standard deviations of 2.9%-3.6%. The high specificity of aptamers for VEGF effectively eliminated interference from serum albumin in complex serum matrices 248. In contrast to the microplate-based detection system, the sensitive aptasensor based on FRET employs a homogeneous assay strategy that eliminates the need for separation steps, offering improved operational simplicity and sensitivity. For instance, Yuan *et al.* developed a highly sensitive FRET-based aptasensor for VEGF_165_ detection, using PAA-modified UCNPs as energy donors and MoS_2_ nanosheets as acceptors. FRET triggered by aptamer-MoS_2_ physical adsorption quenched UCNP fluorescence by 95%. Upon VEGF_165_ addition, aptamer binding induced conformational changes, weakening van der Waals forces with MoS_2_ to separate donor-acceptor pairs, inhibiting FRET and restoring fluorescence. The linear fluorescence recovery range for VEGF_165_ was 0.1-16 ng/mL with a 0.1 ng/mL detection limit 249.

### 8.2. Glycoprotein

Serum tumor markers serve as indispensable indicators for cancer screening, monitoring recurrence, and guiding therapeutic strategies. Cancer antigen 15-3 (CA15-3), a transmembrane glycoprotein from the MUC-1 family detected by monoclonal antibodies DF3 and 115D8, is widely expressed in breast, lung, ovarian, and pancreatic malignancies. Since the 1980s, serum CA153 levels have been used as a diagnostic and assessment indicator for BC 250-252. In 2022, Liang *et al.* developed a homogeneous biosensor for the detection of CA15-3 in human serum based on the FRET strategy (Figure 12B). The biosensor uses NIR-excitable UCNPs as the energy donor and the commercial organic dye as the energy acceptor, which are linked by a molecular beacon containing the CA153 aptamer sequence. The upconversion fluorescence of UCNPs can be effectively quenched by dye. In the presence of CA15-3, the hairpin structure of molecular beacon is opened, leading to the separation of UCNPs and dye and the inhibition of FRET, so the fluorescence is recovered. In HEPES buffer, the fluorescence has a linear relationship with the logarithm of CA15-3 concentration in the range of 0.01-150 U/mL, with an LOD of 4.5 mU/mL. The probe has good selectivity and can be successfully applied to the detection of CA15-3 in human serum, providing a useful tool for the early diagnosis of BC 253.

Similarly, Cancer antigen 125 (CA125), another key biomarker derived from the MUC1 gene, plays a central role in regulating multicellular survival pathways within BC cells 254. In response, researchers developed an upconversion fluorescence biosensor for CA125 detection by constructing a "sandwich" structure using surface-modified UCNPs and silver NPs conjugated with CA125 antibodies and antigens, achieving fluorescence quenching with a linear detection range of 5-100 ng/mL 255. Meanwhile, Zhang *et al.* utilized carbon dots as energy acceptors, combined with aptamer-modified UCNPs through π-π stacking interactions to quench UCL. In the presence of CA125, the UCL was restored, enabling an LOD as low as 9.0 × 10^-3^ U·mL^-1^ and demonstrating excellent performance in human serum 256. Recently, Ekman *et al.* developed a spectrally separated dual-label UCL lateral flow assay to simultaneously detect cancer-specific STn-glycosylated forms of CA125 and CA15-3. Using Er^3+^-doped (540 nm emission) and Tm^3+^-doped (450 nm emission) UCNPs conjugated with anti-CA125/CA15-3 antibodies, the assay employed a single test line immobilized with anti-STn antibody to capture target glycoproteins. The dual-label lateral flow assay discriminated ascites samples from cancer patients and liver cirrhosis controls, with LOD for CA125-STn at 1.8 U/ml in buffer and 3.6 U/ml in ascites, and a linear range of 1-2500 U/ml 257.

Carcinoembryonic antigen (CEA), the first tumor antigen studied as a glycoprotein molecule associated with cell adhesion, exhibits elevated serum levels that may indicate the presence of malignant tumors in endodermal tissues, including the breast 258. Given its clinical significance, extensive research has focused on improving detection sensitivity and specificity, leading to the design of a series of innovative detection systems based on UCNPs. Among these, the most widely studied paradigm utilizes UCNPs as energy donors in combination with nanomaterials as energy acceptors, enabling quantitative detection of CEA through fluorescence signal quenching or recovery 259-261. For instance, Yu *et al.* proposed a modification-free fluorescent biosensor based on polydopamine (PDA)-coated UCNPs for the detection of CEA. The biosensor consisted of UCNPs@PDA and CEA aptamer-functionalized gold nanoparticles (AuNPs). Due to the π-π stacking and hydrogen bonding interactions, the CEA aptamer on AuNPs was adsorbed onto the surface of UCNPs@PDA, triggering the FRET from UCNPs@PDA to AuNPs-CEA aptamer and leading to the quenching of fluorescence. In the presence of CEA, the AuNPs-CEA aptamer detached from UCNPs@PDA because of the stronger affinity between CEA and its aptamer, blocking the FRET process and thus recovering the fluorescence, which enabled the quantitative analysis of CEA concentration 262.

Additionally, addressing the limitations of traditional optical intensity-based detection, the composite signal conversion paradigm based on UCNPs has emerged. For instance, to enhance detection sensitivity, Qiu *et al.* developed a photoelectrochemical aptasensing platform using core-shell NaYF_4_:Yb,Tm@TiO_2_ upconversion microrods, leveraging NIR to UV light conversion to activate TiO_2_-mediated photocurrent generation. The platform integrated target-triggered rolling circle amplification, where CEA binding initiated this process to generate guanine-rich strands digested by exonucleases, releasing free guanines that enhanced photocurrent. This design exhibited a linear range of 10 pg/mL to 40 ng/mL for CEA, with an LOD of 3.6 pg/mL, high selectivity against interferents, and reproducible responses under repeated NIR illumination. Validation in human serum samples showed strong agreement with commercial ELISA, confirming its clinical utility 263. To address the clinical need for wide dynamic range detection, Shao *et al.* constructed a trimodal sensing platform using three-layer dumbbell-like UCNPs combined with G-quadruplex DNAzyme. By tuning Nd^3+^ doping, the platform integrated UCL, photothermal, and colorimetric signals. The UCL mode achieved a linear range of 0.005-50 ng/mL, the photothermal mode covered 50-2000 ng/mL, and the colorimetric mode spanned 10-1000 ng/mL, providing a comprehensive detection range of 0.005-2000 ng/mL 264. In another study, Xu *et al.* developed an aptamer-based biosensor using encoded UCNPs for digital detection. The system formed a "magnetic bead-aptamer-UCNPs" sandwich structure, where CEA binding dissociated complementary DNA-UCNPs from magnetic beads due to stronger aptamer-CEA affinity. The released UCNPs were counted via fluorescence microscopy, establishing a linear relationship with CEA concentration. This label-free counting strategy avoided energy transfer dependencies and background interference, offering a novel path for ultrasensitive detection in complex matrices 265.

### 8.3. Nucleic acids

Nucleic acids, serving as the carriers of genetic information storage and transmission, exhibit dysregulation closely linked to the progression of cancer. The detection of nucleic acids using UCNPs stands out for its unique integration of nanoscale assembly strategies and signal transduction mechanisms, enabling unprecedented *in situ* quantification. For instance, thymidine kinase 1, a key enzyme in DNA synthesis and cellular proliferation, has been investigated as a prognostic marker and early indicator of treatment response in HER2-negative early and metastatic BC. Gao and colleagues developed spiny nanorod-UCNP satellite assemblies for ultrasensitive detection of thymidine kinase 1 messenger RNA (mRNA) in living cells. The design relied on target recognition to dissociate UCNPs from the matrix, thereby restoring UCL. This strategy achieved an LOD of 0.67 fmol/10 μg RNA and successfully quantified mRNA levels in MCF-7 and HeLa cells, demonstrating its utility for intracellular nucleic acid analysis 266. Furthermore, to integrate detection and therapy, a theranostic nanobeacon was constructed, combining a thymidine kinase 1 mRNA-specific molecular beacon with UCNPs and loading the chemotherapeutic drug. The nanobeacon enabled ratiometric UCL detection of thymidine kinase 1 mRNA with an LOD of 1.1 nM, while target binding triggered drug release for chemotherapy 267.

Moreover, Li *et al.* constructed DNA-driven chiroplasmonic nanopyramids by self-assembling AuNPs and UCNPs, creating a dual-mode sensing platform that combined plasmonic circular dichroism and UCL for microRNA-21 detection in MCF-7 cells. The nanopyramids displayed a circular dichroism signal at 521 nm and UCL in the 500-600 nm range, with circular dichroism intensity decreasing and UCL increasing upon microRNA-21 binding, achieving an LOD of 0.03 fmol/10 μg RNA due to plasmonic enhancement of DNA chirality, which enabled precise quantification of microRNA-21 in MCF-7 cells and distinguished them from normal uterine fibroblast cells based on expression levels 268. In another study, Zhang *et al.* advanced this paradigm with lock-like DNA-programmed UCNPs-AuNPs assemblies, where a hairpin DNA hybridized with a bolt DNA to form lock-like DNA, tethering AuNPs to UCNPs and quenching UCL via FRET (Figure 12C). When microRNA-21 was present, fuel hairpin DNA triggered cyclic disassembly. This allowed a single microRNA-21 molecule to repeatedly unlock multiple lock-like DNA units and dissociate AuNPs from UCNPs, restoring UCL with nonenzymatic signal amplification. This strategy achieved an ultra-low LOD of 0.74×10^-15^ M for microRNA-21, approximately 1000 times more sensitive than non-amplified probes, and clearly distinguished MCF-7 cells from normal L-02 cells by imaging intracellular microRNA-21 levels, leveraging target cycling to generate high signal gain suitable for detecting low-abundance microRNAs in BC cell 269.

Meanwhile, circulating tumor DNA (ctDNA) has emerged as a critical biomarker for BC management, owing to its short half-life and ability to reflect disease status in real time 270. However, ctDNA detection faces challenges such as ultra-low abundance, fragment brevity, and rapid degradation, while conventional sequencing methods often fail due to background noise masking tumor-derived signals. Wang *et al.* designed a satellite assembly nanoprobe combining UCNPs and gold nanocages, adjusting the excitation and emission wavelengths of UCNPs to the NIR region via doping with Yb and Tm ions. By constructing an FRET system through complementary DNA pairing between UCNPs and gold nanocages with corresponding wavelength absorption, and using toehold-mediated strand displacement reaction for signal transduction, this approach achieved sensitive detection of KRAS gene point mutations with an LOD of 6.30 pM 271. Gong *et al.* further developed an NIR light-responsive ctDNA capture-release platform. By using UCNPs to convert NIR light into UV light, the platform triggered the trans-cis isomerization of azo units, enabling the reversible release of ctDNA-probe complexes. This allowed the detection chip to be reused, providing new ideas for quantitative analysis of ctDNA and personalized diagnosis of cancer 272.

## 9. UCNPs for BC Phototherapy

Phototherapy boasts a history of thousands of years, with ancient records in India, China, and Egypt document the use of sunlight for treating skin diseases 273. The invention of the laser in 1960 revolutionized phototherapy, enabling applications in ophthalmic surgery and tumor ablation 274. While laser-based phototherapy exhibits less systemic toxicity than traditional chemotherapy due to its localized action, it still harbors critical limitations. Endogenous chromophores in non-malignant tissues cause off-target effects, compromising selectivity for malignant cells. Additionally, the requirement for high power density in laser therapy raises safety concerns and logistical challenges. In response, photodynamic therapy (PDT) using exogenous photosensitizers (PS) and photothermal therapy (PTT) employing photothermal agents (PTA) have emerged to provide more targeted and effective therapeutic options 275. However, current PDT and PTT face significant drawbacks. For instance, most PS are constrained by their absorption primarily in the UV/visible spectra, leading to strong tissue absorption/scattering and shallow penetration. Some PTA suffer from insufficient biocompatibility and low photothermal conversion efficiency. Furthermore, the lack of imaging guidance and real-time efficacy monitoring during treatment often causes unintended damage to normal tissues 276. Against this backdrop, composite systems integrating UCNPs with PS or PTA have emerged as promising solutions. These systems not only overcome the limitation of treatment depth by leveraging NIR light-induced short-wavelength emission from UCNPs but also enhance photothermal conversion efficiency. More importantly, the excellent imaging capabilities of UCNPs enable real-time therapeutic guidance, significantly improving treatment precision and safety. Herein, we deliver an in-depth exploration of UCNP-based phototherapy for BC (Table 6).

### 9.1. Photodynamic therapy

**Figure 13:**
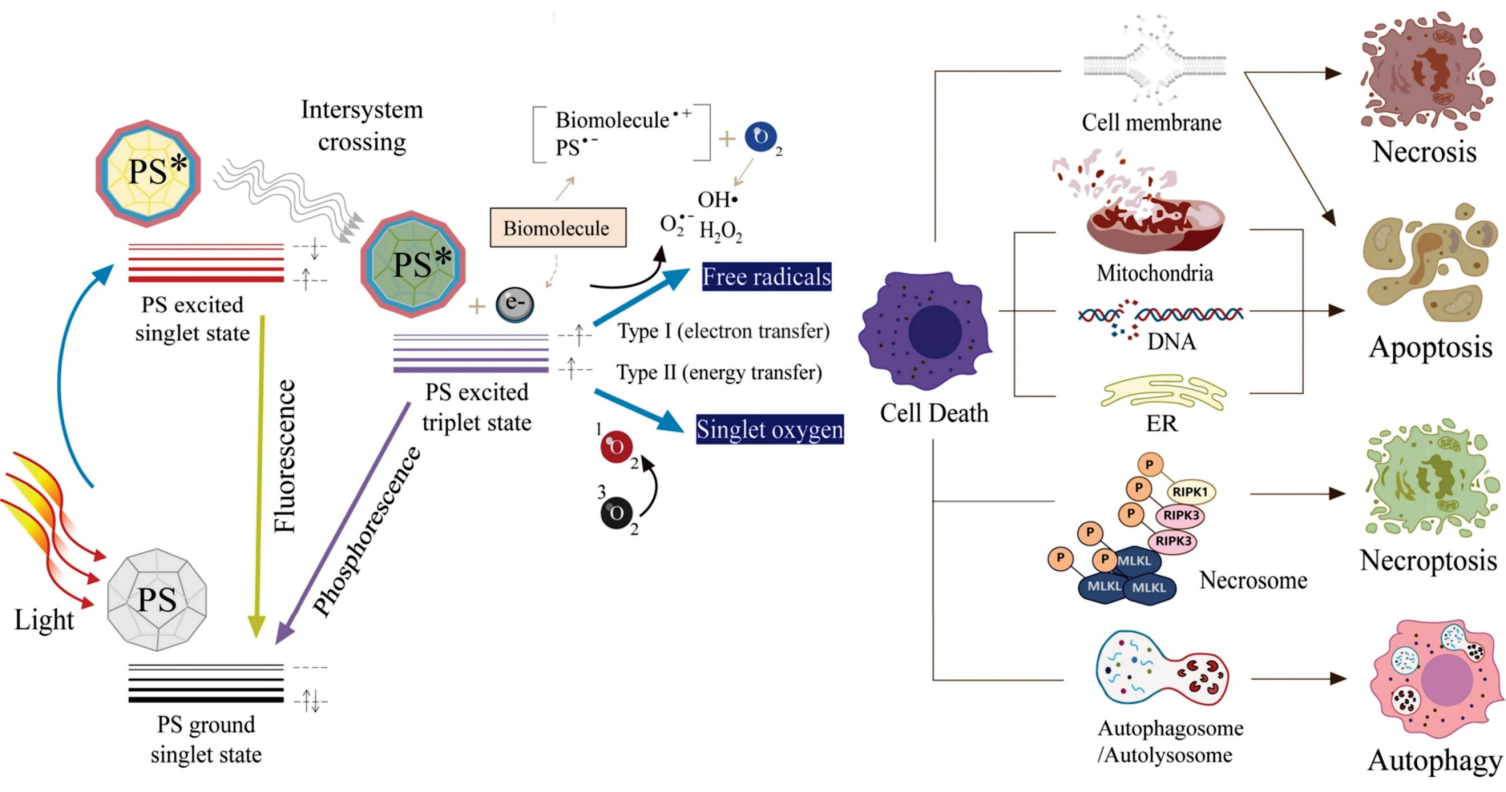
Mechanism of photodynamic reactions (either type I or type II) and cell death pathways in the process of PDT. Adapted with permission from 279, copyright 2021, under a Creative Commons CC BY license.

PDT has been a clinically proven method for over 40 years in the treatment of superficial skin lesions, esophagus, lung and bladder tumors 277,278. It is based on three essential elements: oxygen, PS, and specific radiation. As illustrated in Figure 13, the resulting photodynamic response diverges into two distinct mechanisms. Type I reactions entail direct hydrogen or electron transfer, leading to the generation of free radicals that induce oxidative stress and ultimately result in cell death. Type II, more prevalent in PDT, entails energy transfer to oxygen to form highly reactive singlet oxygen (^1^O_2_), which damages nearby biomolecules and leads to cell death 279. As the key component of PDT, PS have optical properties that directly affect treatment efficacy. Conventional PS, including first-generation porphyrins, second-generation chlorophyll derivatives, and third-generation phthalocyanines, mainly absorb light in the 400-700 nm range. However, biological tissues exhibit strong scattering and absorption in this spectral band, which severely restricts the efficacy of PDT.

Novel UCNP-based PDT systems use engineered UCNPs as converters, efficiently absorbing high-penetration NIR light with minimal damage, and emitting UV/visible light to activate PS, thus enhancing PDT efficacy. For example, Khaydukov *et al.* developed UCNPs converting 975 nm light to UV-blue light for riboflavin activation in BC treatment. With 2% UV-blue and 9.5% total conversion efficiency, these UCNPs showed significant cytotoxicity in SK-BR-3 cells under 975 nm light *in vitro*. In immunodeficient mice, injecting UCNP-riboflavin mixtures around tumors, then NIR irradiation, inhibited tumor growth by 90% in 50 days. This approach extended the treatment depth to 4-6 mm, tenfold deeper than conventional UV-blue light 280. Besides, Yong Zhang and colleagues developed a silk-fibroin-coated implant by incorporating submicrometer UCNPs into polydimethylsiloxane for remote orthotopic PDT. *In vitro*, this implant induced approximately 48% ROS in MCF7 cells and 37% in MDA-MB-231 cells, representing a fivefold increase over conventional implants. It also promoted significant apoptosis. In orthotopic breast cancer mouse models, the implant reduced tumor burden by 60% compared to controls. The system achieved 1.0 cm tissue penetration depth under NIR excitation, and its clinical prototype integrated both cosmetic and therapeutic functions, demonstrating potential for deep tumor therapy 281. In another study, bio-nanohybrids of KillerRed covalently conjugated to UCNPs were efficiently internalized by MDA-MB-231 cells and distributed in the cytoplasm. Under 980 nm NIR excitation, UCNPs converted NIR light to green light, effectively activating KillerRed to generate ROS. This achieved approximately 70% PDT efficiency through 1 cm-thick tissue, whereas the efficiency of conventional KillerRed dropped to only 7% under the same condition 282.

Building on targeted approaches, Ramírez-García *et al.* designed an immunoconjugated upconversion nanocomplex specifically for HER2-positive BC imaging and PDT. They synthesized NaYF_4_:Yb,Er UCNPs via thermal decomposition, modified them with cysteamine, covalently linked zinc phthalocyanine (ZnPc), and conjugated the HER2-targeting antibody trastuzumab. This complex efficiently converted 975 nm light into 659 nm red emission from the UCNPs, subsequently triggering ZnPc to generate ^1^O_2_ with a high energy transfer efficiency of 84.3%. Confocal microscopy confirmed selective binding to HER2-positive SKBR-3 cells with minimal attachment to HER2-negative MCF-7 cells. Under NIR irradiation for just 5 minutes, the nanocomplex at 200 μg/mL reduced SKBR-3 cell viability to 21%, compared to 93.5% viability without light 216. Zhang *et al.* proposed in 2017 a strategy integrating UCNPs with graphene quantum dots for mitochondria-specific PDT. Under NIR excitation, the UCNPs emitted UV-visible light that activated the graphene quantum dots to produce ^1^O_2_. Modification with tetramethylrhodamine isothiocyanate endowed the platform with mitochondrial targeting capability, enabling in-situ ^1^O_2_ generation within mitochondria. This process significantly decreased mitochondrial membrane potential, activated caspase 3, and triggered tumor cell apoptosis. *In vitro* experiments showed significantly higher cytotoxicity against 4T1 cells under light irradiation compared to non-targeted controls, while maintaining biocompatibility in the dark. *In vivo* studies demonstrated a 75.3% tumor inhibition rate in 4T1 tumor-bearing mice, outperforming the non-targeted system 283. Similarly, Liu *et al.* designed a Nd^3+^-sensitized upconversion composite for NIR light-triggered, mitochondria-targeted PDT. The composite was composed of UCNPs and porphyrinic metal-organic frameworks, which were further surface-functionalized with triphenylphosphine (a mitochondria-targeting ligand), enabling the generation of ^1^O_2_ activated by 808 nm light 284. Additionally, strategies responsive to the TME have been developed, such as pH-sensitive targeting systems that enhance tumor accumulation and PDT efficacy by exploiting the acidic TME 285.

**Figure 14:**
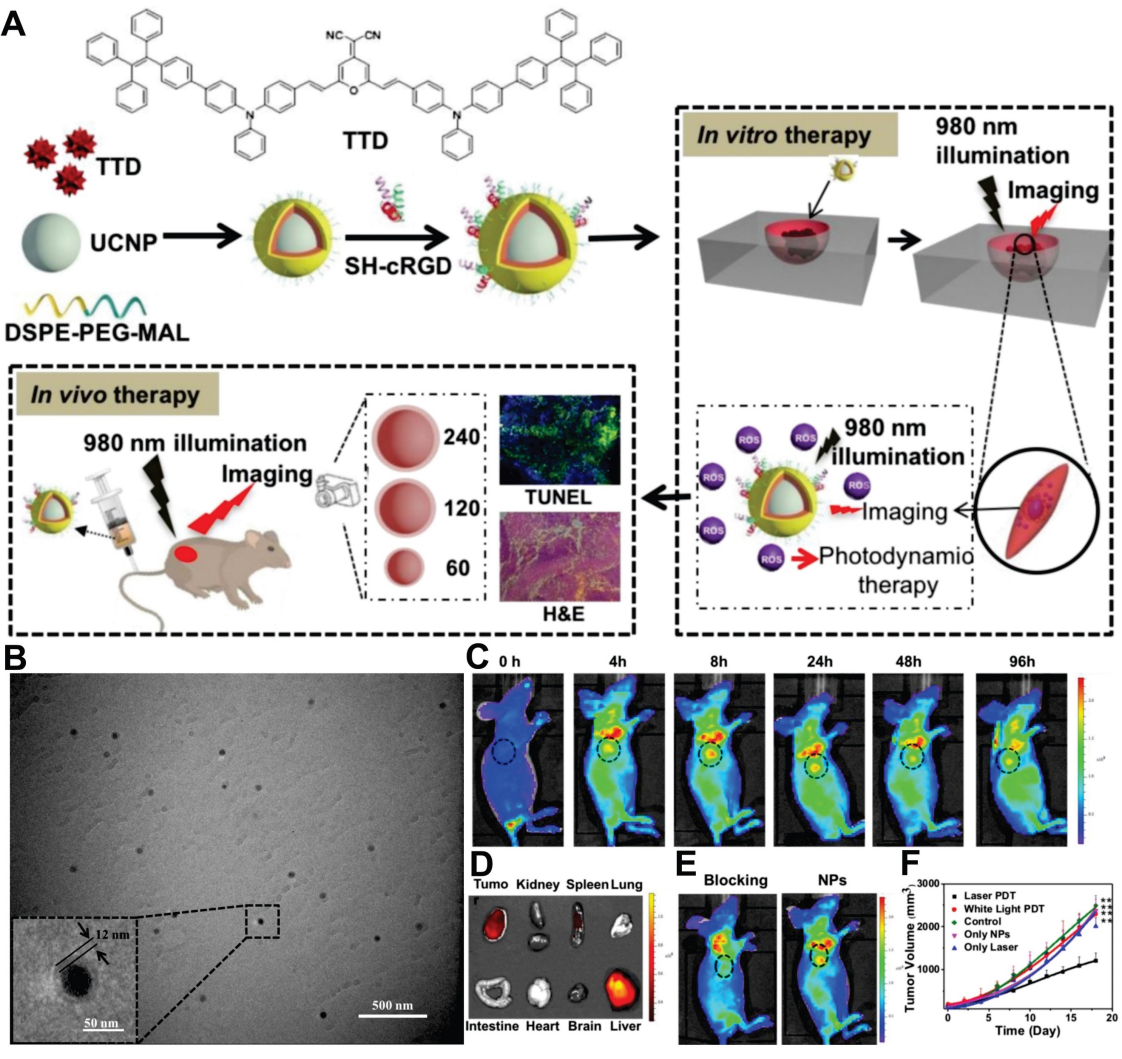
(A) Schematic illustration of preparation of NPs and their applications in bioimaging and PDT of deep-seated tumors upon NIR laser illumination, in an *in vitro* three-dimensional cancer cell spheroid and in a murine tumor model, respectively. (B) The morphology of NPs under transmission electron microscopy. (C) Biodistribution of NPs in tumor-bearing mice after intravenous injection of NPs (30 mg/kg) at different times. Black circles indicate the tumor. (D) *Ex vivo* fluorescence imaging of various organs and tumor tissues from mice intravenously injected with NPs. The mice were sacrificed at 12 h post-injection. (E) Biodistribution of NPs in tumor-bearing mice 8 h after intravenous injection of NPs (30 mg/kg) without or with blocking the receptors. Red circles indicate the tumor. (F) Growth curves of tumors in laser PDT, white light PDT, NP-only, laser-only and control groups, respectively, and NPs were intravenously injected into tumors with an initial tumor volume of 120 mm^3^. Adapted with permission from 286, copyright 2019, under a Creative Commons CC BY license.

Leveraging the inherent imaging capabilities of UCNPs, Jin *et al.* developed an NIR-regulated theranostic nanoplatform in 2019 (Figure 14). They encapsulated UCNPs and an aggregation-induced emission luminogen within an amphiphilic polymer and conjugated cyclic arginine-glycine-aspartic acid peptide for targeting. Excitation at 980 nm yielded a ^1^O_2_ quantum yield of 36.4%, with light penetration up to 6 mm of tissue. The nanocomposite retained 75% of its initial fluorescence intensity after 10 days, outperforming commercial probes. Following intravenous injection, it showed time-dependent tumor accumulation, peaking at 8 hours. *In vitro*, it specifically targeted αvβ3 integrin-overexpressing MDA-MB-231 cells, reducing viability to 28.3% under NIR-mediated PDT. *In vivo*, it achieved a 75% tumor growth inhibition rate in MDA-MB-231 tumor-bearing mice 286. Similarly, Lv's team created degradable peptide-modified NPs for NIR-II imaging and UCL-guided PDT of TNBC. Synthesized via double emulsion, the composite integrated ultra-small UCNPs, polymers, and embedded ZnPc. Under 980 nm excitation, the core-shell structure exhibited enhanced 650 nm red emission with 38.3% energy transfer efficiency and superior NIR-II imaging. *In vitro*, it induced effective PDT and degraded into sub-6 nm particles. *In vivo*, the peptide modified composite selectively targeted MDA-MB-231 tumors, significantly inhibiting growth while maintaining biocompatibility and low toxicity 152. In conclusion, UCNPs appear to be a promising carrier for targeted PDT of BC.

### 9.2. Photothermal therapy

PTT employs PTA to convert light energy into heat, inducing irreversible thermal ablation of tumor cells through localized hyperthermia 287. Its biological effects exhibit distinct temperature-dependent characteristics. At 41 °C, cells initiate a heat-shock response involving altered gene expression and heat-shock protein production to counteract initial damage. At 42 °C, tissues suffer irreversible injury. When exposed to 42-46 °C for 10 min, necrosis occurs, releasing intracellular damage-associated molecular patterns that may trigger inflammation. In the 46-52 °C range, cells rapidly undergo apoptosis (programmed cell death), a process preserving membrane integrity while externalizing "eat-me" signals like phosphatidylserine to facilitate phagocytic clearance, typically without significant inflammation 288,289. Temperatures exceeding 60 °C cause instantaneous protein denaturation and membrane disruption, leading to immediate coagulative necrosis. PTT is categorized as traditional PTT (≥45 °C) or increasingly utilized mild PTT (<45 °C), with the latter often serving as a regulatory mechanism rather than directly targeting tumor destruction 290. However, the temperature regulation dilemma of PTT and the performance limitations of existing PTA have prompted researchers to focus on functional nanomaterials with unique optical properties. Recently, UCNPs have been integrated with conjugated polymers, organic dyes, plasmonic metals, carbon, and inorganic materials for PTT. This approach enhances PTA stability, biocompatibility, and absorption wavelength while enabling synergistic multimodal imaging integration with optimized energy conversion efficiency.

Gold-based nanomaterials including nanoshells, nanorods, nanostars, nanocages, and Au_25_ clusters induce SPR, making them widely used as PTA in PTT. Notably, smaller AuNPs exhibit reduced photothermal conversion efficiency under NIR irradiation due to mismatch between their SPR absorption peaks (500-560 nm for NPs several nanometers in size) and NIR light 276. In this regard, Gold-decorated UCNPs address this challenge. When excited by 980 nm NIR light, the NaYF_4_:Yb,Er@NaYF_4_@SiO_2_ NPs generated green light through upconversion, a process that coupled with the SPR of AuNPs to enable photothermal conversion and thereby endowed the system with efficient photothermal killing effects against BE(2)-C neuroblastoma cells 291. Moreover, to assess AuNPs versus gold nanorods (AuNRs) in absorbing energy from NaYF_4_:Yb,Er, Chen *et al.* synthesized UCNPs@SiO_2_@Au nanocomposites. They systematically investigated energy transfer mechanisms and efficiency from UCNPs to gold nanomaterials by characterizing variations in UCL intensity and photoluminescence lifetime. Results demonstrated UCNPs@SiO_2_@AuNRs exhibit nearly 26-fold higher heat generation efficiency than UCNPs@SiO_2_@AuNPs, attributed to enhanced SPR-UCL interaction in the nanorod-based system 292. In a separate study, Jiang *et al.* engineered several classic inorganic-organic PTT nanocomposites by integrating UCNPs with copper sulfide, manganese dioxide, carbon, dopamine, and polypyrrole. Findings revealed PTT efficacy primarily stems from NIR light absorption, with UCL light conversion providing a secondary contribution 293.

**Figure 15:**
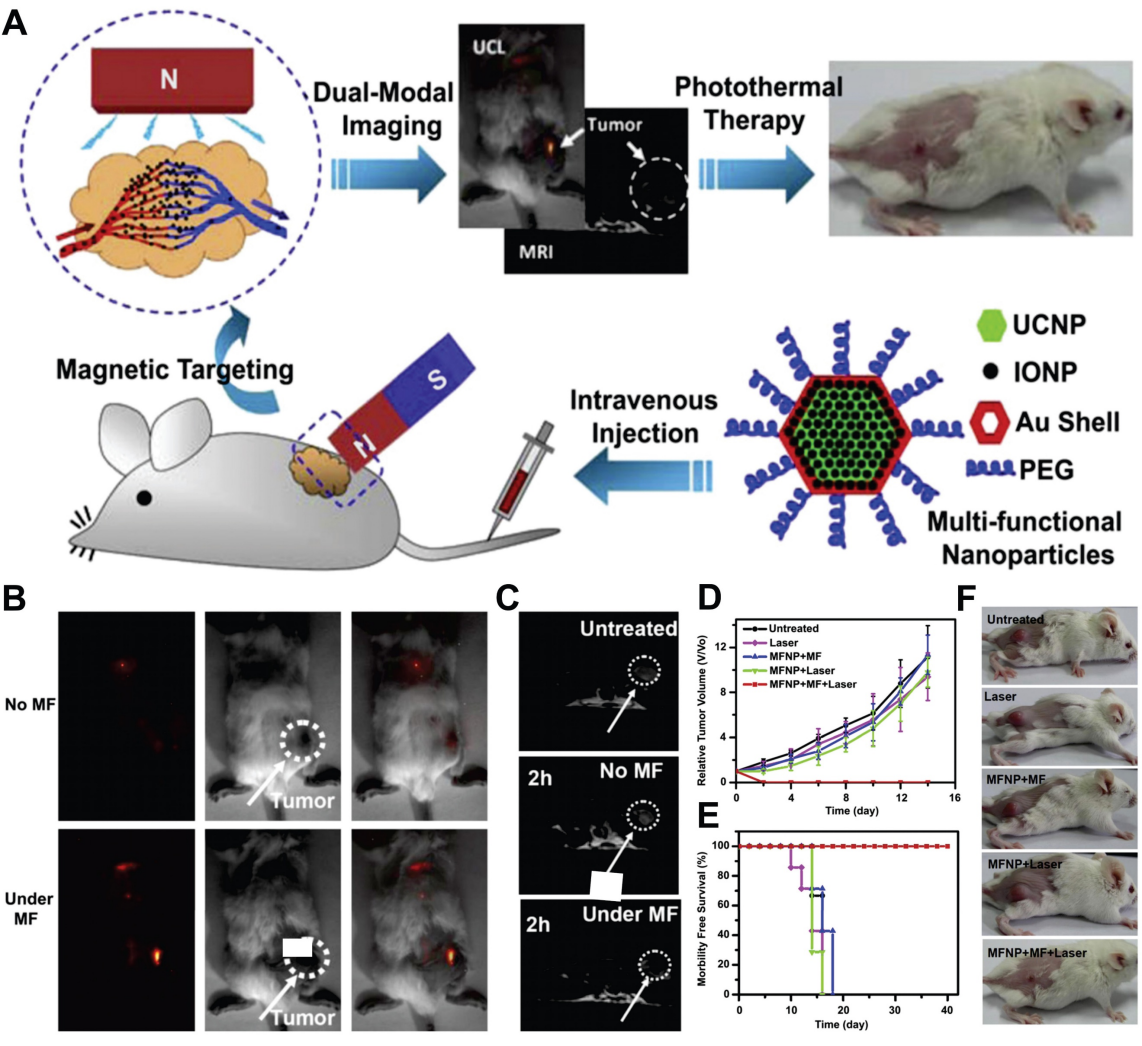
(A) A schematic illustration showing the composition of nanocomposites and the concept of *in vivo* imaging-guided magnetically targeted photothermal therapy. (B) Representative *In vivo* UCL images of 4T1 tumor-bearing Balb/c mice taken 2 h after injection of NPs under the tumor-targeted magnetic filed (top) and without the magnetic field (bottom). (C) Representative *In vivo* T2-weighted MRI images of 4T1 tumor-bearing mice with and without magnetic targeting acquired 2 h after injection of NPs. (D) The growth of 4T1 tumors in different groups of mice after treatment. (E) Survival curves of mice bearing 4T1 tumor after various treatments indicated. (F) Representative photos of mice after various treatments indicated. Adapted with permission from 294, copyright 2011, Elsevier Ltd. All rights reserved.

To achieve precise spatial and temperature control in PTT, integrating imaging-guided technologies with photothermal nanoplatforms has become a research hotspot. For instance, Cheng *et al.* developed gold-coated UCNPs-iron oxide NP nanocomposites for dual-modal imaging via UCL and MRI-guided magnetically targeted PTT (Figure 15). *In vivo* imaging showed magnetic targeting yielded a 7-fold UCL signal increase in tumors versus non-targeted approaches. MRI confirmed an 8-fold rise in magnetic NP tumor accumulation. With magnetic targeting and NIR irradiation, all treated mice achieved complete tumor elimination without recurrence within 40 days and survived beyond 40 days, while controls averaged 14-18 days lifespan. Histology indicated nanocomposite accumulation in some organs caused no significant toxicity 294. Moreover, researchers also leverage UCL temperature sensitivity for nanoscale thermal monitoring. Ramírez-García *et al.* constructed UCNPs-AuNPs composites where 975 nm excitation converts light to visible wavelengths. Green light excites the SPR of AuNPs, generating photothermal effects. Five-minute irradiation increased temperature from 37 °C to 42.2 °C, reducing MCF-7 viability by over 60%. Green emission band ratios enable 25-50 °C temperature monitoring, while 659 nm red emission facilitates cell imaging and tracking, providing a multifunctional nanoplatform for imaging-guided temperature-controlled photothermal therapy of BC 295. Similarly, considering that prolonged treatment at 42-45 °C may damage normal tissues, Zhu *et al.* constructed a core-shell structured upconversion nanocomposite using a carbon shell as the PTA, enabling effective photothermal ablation of tumors *in vivo* while providing temperature feedback to reduce damage to normal tissues caused by overheating 296.

The combining of non-invasive PTT and PDT can achieve synergistic therapeutic performance with attenuated light power. In 2014, Chen *et al.* achieved a milestone by loading UCNPs with rose bengal (RB) and IR825, creating the first UCNP-based PTT-PDT system. Under 980-nm excitation, RB absorbed UCNP-emitted green light to generate cytotoxic ^1^O_2_ for PDT, while IR825 provided strong photothermal conversion under 808 nm irradiation for PTT. *In vitro*, combined therapy demonstrated superior cancer cell killing versus monotherapies, reducing viability to near zero. In 4T1 tumor-bearing mice, combined PDT-PTT yielded only 15% relative tumor volume compared to individual therapies 297. In Yang *et al.*'s study, a multifunctional core-satellite nanostructure was developed for combined PTT and PDT targeting microRNA-21 in MCF-7 cells. It employed the microRNA-21-triggered toehold-mediated strand displacement reaction to dissociate UCNP@Au. This dissociation enabled UCL restoration for *in situ* imaging of microRNA-21 and augmented ^1^O_2_ production for PDT, while the released AuNPs aggregated, resulting in a potent PTT effect. Under 808 nm laser irradiation, the combined PTT-PDT therapy significantly inhibited MCF-7 cells, reducing the survival rate below 40% 298. Another study by Chu *et al.* reported UCNPs@AgBiS_2_ NPs where concentration resonance between Nd^3+^ ions and AgBiS_2_ enhanced photothermal efficiency from 14.7% to 45%. Nd^3+^-doped UCNPs generated strong emissions exciting the AgBiS_2_ shell for ROS production. These NPs showed cancer-specific cytotoxicity against 4T1 cells under 808 nm irradiation. In tumor-bearing mice, NPs elevated tumor temperature to 56.3 °C, causing sustained growth inhibition with significant tissue damage and no systemic toxicity 299.

## 10. Smart Drug/Gene Delivery

Therapeutic agents aim to reach tumor sites via specific administration routes to trigger pharmacological responses. However, conventional formulations release immediately post-administration, with minimal amounts reaching lesions. Intrinsic physicochemical drug properties further limit efficacy. Commercial BC therapeutic drugs are broadly classified by water solubility into hydrophilic and hydrophobic categories (Table 7). Hydrophobic drugs, most common in BC, cannot cross aqueous phases, restricting intracellular target access. Hydrophilic agents face challenges including poor cellular uptake due to membrane penetration difficulty, rapid enzymatic degradation reducing bioavailability, and limited circulation times. These limitations often cause suboptimal therapeutic outcomes and systemic toxicity. For example, doxorubicin (DOX) associates with significant hematopoietic, gastrointestinal, and cardiac toxicities. Paclitaxel frequently causes neutropenia and peripheral neuropathy. Similarly, docetaxel, cisplatin, tamoxifen, and trastuzumab link to side effects like fatigue, weight loss, peripheral neuropathy, and nausea 300,301. Nucleic acid drugs show remarkable potential for treating genetic disorders, tumors, and viral infections, positioning as the third major therapeutic class after small molecules and antibodies. However, their large molecular weight and negative charge impede biological membrane passage. Moreover, RNA is highly susceptible to ribonuclease degradation and often becomes trapped in endosomal vesicles post-internalization, failing to exert functional activity 302.

Drug delivery systems address these physicochemical challenges. Engineered UCNPs are attractive candidates due to distinctive sizes, tunable surface functionalities, and regulated drug discharge. Encapsulating UCNPs with polymers, proteins, or mSiO_2_ provides drug/gene reservoirs while enhancing colloidal stability 303,304. Drug loading typically involves mixing UCNPs with agents in pH-specific solutions for certain durations. This method effectively encapsulates charged groups; positively charged coatings enhance gene loading efficiency. However, inert, uncharged, or hydrophobic groups often yield low encapsulation efficiencies 305. Other approaches like chemical conjugation, in-situ synthesis, and supramolecular assembly aim to increase drug loading, improve stability, and achieve targeted delivery 306,307. Recent understanding of TME and progress in materials engineering have enabled smart UCNP-based drug delivery systems. Unlike traditional methods, these platforms utilize responsive materials reacting to endogenous signals or exogenous stimuli. Activation triggers programmed structural reconstruction through chemical bond cleavage, phase transition, or pore opening, disrupting carrier-drug equilibrium for precise tumor-site release. Advances employing UCNPs for smart release systems are summarized in Table 8, with representative applications reviewed below.

### 10.1. Photo stimuli

Light-controlled delivery offers non-invasive characteristics and superior spatiotemporal precision. UCNP-assisted systems provide deeper tissue penetration via NIR excitation, minimized photodamage, no DNA/RNA harm, excellent biocompatibility, and robust photochemical degradation resistance. Efficient NIR-triggered systems require three elements: strong UCNP upconversion emission, effective photoresponsive material integration, and optimal NIR excitation parameters. Under NIR irradiation, photoresponsive components absorb UCNP-emitted visible/UV photons, initiating photochemical reactions that modify delivery system composition/conformation for controlled release.

**Figure 16:**
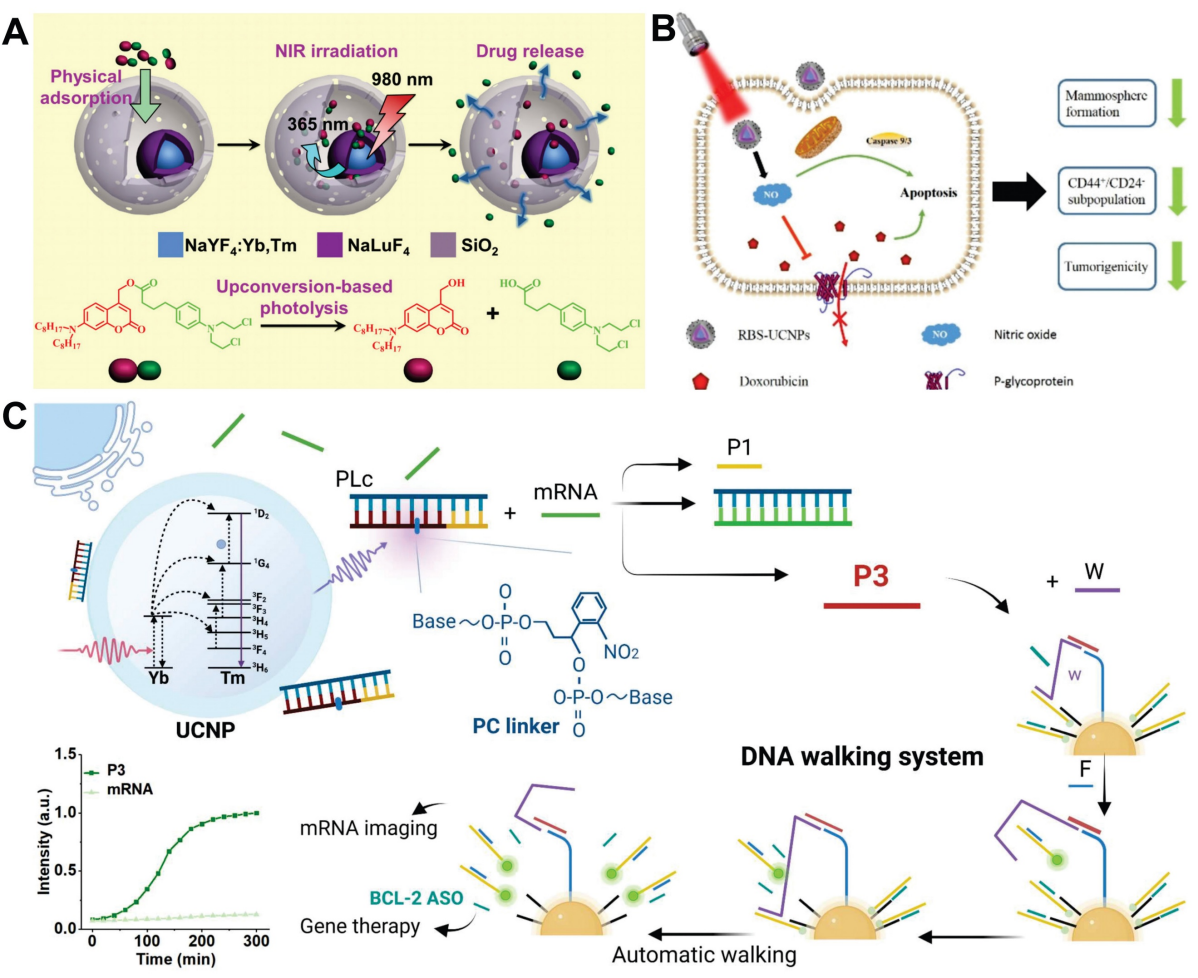
(A) Schematic illustration of the NIR-regulated upconversion-based phototrigger-controlled drug-release device and the photolysis of the prodrug under upconversion emission from the UCNPs. Adapted with permission from 308, copyright 2013, WILEY-VCH Verlag GmbH & Co. KGaA, Weinheim. (B) Schematic diagram of the synergistic treatment of NO released from NPs and DOX. Adapted with permission from 313, copyright 2017, Science China Press. Published by Elsevier B.V. and Science China Press. All rights reserved. (C) Illustration of Intracellular NIR photoactivatable NPs can released P3 DNA in the prescence of mRNA, and P3 can activated downstream operation of entropy-driven DNA walking system for survivin mRNA imaging and gene therapy with highly spatiotemporal resolution. The fluorescence dynamic curve on the left showed the P3-initiated DNA walking system. Adapted with permission from 316, copyright 2023, Elsevier Ltd. All rights reserved.

As a representative example, Zhao *et al.* designed a yolk-shell NaYF_4_:Yb,Tm@NaLuF_4_ nanocage with mSiO_2_ shell, loaded with hydrophobized amino-coumarin-caged chlorambucil (Figure 16A). Under 980 nm NIR light, the system released over 50% chlorambucil within 6 hours and 68% after 15 hours *in vitro*, with 88% photolysis efficiency. *In vivo* studies on S180 tumor-bearing mice showed significant tumor growth inhibition and prolonged survival after intratumoral injection and NIR irradiation. This is the first NIR-regulated drug release system demonstrated in living animals, overcoming the low tissue penetration of traditional phototriggered devices 308. Subsequently, Dcona and Matthew conjugated DOX to LiYF_4_:Tm,Yb NP surfaces via a photocleavable linker. Under 980 nm laser irradiation, NP emissions at 353/368 nm cleaved the nitroveratryl-glutamate linker to release DOX 309. In 2018, Han *et al.* used β-cyclodextrin as a gatekeeper to cap 2-diazo-1,2-naphthoquinones via hydrophobic interaction. Upon UV light illumination from UCNPs, the hydrophobic diazo-1,2-naphthoquinones were converted to hydrophilic 3-indenecarboxylic acid. Consequently, β-cyclodextrin dissociated from the UCNPs@mSiO_2_ surface due to repulsion between the hydrophobic cavities and hydrophilic guest, enabling the release of DOX from the unblocked pores 310. Another system used FA-conjugated photo-responsive copolymers encapsulating mSiO_2_-coated UCNPs for tumor-targeted DOX delivery. Under a 980 nm laser, the emitted UV light modified copolymer structure to trigger rapid release in FR-overexpressing cells 311.

Beyond small-molecule drugs, UCNPs-mediated NIR light-controlled systems have also demonstrated unique advantages in the field of gas signaling molecule controlled release. For example, endogenous messengers like NO regulate tumor vasculature and induce apoptosis, but their short half-life and diffusivity prevent spatiotemporal control. Coupling photosensitive gas donors with UCNPs enables deep-tissue NIR-triggered release, avoiding UV-associated damage. In 2015, Zhao's team integrated 980 nm-excited UCNPs with roussin's black salt, achieving controlled NO release where higher laser power triggered burst release activating apoptosis. Notably, low-dose NO reversed chemoresistance 312. Subsequent work used 808 nm-excited UCNPs conjugated with roussin's black salt for NO delivery combined with DOX, suppressing tumor stem cell proliferation and metastasis (Figure 16B). Blue/UV light from these UCNPs promoted NO release, inducing apoptosis and reversing resistance via P-glycoprotein downregulation. The system inhibited MCF-7 mammosphere formation and CD44^+^/CD24^-^ subpopulations *in vitro* while reducing tumorigenic potential of tumor stem cells *in vivo* 313.

Concurrently, UCNPs-mediated NIR light-controlled delivery systems have also demonstrated remarkable application potential in the field of nucleic acid therapeutics. Short interfering RNA (siRNA), a type of double-stranded non-coding RNA molecule, has emerged as a highly promising therapeutic agent, owing to its gene-regulatory capabilities. Specially, these molecules can silence specific genes, offering an innovative strategy to target previously undruggable signaling pathways in cancer treatment. However, the poor inherent stability of siRNA severely restricts its direct *in vivo* application, rendering it difficult to function as a standalone therapeutic agent. To expand therapeutic modalities, Zhang's team engineered mSiO_2_-UCNPs hybrids for NIR-controlled gene delivery, where UV-mediated cleavage of 4,5-dimethoxy-2-nitroacetophenone caging groups enabled precise release of functional plasmid DNA/siRNA 314. Yang *et al.* developed different upconversion nanoplatforms that siRNA was covalently and stably linked on the upconversion nanocrystals surface by cationic photocaged linkers. Following cellular internalization and 980 nm laser irradiation, the emitted UV light effectively triggered siRNA release, which was further confirmed by gene silencing assays 315. In another study, Xiang *et al.* developed an NIR-activatable DNA nanodevice integrating UCNPs with entropy-driven catalysis for mRNA imaging and antisense oligonucleotide release (Figure 16C). This platform demonstrated precise Bcl-2 antisense oligonucleotides delivery through UV-triggered DNA walker activation, inducing tumor apoptosis via anti-apoptotic protein downregulation without transfection reagents, as validated in both *in vitro* and *in vivo* models 316.

### 10.2. pH stimuli

Under normal physiological conditions, the pH value in the extracellular matrix and blood is nearly 7.4. However, due to the rapid proliferation of tumor cells and the generation of irregular blood vessels, the tumor site lacks nutrients and oxygen. This causes the accumulation of lactic acid produced by glycolysis of tumor cells in the tumor interstitium, reducing the pH value of the extracellular environment of tumor cells to 6.5-7.2, while the pH value of endosomes and lysosomes within tumor cells further decreases to 4.0-6.0 317,318. This micro-acidic environment is prevalent in various tumors and plays a vital role in the generation and development of tumors, especially in drug resistance 319.

**Figure 17:**
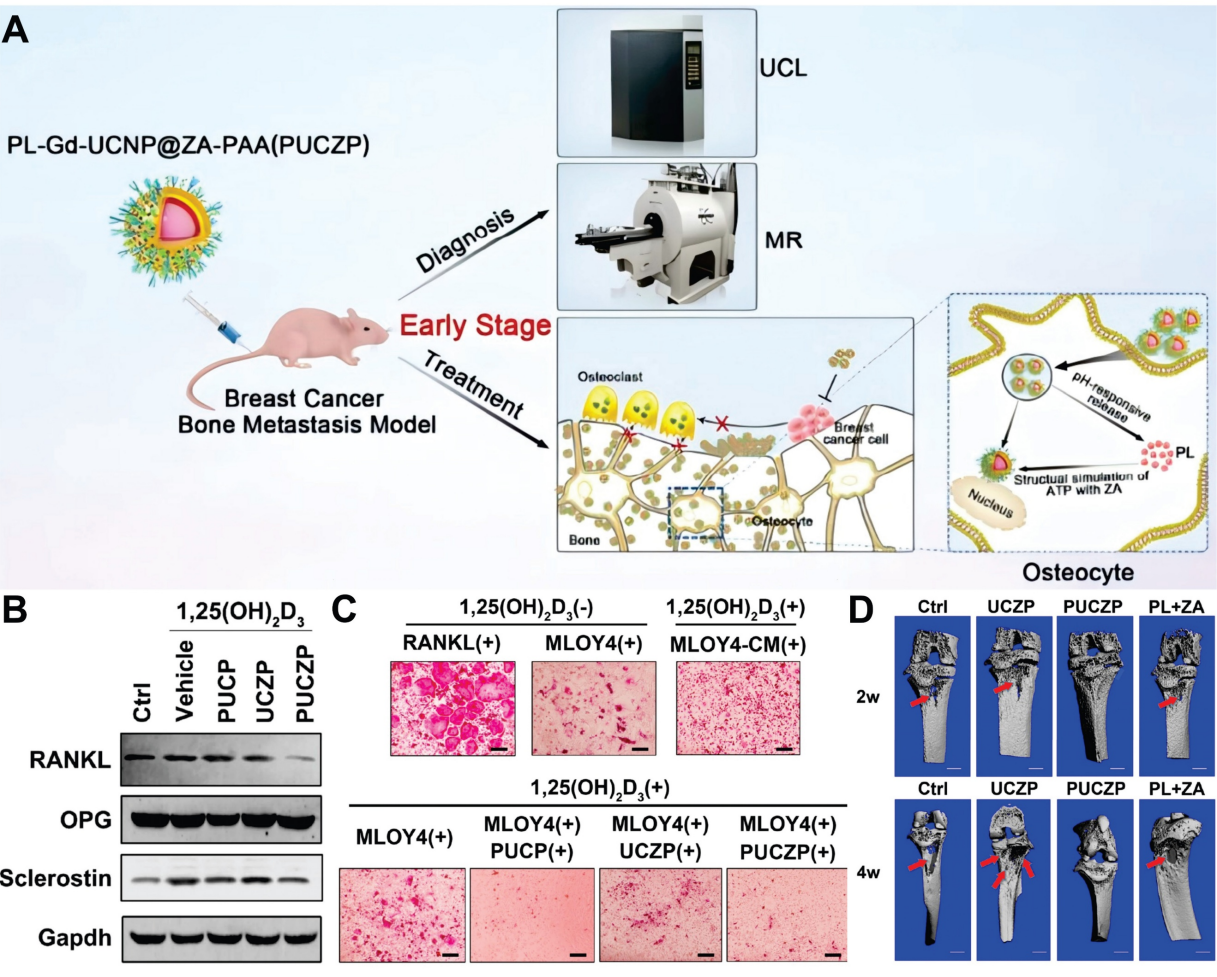
(A) Scheme illustration of the theranostic bone-targeting Gd (III)-doped UCNPs. (B) Western blot of RANKL, OPG, and sclerostin in MLOY-4 cells treated with varying NPs. (C) Murine bone marrow monocytes were treated with various interventions for 7 consecutive days. TRAP staining was used to indicate mature multinucleated osteoclast cells. (D) Representative μCT tomography of bone metastasis in specimens from nude mice treated with various NPs after 2/4 weeks. Red arrows indicate the osteolysis lesions. Adapted with permission from 320, copyright 2017, American Chemical Society.

In view of this, pH-responsive UCNPs delivery systems are designed to remain stable under normal physiological conditions. Once reaching the tumor site, they are triggered by the slightly acidic environment to undergo processes such as protonation, expansion, surface charge reversal, or chemical bond cleavage, thereby achieving the goal of drug release. For example, Qiao *et al.* developed a UCNP system targeting osteocytes that integrated zoledronic acid with pH-responsive plumbagin release for BC bone metastasis theranosis (Figure 17). The results showed that the NPs significantly reduced the expression of RANKL and sclerostin in MLOY-4 osteocytic cells and inhibited osteoclastogenesis induced by MLOY-4 cells. Additionally, the NPs markedly suppressed the proliferation, migration, and invasion of 4T1 and MDA-MB-231 cells, while promoting their apoptosis. Furthermore, the NPs enabled early detection of bone metastasis lesions via UCL and MRI. After 2 weeks of treatment, the bone volume/tissue volume ratio in the NPs group increased by 59.9% compared to the control group, and this ratio remained significantly higher after 4 weeks of treatment, demonstrating sustained therapeutic efficacy 320. In 2017, Chowdhuri *et al.* developed FA-encapsulated nanoscale metal-organic frameworks on the surface of UCNPs, forming a core-shell drug delivery system. This system exhibited negligible toxicity toward TNBC cells and normal NIH3T3 cells, enabling efficient encapsulation of DOX. Notably, approximately 30% and 40% of DOX were released at pH 7.4 within 12 and 24 hours, respectively, whereas 65% and 72% of DOX were released at pH 5.5 over the same time periods, ensuring preferential drug delivery to cancer cells and minimizing damage to normal cells. The DOX-loaded NPs efficiently internalized into MDA-MB-468 cells via FR-mediated endocytosis, exhibiting enhanced cytotoxicity toward cancer cells compared to normal cells 222.

In addition to precise control over drug release, the unique optical properties of UCNPs enable real-time monitoring of the drug release process. For instance, Hu *et al.* developed a pH-responsive drug delivery nanosystem by functionalizing metal-phenolic networks onto mSiO_2_-coated UCNPs. Tannic acid and Cu^2+^ coordination complexes were used to block DOX in the mesopores of UCNPs. Loading DOX induced FRET from UCNPs to DOX, leading to quenching of luminescence for monitoring drug release. Results showed that the cumulative release of DOX reached 51.1% at pH 5.0 and only 3.2% at pH 7.4. When networks degraded in acidic environments to trigger DOX release, FRET was eliminated and luminescence was restored, enabling real-time monitoring of pH-triggered drug release in cells. This study provides an effective strategy for designing smart drug delivery systems with both pH-controlled release and release monitoring functions 321. In another study, Wang *et al.* focused on the synergistic innovation of multiple responsive mechanisms, developing an NIR and pH dual-responsive drug delivery nanocomposite composed of UCNPs and a transformable poly(4,5-dimethoxy-2-nitrobenzyl methacrylate) shell layer. The nanocomposite exhibited a DOX loading efficiency of 7.23%, and the cumulative DOX release reached 59.5% within 300 minutes under the synergistic effect of pH 4.5 and 980 nm NIR light. The drug release kinetics were described by the Baker-Lonsdale model, confirming the dual-stimuli responsive release behavior and its potential for tumor-targeted therapy 322. Similarly, the nanoplatform designed by Chen *et al.* leveraged dual-responsive properties and UCL to enable on-demand drug release and dynamic monitoring of the therapeutic process 323.

### 10.3. Other stimuli

Beyond light and pH stimuli, smart drug delivery systems based on UCNPs can leverage various other signaling cues to achieve precise control over drug release. Glutathione (GSH), a tripeptide composed of glutamate, cysteine, and glycine, serves as the primary reducing ligand in biochemical processes 324. The concentration of GSH in the cytoplasm of tumor cells is higher than its extracellular concentration (ranging from 2 to 10 mmol/L compared to 2-20 μmol/L). Moreover, the GSH concentration in tumor tissues is at least four times higher than that in normal tissues 325. This distinctive feature has spurred the development of diverse GSH-responsive UCNP-based delivery systems, primarily utilizing disulfide bond cleavage and thiol exchange reactions 326,327. In the TME, GSH can also promote the biodegradation of Mn ions doped silica nanoshells, enabling GSH-responsive drug delivery. For instance, Xu *et al.* synthesized a Mn-doped mSiO_2_ nanoshell as a drug carrier, which rapidly degrades in the reductive and mildly acidic TME. This degradation process occurs via the sequential cleavage of Mn-O and Si-O bonds, allowing for efficient delivery of DOX to the tumor site. Notably, the Mn^2+^ released from the biodegradation process can enhance T_1_-weighted MRI contrast, providing diagnostic functionalities 328. Another key characteristic of tumors is their elevated temperature, a consequence of enhanced aerobic glycolysis and rapid cell proliferation. Therefore, temperature-sensitive polymers have been widely applied to regulate the release of antitumor drugs via their structural alterations in response to the temperature fluctuations within tumor tissues 329,330. Additionally, enzymes with specific high expression in tumor tissues can also serve as endogenous signals to trigger drug release 331.

## 11. Immunotherapy

Targeted immune system therapeutic strategies, including chimeric antigen receptor T-cell therapy, immune checkpoint blockade therapy, neoantigen vaccines, and small-molecule modulators, have emerged as highly effective approaches for treating various cancers 332,333. However, their clinical efficacy remains generally limited, benefiting only a minority of patients. Issues such as extensive adverse reactions, lack of reliable biomarkers, tumor recurrence, drug resistance, and metastasis also restrict broader clinical application. UCNP-based immunotherapeutic strategies offer significant advantages, enabling precise targeting, local drug release, combination with other therapies to enhance efficacy, and inherent imaging capabilities. Table 9 summarizes UCNPs combined with immunotherapy.

**Figure 18:**
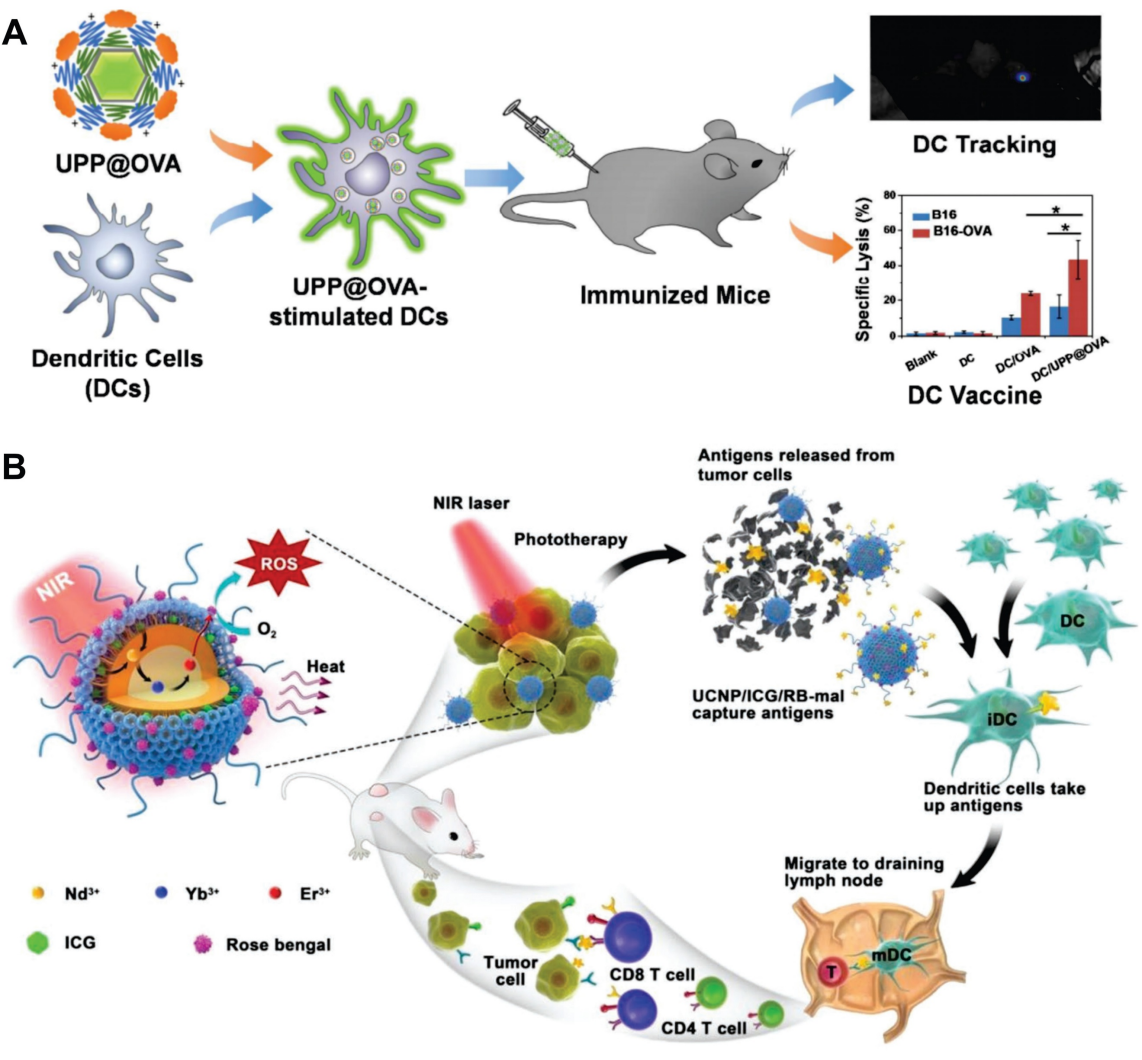
(A) Schematic illustration of antigenloaded UCNPs for DC stimulation, tracking and vaccination in DC based immunotherapy. Bipolymer-coated UCNP-PEG-PEI (UPP) NPs were synthesized and loaded with ovalbumin (OVA) via electrostatic interaction to form UPP@OVA complexes. These complexes were efficiently internalized by DCs, inducing DC maturation and cytokine secretion. The UCL property of UCNPs enabled highly sensitive *in vivo* tracking of DC migration, demonstrating homing of labeled DCs to draining lymph nodes. Compared with free OVA-pulsed DCs, UPP@OVA-pulsed DC vaccines significantly enhanced T cell proliferation, interferon-γ secretion, and cytotoxic T lymphocyte mediated responses, providing a novel trackable strategy for immunotherapy. Adapted with permission from 335, copyright 2015, American Chemical Society. (B) Schematic illustration of both fabrication and mechanism of NIR-triggered antigen-capturing nanoplatform for synergistic photo-immunotherapy. The NIR-triggered antigen-capturing nanoplatform was constructed via self-assembly of PEG and indocyanine green onto the oleate-capped UCNPs, followed by remote loading of RB. Upon NIR laser activation, the photodynamic therapy efficiency of NPs was significantly enhanced by indocyanine green modification, while simultaneously achieving selective PTT. Next, tumor-derived protein antigens arising from NPs based phototherapy can be captured and retained *in situ*, which increases the effects of antigen uptake by antigen-presenting cells to induce a tumor-specific immune response. Adapted with permission from 345, copyright 2019, under a Creative Commons CC BY license.

Dendritic cells (DCs) are central to initiating and regulating innate and adaptive immunity within the TME. They exhibit remarkable competence in antigen uptake, processing, and presentation. DCs differentiate into mature form upon antigen exposure and migrate to lymph nodes to activate T cells. This process is critical for DC vaccine efficacy 334. Conventional imaging methodologies cannot enable real-time visualization of DC migration dynamics. In 2015, Xiang *et al.* developed antigen-loaded UCNPs for DC stimulation, tracking, and vaccination (Figure 18A). They showed that dual-polymer-coated UCNPs efficiently delivered antigens into DCs through endocytosis, induced DC maturation and cytokine release, and enabled highly sensitive *in vivo* UCL imaging to track DC migration to draining lymph nodes, achieving a detection limit as low as 50 cells in mice. Notably, the UCNPs-pulsed DC vaccine elicited robust antigen-specific immune responses, including enhanced T cell proliferation, interferon-γ secretion, and cytotoxic T lymphocyte mediated cytotoxicity against tumor cells. These findings highlight the potential of UCNP-based DC vaccines for effective cancer immunotherapy 335.

Immunogenic cell death (ICD), which can be induced by various antitumor therapies, prompts the release of danger associated molecular patterns and tumor-associated antigens. This process facilitates the maturation of DCs and infiltration of cytotoxic T lymphocytes, thereby reversing the tumor immunosuppressive microenvironment and enhancing sensitivity to immunotherapy 336. Notably, UCNP-based drug delivery systems are being explored for inducing ICD through chemotherapy, PDT, and PTT, enabling multifunctional therapeutic integration. For example, Lin's team prepared mSiO_2_-coated UCNPs with large pores, loaded with PS MC540 and tumor antigens. Under 980 nm light irradiation, the nanocomposites mediated PDT via UCL, generating ROS to induce tumor cell death. Simultaneously, released antigens activated DCs, triggering robust Th1/Th2 immune responses. This was evidenced by increased cytokine secretion and elevated frequencies of CD4^+^, CD8^+^ and effector-memory T cells. In tumor-bearing mice, the nanovaccines showed synergistic therapeutic effects, more potently inhibiting tumor growth and prolonging survival than single PDT or immunotherapy 337. Jin *et al.* developed a nanocarrier system where UCNPs were coated with sialic acid-modified micelles and loaded with DOX and PS RB. This system enabled synergistic chemo-photodynamic therapy that induced ICD, evidenced by increased cell surface calreticulin exposure, extracellular ATP release, and high mobility group protein B1 secretion. In 4T1 tumor-bearing mice, the nanocarrier combined with NIR light promoted intratumoral infiltration of CD8^+^/CD4^+^ T cells, elevated anti-tumor cytokine levels, and inhibited tumor growth and lung metastasis 338. Another study reported that biodegradable K_3_ZrF_7_:Yb,Er NPs dissolved in cancer cells to release substantial K^+^ and [ZrF_7_]^3-^, triggering a surge in intracellular osmolarity and homeostasis imbalance. This further induced increased ROS, caspase-1 activation, gasdermin D cleavage, and interleukin-1β maturation. *In vivo* experiments confirmed these UCNPs enhanced DC maturation and effector memory T cell frequency, significantly inhibiting TNBC growth and pulmonary metastasis 339.

To enhance the immune response of immunotherapy, UCNPs are frequently integrated with immune adjuvants or immune checkpoint inhibitors 340-344. For instance, Wang *et al.* designed a nanoplatform by self-assembling PEG and indocyanine green onto UCNPs followed by loading RB, applying it to NIR-triggered photothermal, photodynamic, and immunotherapy for metastatic TNBC (Figure 18B). The nanoplatform effectively destroyed primary tumors and inhibited untreated distant tumors. When combined with anti-cytotoxic T-lymphocyte-associated protein 4 (CTLA-4) antibody, it enabled approximately 84% of treated tumor-bearing mice to achieve long-term survival and 34% to develop tumor-specific immunity, offering a promising approach for metastatic cancer treatment 345. Similarly, Liu *et al.* developed UCNPs-PEG nanocomposites for co-delivering PS and the immune adjuvant imiquimod (R837). Under NIR irradiation, the effective photodynamic destruction of tumors generated tumor-associated antigens. These antigens promoted strong antitumor immune responses in the presence of the R837-containing NPs acting as adjuvant. Furthermore, combining nanocomposite-mediated PDT with CTLA-4 checkpoint blockade exhibited remarkable efficacy. This strategy not only eradicated NIR-exposed tumors but also induced strong systemic antitumor immunity, inhibiting growth of distant tumors 346. In another study, Lin *et al.* designed a TME-activated UCNP-based enzymatic cascade nanocatalyst. This system enabled synergistic starvation/chemodynamic/immunotherapy by amplifying ROS generation, reversed the immunosuppressive TME, and allowed real-time therapeutic monitoring via UCL 347.

## 12. Clinical Translation of UCNPs

The theranostic platforms based on UCNPs offer a promising approach in BC management. By integrating diagnostic imaging and therapeutic functions, these platforms address the limitations of conventional methods. Their ability to enable deep tissue penetration, combined with the strategic integration of MRI, PET, and CT-compatible components, facilitates multimodal imaging. Meanwhile, these nanoplatforms demonstrate robust drug/gene delivery capabilities, supporting chemotherapeutic, gene, photodynamic, photothermal, and immunotherapeutic strategies. Stimuli-responsive release mechanisms further enhance spatiotemporal payload delivery precision, maximizing therapeutic efficacy while minimizing off-target toxicity. Crucially, modular functional design on a single NP enables personalized theranostic systems. However, no UCNP-based products are yet approved for clinical use, with most studies confined to *in vitro* cell studies or animal models. This clinical translation lag stems from insufficient integration of UCNPs basic research with translational applications, alongside common nanomedicine translation challenges. This section reviews UCNPs clinical translation aspects, discusses challenges and solutions, and outlines future directions.

### 12.1 Design and fabrication of UCNPs

The biomedical application of UCNPs exemplifies a compelling interdisciplinary field. Over the past two decades, a critical disparity has emerged: while most nanomaterial researchers hold academic backgrounds in chemistry or physics, they often lack specialized expertise in pharmacy and medicine. This knowledge gap contributes to a growing disconnect between research objectives and material design. Specifically, UCNPs have been extensively studied for uses such as molecular detection, *in vivo* imaging, drug delivery, and cancer therapy. Yet, researchers have disproportionately focused on developing novel NP designs. This focus often overlooks crucial questions about how and when nanoplatforms interact with tumors and the underlying reasons for preclinical trial failures. This trend is evident in the thousands of publications detailing UCNP synthesis, modification, and evaluations *in vitro* or animal models. Most prioritize material development and preliminary efficacy validation, placing limited emphasis on comprehensive safety, quality control, and thorough efficacy assessment. For example, a survey by the European Upconversion Network (COST Action CM1403) found that only 18% of 1,811 biomedical UCNP studies included toxicity or safety evaluations 155. Systematic preclinical pharmacokinetic/pharmacodynamic analysis and molecular mechanism exploration were even rarer. Consequently, this interdisciplinary imbalance leaves numerous "candidate materials" without the critical data needed for clinical translation. To prevent this disconnection between design intent and practical utility, future UCNP design must integrate interdisciplinary collaboration and prioritize genuine clinical needs from the outset.

UCNPs and their functionalized derivatives show significant potential for diagnostic and therapeutic applications. However, knowledge gaps exist about their complex biological interactions, demanding an application-tailored design approach. As regulatory agencies evaluate nanomedicines individually, reflecting the absence of universal design standards, validation should align closely with specific application requirements. For diagnostic UCNPs, achieving rapid *in vivo* clearance is crucial to minimize biological burden. This often leads researchers to employ degradable materials or reduce particle size for efficient renal or hepatic excretion. While smaller nanocrystal sizes facilitate clearance, they typically compromise quantum efficiency. Conversely, therapeutic UCNPs frequently require prolonged tumor retention, addressed through surface modifications extending circulation time and enhancing active targeting. For cancer imaging and therapy, intravenous administration of UCNPs under 500 nm is generally preferred, optimizing targeting while preventing vascular obstruction and reducing clearance via the mononuclear phagocyte system. Some studies indicate arterial administration can bypass hepatic filtration, increasing tumor accumulation. For lymph node tracing, subcutaneous injection of negatively charged particles in the 10-100 nm range proves optimal. Particles with this size and charge exhibit weak interactions with blood and lymph components while facing reduced interstitial transport barriers. Moreover, while a positive zeta potential facilitates gene loading, excessively high positive charge density can result in increased cytotoxicity. In summary, UCNP design is fundamentally application-specific, with no single paradigm suiting all purposes. Each application necessitates rigorous, case-by-case validation of its customized parameters. Although this context-dependency complicates standardization, it highlights the scientific rigor essential for successful clinical translation.

Scaling nanodrug production from lab batches to industrial manufacturing presents a major challenge in nanomedicine development, requiring good manufacturing practice (GMP)-compliant synthesis to ensure safety and efficacy. Engineered UCNPs face poor reproducibility and significant scale-up hurdles due to their complex compositions and multi-step syntheses. These difficulties are further compounded by the absence of standardized GMP frameworks for inorganic NPs. Addressing these issues requires two essential strategies. First, developing more efficient fabrication processes involves optimizing synthesis parameters like temperature and pressure control validated for GMP environments alongside automated production systems. This enhances process control ensuring each manufacturing step meets GMP-compliant protocols. Second, establishing unified quality standards necessitates standardized testing protocols including GMP-aligned in-process analytics and post-synthesis characterization. This ensures product consistency by integrating critical quality attributes into the manufacturing pipeline.

### 12.2 Targeting for metastases

Patients with BC typically die from metastases rather than primary tumors. However, most existing studies utilize subcutaneously implanted primary tumors. These models grow in isolation with less than 20-30% of the tumor surface contacting non-cutaneous host tissues, inadequately representing real metastatic disease where lesions spread to distant sites like lymph nodes, brain, bone, and lung. Therefore, recapitulating BC's natural behavior using syngeneic mouse models or genetically engineered models is essential for research targeting metastatic foci. For example, the 4T1 model in BALB/c mice, when properly induced through spontaneous metastasis from orthotopic or subcutaneous inoculation sites not via tail vein injection, establishes reliable metastatic models that authentically recapitulate natural dissemination and colonization processes within murine systems. Notebly, current UCNP-based lymph node targeting primarily relies on lymphatic drainage and BC-related targets. However, recent research demonstrates that leveraging receptors on antigen-presenting cells or addressins on high endothelial venules represents powerful targeting approaches for lymph nodes 348. Advanced BC brain metastasis often causes severe outcomes. The blood-brain barrier, composed of brain microvascular endothelial cells, restricts most systemically administered therapeutics from entering brain parenchyma, hindering effective brain tumor treatment. For instance, trastuzumab, a HER2-targeted antibody, shows limited efficacy against BC brain metastases due to poor blood-brain barrier permeability. Owing to their nanoscale size and structural modifiability, UCNPs emerge as promising candidates for crossing the blood-brain barrier and enabling targeted brain tumor therapy 349.

### 12.3 Diagnosis for BC

UCNPs offer distinct advantages for BC molecular imaging, lymph node tracing, and tumor marker detection, including large anti-Stokes shifts, photobleaching resistance, and low background fluorescence, yet face significant challenges. To achieve high quantum yield, Yb^3+^-sensitized UCNPs excited by 980 nm light are widely used. However, this approach encounters two primary obstacles. Primarily, biological tissues strongly absorb 980 nm light through water and melanin, attenuating excitation intensity during penetration and reducing efficiency. Secondly, continuous 980 nm irradiation causes tissue overheating, potentially damaging healthy cells. Alternative Nd^3+^-sensitized UCNPs excitable at 808 nm present promising solutions to these limitations 350. Additionally, UCNPs exhibit low nonlinearity with orders of 4-5, challenging super-resolution microscopy below 100 nm. Enhancing strategies include utilizing photon avalanche mechanisms, optimizing ion doping, refining core-shell structures, and engineering energy migration pathways 351.

Despite multimodal imaging capabilities, UCNP platforms face persistent technical hurdles. Physical interferences between imaging modes, particularly energy transfer conflicts between luminescent cores and surface conjugates, compromise signal fidelity and resolution. Addressing this requires smart nanoplatform engineering with computational model-guided energy optimization and interfacial isolation. Furthermore, disparities in signal intensity and resolution across modalities hinder accurate image registration and fusion, limiting diagnostic value. Clinically, multimodal imaging suits patient stratification but requires dedicated equipment per modality. Mechanically integrating spatiotemporally disparate results often fails to achieve complementary information while adding noise. Additionally, the prolonged blood circulation of UCNPs necessitates delayed scanning protocols, typically conducted several days post-injection. This temporal lag not only complicates patient logistics but also reduces clinical feasibility by increasing the risk of inter-scan variability and prolonging diagnostic workflows.

Current UCNP-based biosensors primarily target circulating tumor biomarkers, with less emphasis on tissue specimens. Due to the transient and fluctuating nature of circulating biomarkers in non-malignant conditions, diagnostic outcomes relying on them are often ambiguous. In contrast, tissue biopsy combined with IHC for molecular subtyping remains the gold standard in BC diagnosis. Studies show nanoprobe-spectral analysis improves detection accuracy for low-expression targets compared to IHC alone, addressing biomarker ambiguity. Significantly, as HER2-low BC gains focus for targeted therapy, the semi-quantitative nature of IHC leads to poor consistency in HER2 evaluation. Quantitative fluorescence intensity measurement via UCNP probes offers a solution. Additionally, clinical tissue sections are typically several micrometers thick. Excessively thick sections obscure cellular structures, while overly thin sections increase workload and missed diagnosis risk 352. Leveraging NIR lasers with deep tissue penetration for excitation, UCNPs could potentially enable compatibility with thicker sections.

### 12.4 Treatment for BC

UCNP-based phototherapy holds notable potential for BC treatment but faces critical limitations. NIR light penetration depth is limited to several millimeters. For instance, in human breast tissue, 835 nm NIR light penetrates about 3.54 mm, in adipose tissue about 2.79 mm, and in cancer tissue about 4.23 mm 13. Consequently, low-power NIR light cannot effectively reach deep-seated tumors, reducing therapeutic efficacy. Additionally, UCNPs suffer from inherently low upconversion efficiency. They generate insufficient excitation energy under low laser power, while high-power lasers risk photodamage to biological tissues. Specifically, prominent NIR-to-visible upconversion efficiencies for UCNPs are around 5% for irradiances below 100 W/cm^2^ 353. Most UCNP-based phototherapy applications require relatively high laser powers (1-4 W/cm^2^), far exceeding the American National Standards Institute safe thresholds of 330-350 mW/cm^2^ at 808/980 nm and 420 mW/cm^2^ at 1064 nm 276. Higher laser power is thus unsuitable for clinical use due to severe photodamage risks. To tackle these challenges, strategies aim to boost upconversion efficiency within safe laser limits and enhance light penetration. For example, dye-sensitized organic-inorganic hybrid systems show upconversion quantum yields up to 9.8% even in sub-optimized configurations 354. Another approach leverages the second near-infrared window (NIR-II, 1000-1700 nm), offering deeper penetration and higher permissible exposures. Precision core-shell structure design also promises improved efficiency. Moreover, as mentioned earlier, combining phototherapy with other treatments such as chemotherapy, immunotherapy, and radiotherapy can synergistically enhance efficacy. Furthermore, existing UCNP-based PTT overlooks peritumoral heat accumulation, risking damage to normal tissues. Exploring strategies for real-time thermal feedback and mild PTT is therefore necessary to ensure safety 355.

Current drug delivery platforms primarily focus on single chemotherapy agents. Co-delivering multiple anti-BC therapeutics could enhance efficacy and combat resistance. Additionally, using UCNPs to visualize drug release can improve precision. For instance, one study used NaYF_4_:Yb,Tm NPs as optical probes to monitor DOX release, correlating luminescence attenuation with drug dissociation 356. Moreover, upconversion superballs loaded with the PS ZnPc and superoxide dismutase 1 siRNA via mSiO_2_ coating enabled spatiotemporally controlled release of therapeutics and genes under 808/980 nm light irradiation, setting a paradigm for co-delivery of multiple therapeutics and real-time visualization 357. Recently, intraductal drug delivery strategies for BC, which involve administering drugs through mammary ducts, have garnered significant attention. This approach enables minimally invasive drug distribution via the ductal tree. Nanosystem-based delivery can maximize its advantages by prolonging tissue retention, enhancing targeting, improving cytotoxicity, and reducing dosing frequency 358. In the foreseeable future, leveraging their superior optical properties, UCNP-based intraductal strategies could overcome challenges like duct identification and catheterization, enabling precise local therapy. Despite the rising trend in gene therapy, UCNP-based gene therapy research remains limited. Structural optimization based on payload-specific design is crucial. For instance, cationic polymer coatings protect nucleic acids during transport, biomimetic surface modifications enable stable conjugation of proteins and antibodies, and mesoporous structures enhance encapsulation of hydrophobic compounds 359. Spatiotemporal control of release kinetics is vital, especially for gene editing tools requiring cytosolic delivery. Ideally, such systems promptly release cargo upon entering the cytosol, minimizing delays and ensuring high-efficiency editing. Moreover, optimizing delivery vehicle design to enhance biocompatibility and reduce off-target effects is also crucial for safe gene editing application.

### 12.5 Systematic assessment

The systematic evaluation of engineered UCNPs requires a full-process assessment of quality, safety, and efficacy, which necessitates integrating multi-dimensional characterization technologies and interdisciplinary research frameworks. Traditional quality evaluation methods for UCNPs, including dynamic light scattering, transmission electron microscopy, X-ray diffraction, and fluorescence spectroscopy, primarily characterize physicochemical parameters post-synthesis and pre-application. However, given the complex nano-bio interactions, *in vivo* quality assessment of UCNPs is critical. Tracking structural evolution trajectories across diverse physiological and pathological stages to establish spatio-temporal correlations between structural parameters and biological effects can reveal nanomaterial dynamics *in vivo*. For example, fluorescence spectral analysis enables real-time monitoring of luminescence stability and spectral shifts, clarifying how anti-Stokes emission properties are influenced by protein corona formation or microenvironmental pH fluctuations.

Structure-activity relationships (SARs), which define the inherent correlation between molecular structures and biological outcomes (e.g., efficacy and toxicity), form the theoretical foundation for drug design, optimization, and mechanism elucidation. Establishing clear SARs for UCNPs remains challenging due to their multi-scale complexity, synergistic multi-factor influences, and interference from dynamic *in vivo* environments. This complicates biological effect prediction, quality control, mechanistic analysis, and material optimization. Specifically, the structures of UCNPs encompass not only molecular-level chemical compositions but also nanoscale physical properties (e.g., size, morphology, and surface charge). The biological effects of UCNPs arise from the combined action of multiple structural factors, making it difficult to disentangle the influence of a single component. Notably, once UCNPs enter the body, they undergo dynamic interactions with blood proteins, cell membranes, and tissue microenvironments. Their "actual structure" (e.g., the composition of the surface protein corona) may differ significantly from the as-designed structure, further blurring the correlation between the original structure and biological effects.

While most studies claim acceptable toxicity for engineered UCNPs, many lack dedicated toxicity evaluations and comprehensive characterization of key parameters, including spatio-temporal biodistribution, long-term accumulation kinetics, and transgenerational effects. Similar to other inorganic nanomaterials, most UCNPs are non-biodegradable, with only partially sized and charged NPs excreted via hepatorenal pathways 281. Prolonged retention in the mononuclear phagocyte system may pose risks. Future efforts should prioritize developing biodegradable UCNPs and balancing functional durability with controllable *in vivo* clearance through surface modification and biomimetic strategies. Notably, quantitative nanomaterial risk assessment remains infeasible due to absent standardized, validated methods for defining human or environmental exposure limits.

Current safety and efficacy evaluations rely predominantly on cell lines and rodent models, which suffer from translational limitations due to species-specific differences in metabolism, immunity, and barrier structures. Shifting to non-human primate models can mitigate such discrepancies. Additionally, integrating patient-derived xenografts and biomimetic *in vitro* 3D tumor models (e.g., tumor organoids) maximizes recapitulation of human BC heterogeneity and *in vivo* microenvironments 360. To demonstrate superior clinical efficacy or safety, large-scale, long-term clinical trials are essential. Identifying endpoints that meaningfully reflect patient benefits, such as improved long-term remission, delayed disease progression, or reduction of side effects, is critical for ensuring new nanomedicine products show sufficient clinical potential in pivotal trials. Tightening inclusion criteria to reduce patient variability in study protocols can downsize trial scales, though this introduces challenges like slower enrollment and narrower patient representation. One solution is using biomarkers to predict treatment responses, which diminishes variability impacts while failing to address enrollment delays.

### 12.6 Regulatory requirements of nanomaterials

The rapid advancement of nanotechnology in pharmaceuticals, cosmetics, and other sectors has outpaced the update of regulatory frameworks. The unique properties of nanomaterials render traditional regulatory standards for bulk materials inapplicable to them. For instance, gold nanoclusters exhibit catalytic activity, whereas bulk gold is inert. Additionally, controversies exist regarding the size-dependent toxicity of ZnO NPs toward crustaceans. These disparities make assessment systems based on bulk materials unable to accurately predict the biological effects of nanomaterials, thereby giving rise to challenges such as the inapplicability of safety and efficacy data and the fragmentation of global classification standards.

In response to these challenges, major countries and regions have adopted distinct regulatory strategies. For example, the European Medicines Agency applies general pharmaceutical legislation to nanomedicines and references the European Commission's 2011 recommendations on nanomaterials. However, these recommendations lack legal binding force and have not been uniformly implemented across the European Union 361. The FDA employs a "case-by-case approval" model, issuing the Drug Products, Including Biological Products, That Contain Nanomaterials Guidance for Industry in 2017 and the Liposome Drug Products Chemistry, Manufacturing, and Controls Human Pharmacokinetics and Bioavailability and Labelling Documentation Guidance for Industry in 2018. These guidelines emphasize the evaluation of physicochemical properties and pharmacokinetics of nanomedicines 362. The Medicines and Healthcare products Regulatory Authority of the United Kingdom currently lacks specific legislation for nanotechnology products, relying instead on laws of the European Union and domestic health and safety regulations.

Furthermore, international organizations are actively promoting regulatory coordination. The International Organization for Standardization has issued standards defining core terms in nanotechnology. The Organization for Economic Co-operation and Development has advanced the international harmonization of safety testing methods for nanomaterials. The International Council for Harmonization of Technical Requirements for Pharmaceuticals for Human Use has facilitated the exchange of regulatory science in nanomedicines through the International Pharmaceutical Regulators Programme. Adjustments to nanomaterial regulation must balance innovation and safety, gradually constructing a regulatory framework adapted to the characteristics of nanotechnology through international standard-setting, regional regulatory coordination, and interdisciplinary research. Future efforts should further strengthen global collaboration to address the complex challenges posed by nanomaterials in medicine, the environment, and other domains.

### 12.7 Clinical translation roadmap

**Figure 19:**
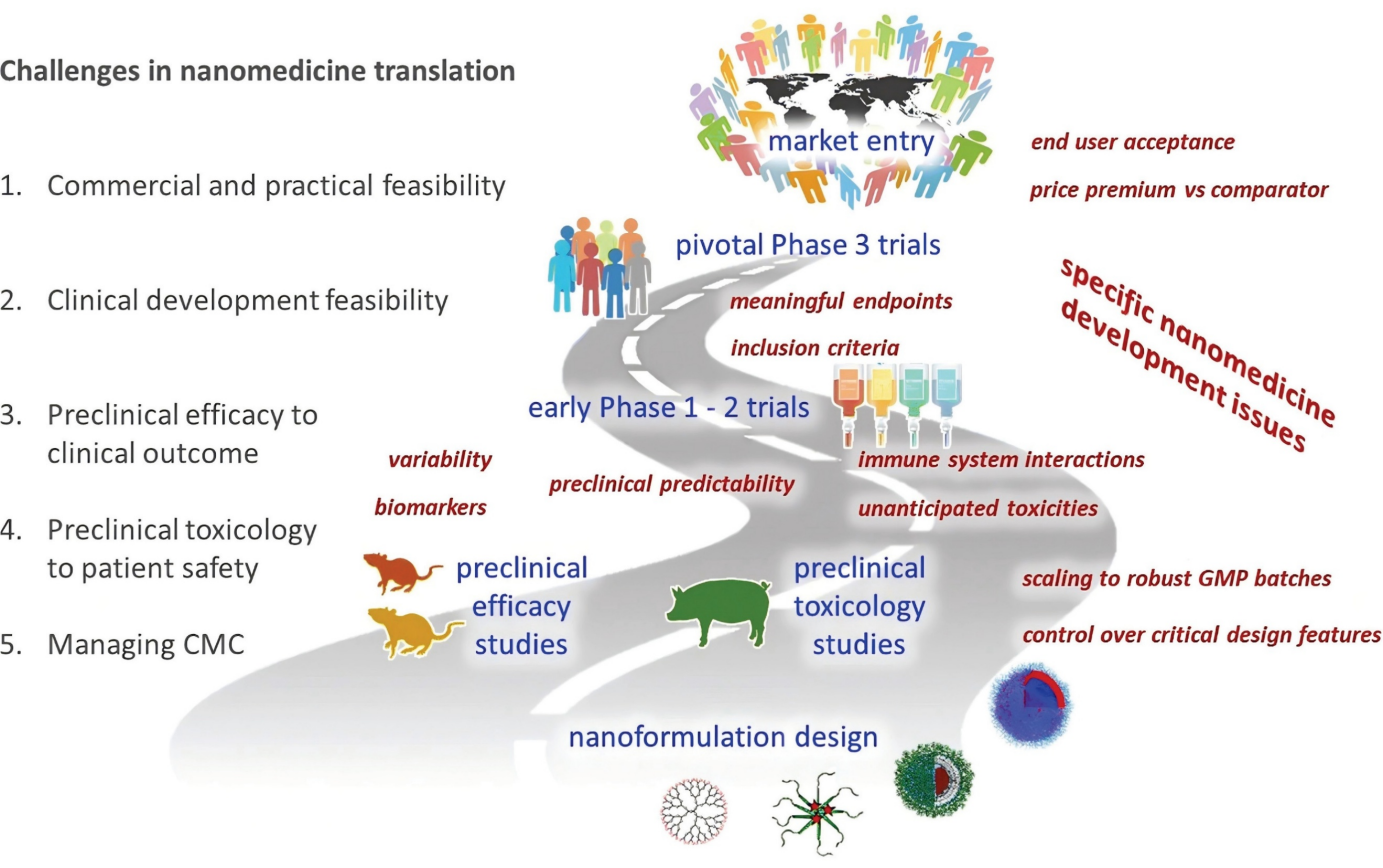
Challenges in nanomedicine clinical translation. Key translational and industrial aspects of nanomedicine product development are depicted. Challenges are traditionally approached in a bottom-up manner. However, also considering challenges in a top-down manner, from the vantage point of end-users, with commercial, practical, and clinical feasibility firmly in mind, is considered to be important for ensuring success. The top-down analysis allows for the identification-from the initiation of the clinical translation process onwards-of the most important issues that can be encountered along the way, triggering proactive thinking and planning to overcome potential challenges already at early stages. Adapted with permission from 363, copyright 2020, under a Creative Commons CC BY license.

Similar to other nanomedicines, the clinical translation of UCNPs should always be regarded as the ultimate development goal. Traditionally, the development and translation of cancer nanomedicines have adopted a bottom-up approach, starting from the design, manufacturing, and controls of nanoplatforms. However, in terms of feasibility, efficiency, and industrialization, a top-down approach is considered a wiser and more valuable strategy. This approach begins with an assessment of commercial potential, followed by an evaluation of clinical feasibility, and finally progresses to drug design, production, and quality control (Figure 19) 363.

Specifically, the first hurdle in developing any nanomedicine is evaluating its commercial viability, which hinges on the potential to improve patient outcomes and the size of the target patient population. Compared to benchmark products, UCNP formulations must offer advantages such as reduced dosing frequency, more convenient administration routes, improved therapeutic efficacy, reduced toxicity, or augmented patient benefits. The more pronounced these advantages, the easier it is to justify price premiums once the product enters the market. This requires an end-user perspective to identify products and trajectories with genuine clinical and commercial potential.

Concurrently, preclinical efficacy experiments must be meticulously planned, employing appropriate models to simulate human cancer progression for accurate evaluation of the antitumor activity of UCNPs. Toxicology studies should proceed in parallel, comprehensively investigating short-term acute toxicity, long-term chronic toxicity, and potential toxic effects on major organs. During experimental design, proactively focusing on critical issues likely to emerge in clinical development is essential, such as the predictability and controllability of UCNPs in their distribution, metabolism, and excretion, as well as uncertainties regarding immune response risks or unexpected tissue damage. In-depth research and data accumulation enable early identification and resolution of these issues, paving the way for subsequent clinical trials and ensuring high success rates and safety when UCNPs enter human testing.

In the drug design phase, considerations must extend beyond therapeutic efficacy to include formulation stability and manufacturability. The selection of nanomaterials and drug loading strategies requires iterative optimization to ensure stability during storage and transportation, as well as precise *in vivo* release. Strict standard operating procedures must be established during production to ensure each batch of UCNPs is manufactured according to predefined design criteria, guaranteeing quality consistency. Quality control systems should cover the entire process from raw material procurement to finished product testing, employing advanced analytical techniques and equipment to strictly monitor critical quality attributes, ensuring that product safety and efficacy meet clinical requirements.

In summary, top-down analysis identifies the most critical issues in the clinical translation process from the outset, triggering proactive thinking and planning to overcome potential challenges at early stages. This approach aligns with the interdisciplinary strategies emphasized by the European Upconversion Network (COST Action CM1403), focusing on key aspects such as nanotoxicity research, surface functionalization, and instrumentation development to ensure safety while maximizing clinical value in cancer theranostics 155.

## 13. Conclusion

Engineered UCNPs have garnered significant attention in the field of BC diagnosis and therapy due to their unique optical properties, excellent biocompatibility, and flexible design. These NPs not only serve as highly efficient imaging probes but also enable highly selective and smart therapeutic strategies. Current preclinical studies, including both *in vitro* cellular experiments and tumor animal models, have demonstrated the outstanding theranostic efficacy of these materials. However, the translational process from laboratory research to clinical application faces numerous challenges at the scientific, technical and regulatory levels. In the future, efforts should focus on optimizing the synthesis, functionalization, and clinical translation of UCNPs. Meanwhile, promoting collaborative and multidisciplinary approaches will help break through the limits of possibility, paving the way for UCNPs to play a core role in the theranostics of BC.

## Figures and Tables

**Table 1:** Advantages and disadvantages of common fluorescent probes.

Probes	Examples	Advantages	Disadvantages	Ref.
UCNPs	NaYF_4_:Yb,Er; NaGdF_4_:Yb,Tm	Large anti-Stokes shift; Narrow emission spectra; Long fluorescence lifetime; Strong stability	Low quantum yield; Potential biotoxicity	364
Fluorescent proteins	GFP; RFP; BFP	High biocompatibility; Genetically encodable	Poor stability; Shallow tissue penetration; Moderate quantum yield	365
Organic dyes	Cy5; FITC; ICG	High quantum yield; Ease of functionalization and bioconjugation	Poor stability; Biotoxicity; Broad emission spectra	366
Quantum dots	CdSe@ZnS; CdTe; InP@ZnS	High quantum yield; Broad excitation spectra; Narrow tunable emission; Strong stability	Heavy metal toxicity; Photoblinking	367
Metal complexes	[Ru(bpy)_3_]^2+^; Ir(ppy)_3_	Long fluorescence lifetime; Strong stability; Tunable redox activity	Heavy metal toxicity; Poor stability; Shallow tissue penetration	368
Carbon dots	Nitrogen-doped carbon dots; B-doped carbon dots	Low biotoxicity; Strong stability	Moderate quantum yield; Broad emission spectra; Limited functionalization and bioconjugation capacity	369

GFP: green fluorescent protein; RFP: red fluorescent protein; BFP: blue fluorescent protein; Cy5: cyanine 5; FITC: fluorescein isothiocyanate; ICG: indocyanine green.

**Table 2:** Cell viability after incubation with UCNPs.

UCNPs	Surface modification	Size (nm)	Zeta potential (mV)	Exposure time (h)	Concentration (μg·mL^-1^)	Viability	Cell line	Ref.
Ba_2_GdF_7_:Yb,Er	PEG	24±5	25.73	24	5000	>85%	HepG2	163
BaYbF_5_:Tm	PAA	14.7±3.5	-19.8	24	0.1-1000	>90%	HepG2	370
				48	0.1-1000	>90%		
NaYF_4_:Gd,Yb,Er	Citrate; ^18^F	28.2	18.1	4	100-500	>89%	KB	371
				24	100-500	81%		
Lu_2_O_3_:Gd,Yb,Er	PEG	85-130	-0.65	48	1000	>90%	MCF-7	372
NaLaMgWO_6_:Yb,Er	-	105	-	48	50-200	100%	WI⁃38	373
NaGdF_4_:Tm,Er,Yb	Azelaic acid (carboxylic acid)	25-60	-	4	62.5-500	>90%	KB	374
				12	62.5-500	>90%		
NaGdF_4_:Yb,Er	PEI; Phycocyanin	260.6±7.3	8.1±1.2	24	12.5-200	>80%	RAW264.7	375
				24	12.5-200	>80%	HeLa	
NaYF_4_:Yb,Er	PEI; FA;	7	-55	24	750-12000	90%	HeLa	376
LaF_3_:Yb,Ho	PEG	15	-	4	125-500	80%	KB	377
				12	125-250	>80%		
				24	125-250	>80%		
NaYF_4_:Tm,Yb @NaYF_4_:Nd,Yb	SiO_2_; MMP2-sensitive peptide; CuInS_2_/ZnS quantum dot	30	-	72	1-250	100%	Cal27	378
					1-250	>90%	FADU	
					1-250	>90%	OEC-M1	
NaLuF_4_:Yb,Er	Polypyrrole; PEG; DNA; DOX	70	4.6	48	200-1000	>80%	HEK293T	379
					200-1000	>80%	HeLa	
NaYF_4_:Yb,Tm	PEG; Metal-organic framework	500	-	24	25-1000	>90%	MCF-7	380
K_0.3_Bi_0.7_F_2.4_:Yb,Er	SiO_2_; Polyvinylpyrrolidone; Methylene blue	50	-	24	75-100	90%	C6	381
Y_2_O_3_:Er,Yb	FA; SiO_2_	70±10	-11.8±4.30	24	0.001-1	>80%	HeLa	219
						>80%	MDA-MB-231	
						>80%	MCF-7	
NaYF_4_:Yb,Tm@NaYF₄	4T1 cell membrane; Semiconductor material	110	-	24	25-150	>90%	L929	382
NaYF_4_:Yb,Er@NaYF_4_:Nd@NaYF_4_	Hydrogen-bonded organic framework materials; PAA	48.2	-12.97	24	37.5-600	>90%	4T1	383
NaYF_4_:Yb,Tm@NaYF_4_@NaYF_4_:Yb,Nd	SiO_2_; Covalent organic framework	421.0±40.3	10.8±2.5	24	10-200	100%	BJ	384
				48	10-200	100%	BJ	
				72	10-200	110%	BJ	

PEG: polyethylene glycol; PAA: polyacrylic acid; PEI: polyethylene imine; FA: folic acid; MMP: matrix metalloproteinase; DOX: doxorubicin.

**Table 3:** Safety studies of UCNPs in rodents.

UCNPs	Surface modification	Size (nm)	Zeta potential (mV)	Animal	Route	Dosage	Results	Ref.
Ba_2_GdF_7_:Yb,Er	PEG	24±5	25.73	Nude mice (25 g)	Tail vein injection	2-4 mg/mL (0.2 mL)	Major organ pathological sections normal; Hemolysis assay confirmed no blood cell damage	163
BaYbF_5_:Tm	PAA	14.7±3.5	-19.8	Kunming mice	Oral	100 mg/mL (0.4 mL)	Major organ pathological sections normal; Female mice litter size unchanged	370
					Anus	20 mg/mL (0.8 mL)	Rectal mucosa epithelium intact, no ulcers or inflammatory cell infiltration;	
				Wistar rats	Oral	20 mg/mL (5 mL)	Blood concentrations of Ba^2+^, Yb^3+^ < 0.1 ng/mL	
					Anus	20 mg/mL (5 mL)	*In vitro* hemolysis rate < 0.5%	
Lu_2_O_3_:Gd,Yb,Er	PEG	85-130	-0.65	Kunming mice	Tail vein injection	100 mg/kg	Body weight stable; normal diet, activity, and hair; major organ pathological sections normal; Hematological and biochemical parameters normal; Female mice litter size unchanged	372
NaGdF_4_:Tm,Er,Yb	Azelaic acid (carboxylic acid)	25-60	-	Kunming mice (20 g)	Tail vein injection	1.5 mg/kg	No significant weight loss, abnormal behavior, or reproductive changes	374
NaLuF_4_:Yb,Er	Polypyrrole; PEG; DNA; DOX	70	4.6	BALB/c nude mice (16-18 g)	Intravenous injection	9.4 mg/kg	Major organ pathological sections normal; Hematological and biochemical parameters normal	379
NaLuF_4_:^153^Sm,Yb,Tm	6-aminohexanoic acid	25-30	-	Kunming mice (20 g)	Tail vein injection	20 mg/kg	Major organ pathological sections normal; Hematological and biochemical parameters normal	385
Gd_2_O_3_:Yb,Er	PEG	90-150	-	Kunming mice	Tail vein injection	10 mg/kg	Body weight gain patterns normal; Mild inflammation in liver/spleen tissues; Hematological and biochemical parameters normal	66
NaLuF_4_:Yb,Er	Polyaniline NPs; Pluronic F127	120	0	NU/NU nude mice	Intravenous injection	2 mg/mL (0.1 mL)	Major organ pathological sections normal; Biochemical and renal parameters normal	386

PEG: polyethylene glycol; PAA: polyacrylic acid; PEI: polyethylene imine; FA: folic acid; DOX: doxorubicin; NPs: nanoparticles; Pluronic F127: poly (ethylene glycol)-block-poly (propylene glycol)-block-poly (ethylene glycol).

**Table 4:** Applications of UCNPs in BC molecular imaging.

Application	UCNPs	Surface modification	Size (nm)	Zeta potential (mV)	Results	Ref.
UCL imaging	NaYF_4_:Yb,Er	Recombi-nant scFv4D5 mini-antibody; Poly(maleic anhydride-alt-1-octadecene)	120±20	-53	Showed specific binding and uptake to SK-BR-3 cells; Predicted UCNPs-assisted cancer detection feasible at up to 4 mm tissue depth	206
UCL imaging	NaYF_4_:Yb,Er	Methylene blu; Liposomes; PEG; Anti-HER2 peptides	90±1.92	-18.3±1.56	Showed specific binding and uptake to SKBR-3 cells	202
UCL imaging	NaYF_4_:Yb,Er	Zinc tetracarboxyphenoxy phthalocyanine; Trastuzumab	23	-14.5±4.3	Showed specific binding and uptake to SKBR-3 cells	216
UCL imaging	NaYF_4_:Yb,Er	FA-PEG-poly(aspartic acid-hydrazone)-dihydrolipoic acid; Pheophorbide a	90.3	-	Showed specific binding and uptake to MCF-7 cells	221
UCL imaging	Y_2_O_3_:Yb,Er/Gd_2_O_3_:Yb,Er	SiO_2_; FA	70/50	-	Showed specific binding and uptake to MCF-7 cells	220
UCL imaging	Y_2_O_3_:Yb,Er	SiO_2_; FA	70	-11.8±4.30	Showed specific binding and uptake to MCF-7 and MDA-MB-231 cells	219
UCL imaging	NaYF_4_:Yb,Er	Nanoscale metal organic framework; FA	180±20	-	Showed specific binding and uptake to MDA-MB-468 cells	222
UCL imaging	NaYF_4_:Yb,Er,Tm	Arginine-glycine-aspartate; PEG	25.8	-	Showed specific binding and uptake to U87MG cells; Maximum binding of the material in U87MG tumors occurred at 4 hours after tail-vein injection and persisted until 24 hours; The SNR was about 24; Detected no autofluorescence signal even at 600 μm depth	223
FL imaging	NaErF_4_@NaYF_4_	Cyclic Arg-Gly-Asp sequence-containing pentapeptide c; PEG	61.3	-	Showed specific binding and uptake to 4T1 cells; Imaging penetration depth 9 mm; SBR at 5 mm depth fourfold ICG; Effectively distinguished malignant from normal tissues, identified microtumors, and guided complete tumor resection during surgery	224
FL/MRI imaging	NaGdF_4_:Nd@NaLuF_4_	PEG	32.7	-	The fluorescence signal peaked at 4 h post-injection, with a TBR of 8.2 at 1340 nm; The 4T1 tumor MRI signal was enhanced by 1.46-fold at 6 h post-injection	226
UCL/MRI imaging	NaGdF_4_:Yb,Er@NaGdF_4_:Yb@NaGdF_4_:Yb,Nd	mSiO_2_; Site-specific peptide; Chlorin e6	157.7	12.4	Showed specific binding and uptake to MDA-MB-435 cells; Showed good T_1_-weighted MRI performance	387
UCL/MRI/CT imaging	LiLuF_4_:Yb,Er@nLiGdF_4_	PEG; mSiO_2_; The Y₁ receptor ligand	106.7	-12.7	Showed specific binding and uptake to MCF-7 cells; Longitudinal relaxivity far higher than traditional MRI contrast agents, showing strong T_1_ contrast	228
UCL/MRI/PET imaging;	NaGdF_4_:Yb,Tm	Red blood cell membrane; PEG; FA	138.9	-12.1	Showed specific binding and uptake to 4T1 cells; UCL signal persisted ≥48 h; Enhanced tumor MRI signal	208
UCL/MRI/PET imaging	NaGdF_4_:Yb,Tm@NaGdF_4_	MDA-MB-231 cell membrane; PEG; ^18^F	200	-10 to -20	Showed specific binding and uptake to MDA-MB-231 cells; UCL signal persisted ≥48 h	235
UCL imaging	NaGdF_4_:Yb,Tm,Ca@NaLuF_4_	PEG; Anti-HER2 monoclonal antibody	137	-0.7	Showed specific binding and uptake to SK-BR-3 cells; Blood half-life 421 min; Tissue penetration depth 7.7 mm; Metastatic lymph nodes revealed	238
FL imaging	NaErF_4_@NaYF_4_	PAA; Balixafortide	18.6±0.8	-23.41	Fluorescence intensity stable ≥7 days in various solutions; Showed stronger optical stability (vs. ICG) under continuous laser, deeper penetration; Sentinel lymph node metastasis accurately detected	239
UCL/PET/SPECT imaging	NaGdF_4_:Yb,Tm@NaLuF_4_	PEG; Anti-HER2 monoclonal antibody; ^68^Ga; ^177^Lu	22.5±2.94	12.63	Showed specific binding and uptake to SK-BR-3 cells; Reduced lymph node metastasis, inhibited tumor growth	201
UCL imaging	NaYF_4_:Gd,Yb,Er@NaYF_4_	Cyanine 3; Anti-EGFR antibody; PEG; Atrix metalloproteinas 2 substrate peptide labeled with QSY7 quencher	85.7±2.3	10.8±1.5	Metastatic lymph nodes revealed	240

PEG: polyethylene glycol; FA: folic acid; UCL: upconversion luminescence; FL: fluorescence; PET: positron emission tomography; SPET: single-photon emission computed tomography; CT: computed tomography; SNR: signal-to-noise ratio; SBR: signal-to-background ratio; ICG: indocyanine green; TBR: tumor-to-background ratio; PAA: polypropylene acid.

**Table 5:** Applications of UCNPs in BC biomarker detection.

UCNPs	Surface modification	Size (nm)	Zeta potential (mV)	Biomarkers	Results				Ref.
					Linear range	Limit of detection	Recovery	Relative standard deviation	
NaYF_4_:Yb,Tm	PEG; Streptavidin	78.7	-	HER2	SBR=319; UCNP labeling compatible with H&E staining; Signal stable within 20 min				245
NaYF_4_:Yb,Tm	PEG; Streptavidin	44.2±4.0	-	HER2	SBR=319				246
NaGdF_4_:Yb,Er@NaGdF_4_	SiO_2_; NPTAT-doped SiO_2_; Anti-PR antibody	39.24±1.22	-	PR	Excellent correlation with WB/IHC; More accurate low-level protein quantification vs. IHC; Enabled multiplex *in situ* biodetection				247
NaGdF_4_:Yb,Tm@NaGdF_4_	SiO_2_; NPTAT-doped SiO_2_; Anti-ER antibody	38.14±1.22	-	ER					
NaYbF_4_:Er@NaYF_4_	SiO_2_; Rhodamine B isothiocyanate-doped SiO_2_; Anti-HER2 antibody	24.22±1.50	-	HER2					
NaYbF_4_:Er	Aptamer	6-7	-	VEGF	50-2000 pM	6 pM	98.0%-113.0%	2.9%-3.6%	248
NaYbF_4_:Er	PAA; Aptamer	50	-	VEGF_165_	0.1-16 ng/mL	0.1 ng/mL	96.5%-112%	<6.2%	249
NaYF_4_:Yb,Tm	PAA; 6-Carboxyfluorescein; Aptamer	43.4	-	CA15-3	0.01-150 U/mL	4.5 mU/mL	98.0%-105.0%	<5%	253
NaYF_4_:Yb,Tm	PEI; Anti-CA125 antibody	20	17	CA125	5-100 ng/mL	120 pg/mL	-	-	255
NaYF_4_:Yb,Er	PAA; Aptamer	41.7±3.6	-	CA125	0.01-100 U/mL	9 mU/mL	98%-102%	<6%	256
NaYF_4_:Yb,Er	PAA; Anti-CEA antibody	55	-	CEA	0.5-30 ng/mL	0.36 ng/mL	-	-	259
NaYF_4_:Yb,Tm	PDA	36	-13.1	CEA	2-200 ng/mL	0.1 ng/mL	96.7%-104.8%	<5%	260
NaYF_4_:Yb,Er	PAA; Aptamer	52±11	-	CEA	0.05-10 ng/mL	0.008 ng/mL	92.0%-110.0%	1.4%-6%	261
NaYF_4_:Yb,Tm	PDA	-	-	CEA	0.1-100 ng/mL	0.031 ng/mL	92.6%-107.0%	1.0%-3.0%	262
NaYF_4_:Yb,Tm	TiO_2_	150	-	CEA	0.01-40 ng/mL	3.6 pg/mL	-	8.9%	263
NaErF_4_:Tm@NaYF_4_@NaNdF_4_	PAA; G-quadruplex DNAzyme; cDNA	399.9	-14.16	CEA	50-2000 ng/mL	0.910 pg/mL	94.12%-103.92%	2.12%-5.33%	264
NaYF_4_:Yb,Er	cDNA	30	-35.6	CEA	0.02-6.0 ng/mL (0.1-30 pM)	65 fM (0.013 ng/mL)	-	-	265
NaGdF_4_:Yb,Er	PEG; Thiolated ssDNA;	10±3	-	TK1 mRNA	1.17-65.21 fmol/10 µg	0.67 fmol/10 µg	-	-	266
				microRNA-21	0.043-41.25 fmol/10 µg	0.019 fmol/10 µg	-	-	
NaYF_4_:Yb,Er	PEI; Molecular beacon	-	-	TK1 mRNA	0-100×10^-9^ M	1.1×10^-9^ M	-	-	267
NaGdF_4_:Yb,Er	PEG; Cell-penetrating peptide;	-	-	microRNA-21	0.073-43.65 fmol/10 μg	0.03 fmol/10 μg	-	-	268
NaYF_4_@NaYF_4_:Yb,Er@NaYF_4_ ​	PEG; Anchor DNA; Glycerol phosphate disodium salt hydrate;	-	-	microRNA-21	10×10^-15^- 10×10^-11^ M	0.74×10^-15^ M	95.0%-96.7%	-	269
NaYF_4_:Yb:Tm@NaYF_4_	Plasmid DNA	46.83	-12.0	ctDNA (KRAS)	5-1000 pM	6.30 pM	93.14%-98.51%	2.02%-4.80%	271
NaYF_4_:Tm,Yb@NaYF_4_ ​	SiO_2_; DNA probe	60	-	ctDNA (PIK3CA E542K)	1-500 fM	1 nM	-	-	272

PEG: polyethylene glycol; PAA: polypropylene acid; SBR: signal-to-background ratio; IHC: immunohistochemistry; PEI: polyethyleneimine; PDA: polydopamine; WB: western blotting; NPTAT: nickel (II) phthalocyanine-tetrasulfonic acid tetrasodium salt.

**Table 6:** Applications of UCNPs in BC phototherapy.

UCNPs	Beneficial molecules	Application	Results	Ref.
NaYF_4_:Yb,Tm@NaYF_4_	Riboflavin	PDT	In SK-BR-3 xenograft mice, tumor volume inhibition rate reached 90% post-PDT; Treatment depth achieved 4-6 mm	280
NaYF_4_:Yb,Er,Gd@NaYF_4_	RB	PDT	Under 980 nm light irradiation, SK-BR-3 cell death rate 67%; Dark toxicity 5%	388
NaYF_4_:Yb,Er/Tm@NaYF_4_:Yb@NaYF_4_	Protoporphyrin IX	PDT	Experimental group tumor volume decreased 50% vs. control on day 15	281
NaYF_4_:Yb,Er@NaYF_4_	KillerRed	PDT	Achieved 70% PDT efficiency through 1 cm tissue; KillerRed showed 7% under identical conditions	282
NaYF_4_:Yb,Er	ZnPc	PDT	After 5 min 975 nm irradiation, SK-BR-3 cell viability reduced to 21.8%; MCF-7 cells showed slight decrease under identical conditions	216
NaYF_4_:Yb,Tm	Graphene quantum dots	PDT	20 days post-treatment, 4T1 tumor inhibition rate reached 75.3%	283
NaGdF_4_:Yb,Er@NaGdF_4_:Yb,Nd@NaGdF_4_	Porphyrins	PDT	Post-PDT, 4T1 cell viability reduced to 27.0%; Suppressed tumor growth over 14 days; 808 nm laser mitigated thermal damage risk during PDT	284
NaY (Mn) F_4_:Yb,Er	Chlorin e6	PDT	4T1 relative tumor volume increased to 2-3 times initial at 14 days, significantly lower than control group	285
NaYF_4_:Yb,Er	TTD	PDT	^1^O_2_ yield was 36.4%, higher than mTHPC (31%); Targeted MDA-MB-231 cells with high integrin αvβ3 expression via cRGD peptide; After NIR irradiation, cell viability decreased to 28.3%, with negligible toxicity to MCF-7 and NIH 3T3 cells	286
NaYF_4_:Yb,Er@NaLuF_4_	ZnPc	PDT	NIR irradiation reduced 4T1 viability to 38%; 12-day treatment significantly inhibited tumor growth; Material degraded to <6 nm NPs *in vivo*, excreted renally	152
NaYF_4_:Yb,Er	Gold shell	PTT	UCL/MRI dual-modal imaging enabled; With magnetic field near tumor, NP accumulation increased 8-fold vs. no field; Magnetically targeted NPs combined with 808 nm laser eliminated 4T1 tumor completely	294
NaYF_4_:Yb,Er	AuNPs	PTT	With 5-min NIR irradiation, MCF-7 cell viability reduced >60%. The 525/545 nm green emission ratio allowed ratiometric temperature sensing (25-50 °C) for monitoring	295
NaGdF_4_:Yb:Er	RB; IR825	PTT-PDT	UCL/MRI dual-modal imaging enabled; Combined therapy significantly inhibited tumor growth	297
NaYF_4_@NaYF_4_:Yb,Er@NaYF_4_:Yb,Nd@NaYF_4_ ​	RB; AuNPs	PTT-PDT	Combined therapy reduced the viability of MCF-7 cells to less than 40%	298
NaYF_4_:Yb,Er,Nd@NaYF_4_:Nd	AgBiS_2_	PTT-PDT	160 μg/mL NPs combined with 808 nm laser irradiation (3 min) induced near-zero 4T1 cell survival; 0.5 W/cm^2^ 808 nm laser (10 min) raised tumor temperature to 56.3 °C, sustaining tumor inhibition for 14 days	299

RB: rose bengal; ZnPc: zinc phthalocyanine; TTD: 2-(2,6-bis ((E)-4-(phenyl (40-(1,2,2-triphenylvinyl)-[1,10-biphenyl]-4-yl) amino) styryl)-4H-pyran-4-ylidene) malononitrile; mTHPC: meso-tetrakis (m-hydroxyphenyl) chlorin.

**Table 7:** Clinical uses of anti-BC drugs.

Drugs	Chemical Structure	Ref.
Hydrophobic drugs		
Docetaxel	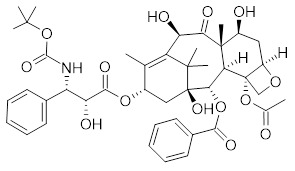	389
Etoposide	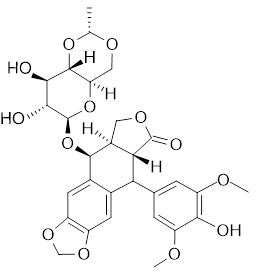	390
Doxorubicin	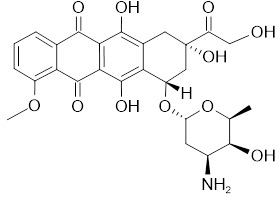	391
Cisplatin	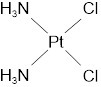	392
Methotrexate	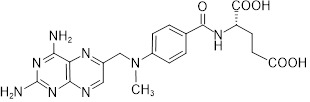	393
Paclitaxel	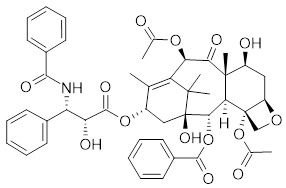	394
Hydrophilic drugs		
Cyclophosphamide	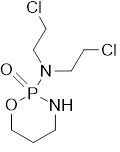	395
Gemcitabine	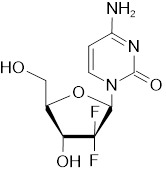	396
5-Fluorouracil	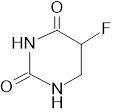	397
Bevacizumab	Monoclonal antibody	398
Pembrolizumab	Monoclonal antibody	399
Trastuzumab deruxtecan	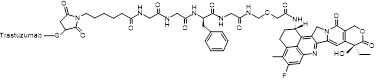	400
Trastuzumab emtansine	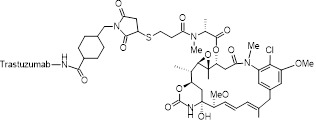	401
Vorinosta	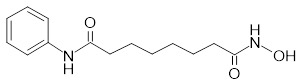	402

**Table 8:** Applications of UCNPs in smart drug/gene delivery.

UCNPs	Agents	Loading method	Loading capacity	Stimulus type	Responder	Results	Ref.
NaYF_4_:Yb,Tm@NaLuF_4_	Chlorambucil	Physical absorption	49%	980 nm light	Amino-coumarin derivative	In dark enzymatic physiological environment, chlorambucil released only 3% within 48 h; With 980 nm light irradiation, over 50% was released within 6 hours, peaking at 68% at 15 h;	308
LiYF_4_:Tm,Yb	DOX	Coordination	Approximately 1500 molecules of doxorubicin were loaded onto each NP	980 nm light	Nitroveratryl	In dark, ultrasound and centrifugation for 60 min induced negligible DOX release; 980 nm light irradiation for 60 min triggered 40% DOX release;	309
NaYF4:Tm,Yb@NaYF_4_	DOX	Physical absorption	4.75%	980 nm light	2-Diazo-1,2-naphthoquinones	In dark, cumulative DOX release remained below 6%; With 980 nm light at 1.0 W/cm^2^ for 60 min, the release reached 30%, and at 1.5 W/cm^2^, it reached 40%;	310
NaYF_4_:Tm,Yb@NaYF_4_	DOX	Physical absorption	-	980 nm light	Spiropyran group	In dark, cumulative DOX release stayed below 5%; 980 nm light induced 80% drug release within 24 h;	311
NaYbF_4_:Tm@NaYbF_4_:Yb,Er	Roussin's black salt	Physical absorption	10%	980 nm light	Roussin's black salt	Burst NO release achieved	312
NaYbF_4_:Tm@NaYF_4_:Yb,Nd@NaYF_4_	Roussin's black salt	Physical absorption	11%	808 nm light	Roussin's black salt	Burst NO release achieved	313
NaYF_4_:Yb,Tm	Plasmid DNA; siRNA;	Physical absorption	0.7%	980 nm light	4,5-dimethoxy-2-nitroacetophenone	Spatiotemporal control of GFP expression achieved	314
NaYF_4_:Yb,Tm	siRNA	Physical absorption	0.7%	980 nm light	Photocaged linker	Spatiotemporal suppression of EGFP expression achieved	315
NaYF_4_:Yb,Tm@NaYF_4_	Bcl-2 antisense oligonucleotides	Physical absorption	Approximately 38 DNA probes containing photocleavable groups were loaded on each NP	980 nm light	Photocleavable linker	Bcl-2 mRNA expression in HeLa cells reduced by 50%	316
NaYF_4_:Yb,Er	DOX	Physical absorption	14.2%	pH	mSiO_2_	DOX release accelerated at pH 5.0, releasing >55% within 96 h; At pH 7.4, release was 35.2%;	403
NaYF_4_:Yb,Tm	DOX	Physical absorption	60%	pH	PEG	At pH 5.8, DOX cumulative release 60% in 10 h; At pH 7.4, release <20%;	380
NaYF_4_:Yb,Tm@NaGdF_4_	Plumbagin	Physical absorption	22.63±1.28%	pH	PAA	At pH 7.4, plumbagin release was 4.32% in 24 h, with negligible release in 96 h; At pH 5.86 and 4.01, 24 h release was 36.37% and 85.72%, stabilizing to 39.79% and 86.17% at 96 h, respectively;	320
NaYF_4_:Yb,Er	DOX	Physical absorption	58.7%	pH	UIO-66(NH_2_)	At pH 7.4, 30% and 40% of DOX were released in 12 and 24 h, respectively; At pH 5.5, 65% and 72% were released over the same times;	222
NaGaF_4_:Yb,Tm@NaGaF_4_: Yb,Er@NaGaF_4_	DOX	Physical absorption	7.9%	pH	Metal-phenolic networks	At pH 5.0, cumulative DOX release reached 51.1%; At pH 7.4, it was only 3.2%;	321
NaYF_4_:Yb,Tm	DOX	Physical absorption	7.23%	980 nm light; pH;	O-nitrobenzyl; Poly(methacrylic acid);	Under visible light at pH 7.4, cumulative DOX release was 8.35% after 5 h; Under 980 nm light irradiation and acidic conditions, it reached 59.5% in the same time;	322
NaYF_4_:Yb,Er@NaYF_4_	DOX; Fe^2+^;	Physical absorption	21.02% (DOX); 4.99% (Fe^2+^);	980 nm light; pH;	Bis-(alkylthio) alkene; Bovine serum albumin;	At pH 7.4, 6.39% DOX released in 48 h; At pH 5.6, 35.76% released in same time; Fe^2+^ release rate significantly accelerated under 980 nm laser irradiation;	323
NaYF_4_:Yb,Er	Camptothecin	Self-assemble	11.5%	980 nm light; GSH;	Eosin Y; Disulfide bond;	NIR light-activated photochemical internalization promoted vesicle escape to cytoplasm, enabling GSH-triggered camptothecin release	326
NaYF_4_:Yb,Er@NaGdF_4_​	DOX	Physical absorption	8.7%	980 nm light; GSH;	CuS; Disulfide bond;	At pH 5.0 with 10 mM GSH, DOX release reached 76% in 48 h under laser irradiation; Without laser, it was 71%;	327
NaGdF_4_:Yb,Er@NaGdF_4_:Nd,Yb	DOX	Physical absorption	54.3%	808 nm light; pH; GSH;	Polyoxometalate; Mn-doped silica;	DOX release rate significantly increased under combined acidic, reductive conditions and NIR light irradiation	328
Y_2_O_3_:Yb,Er@Y_2_O_3_:Yb	DOX	Physical absorption	6.89%	Temperature; pH;	Poly (N-isopropylacrylamide-co-methacrylic acid)	At pH 7.4, DOX release in 36 h was 12.5%; At pH 4.0, it reached 73.6%; At 25 °C, release was 8.1% in 36 h; At 50 °C, it increased to 51.4%;	329
NaYF_4_:Yb,Er	DOX	Physical absorption	-	Temperature; pH;	Poly (N-isopropylacrylamide-co-methacrylic acid)	At low temperature/high pH, DOX showed low release; At higher temperature/lower pH, release increased significantly;	330
NaYF_4_:Yb,Er@NaYF_4_	DOX	Covalent bonding	4.36±0.5%	Enzyme; pH;	Succinic acid-glycine-phenylalanine-leucine-glycine linker	DOX release significantly enhanced under combined enzymatic and acidic conditions	331

siRNA: small interfering RNA; GFP: green fluorescent protein; EGFP: enhanced green fluorescent protein; mRNA: messenger RNA; PEG: polyethylene glycol; PAA: polyacrylic acid.

**Table 9:** Summary of UCNPs combined with immunotherapy.

UCNPs	Immunotherapy agents	Results	Ref.
NaYF_4_:Yb,Er,Gd	Ovalbumin	Highly sensitive *in vivo* UCL imaging for DC migration tracking achieved; UCNP-based DC vaccine induced strong antigen-specific immune responses	335
NaYF_4_:Yb,Tm@NaYF_4_	Zn_x_Mn_1-x_S	Significant inhibition of primary 4T1 tumor and lung metastasis growth	382
NaYF_4_:Yb,Er	MC540; Ovalbumin/Tumor cell fragment	More effective tumor growth inhibition and survival prolongation by nanovaccine vs. PDT/immunotherapy alone	337
NaYF_4_:Yb,Er	DOX; RB	Combination therapy induced 73.1% 4T1 cell apoptosis, significantly higher than single PDT (47.2%) or chemotherapy (24.5%); In TNBC mice, intravenous nanocarriers with NIR irradiation inhibited orthotopic tumor growth, suppressed lung metastasis, and prolonged survival;	338
K_3_ZrF_7_:Yb,Er	-	Biodegradable NPs released ions in cancer cells to induce pyroptosis, enhancing anti-tumor immunity and inhibiting tumor growth and lung metastasis	339
NaGdF_4_:Yb,Er	PDA; Ce6; α-PD-1 antibody	Primary tumor ablation; Antitumor immunity activation; BC metastasis inhibition	340
NaGdF_4_:Yb,Er@NaYF_4_@NaYF_4_:Yb,Tm@NaYbF_4_:Nd@NaYF_4_	RB; α-PD-1 antibody	Significant inhibition of primary tumors; Abscopal effect on non-irradiated distant tumors, suppressing growth	341
GdOF:Yb,Er	AuNPs; Anti-CTLA-4 antibody	Combined therapy showed 90% 4T1 tumor volume reduction vs. control; Efficacy superior to single PDT or immunotherapy	342
NaYF_4_:Yb,Er	DOX; RB; Anti-CD73 antibody	Significant 4T1 tumor growth inhibition (93.4%); Reduction in lung metastasis number	343
NaGdF_4_:Yb,Tm@NaGdF_4_	CpG oligonucleotides	NIR light-induced immune response activation in 4T1 tumors, tumor growth inhibition, and survival prolongation	344
NaYF_4_:Yb,Er@NaYF_4_:Nd	ICG; RB; Anti-CTLA-4 antibody	Destruction of primary 4T1 tumors; Inhibition of untreated distant tumors by the NPs	345
NaYF_4_:Yb,Er	R837; Anti-CTLA-4 antibody	Primary tumor elimination; Distant tumor inhibition; Long-term immune memory protection	346
NaYF_4_:Yb,Er@ NaYF_4_ @NaYF_4_:Yb,Tm@ NaYF_4_	α-PD-1 antibody	Reversal of immunosuppressive TME	347

DC: dendritic cell; RB: rose bengal; ICG: indocyanine green; CTLA-4: cytotoxic T-lymphocyte-associated protein 4; PD-1: programmed cell death-1; TME: tumor microenvironment.

## Abbreviations

BC: breast cancer
RE: rare earth
UCNPs: upconversion nanoparticles
NIR: near-infrared
TNBC: triple-negative breast cancer
ER: estrogen receptor
PR: progesterone receptor
HER2: human epidermal growth factor receptor 2
UCL: upconversion luminescence
SPR: surface plasmon resonance
NPs: nanoparticles
PEG: polyethylene glycol
PAA: polyacrylic acid
PEI: polyethylenimine
FDA: Food and Drug Administration
mSiO_2_: mesoporous silica
EDC: 1-ethyl-3-(3-dimethylaminopropyl)carbodiimide
NHS: N-hydroxysuccinimide
FA: folic acid
TME: tumor microenvironment
ROS: reactive oxygen species
EPR: enhanced permeability and retention
FR: folate receptor
MRI: magnetic resonance imaging
CT: computed tomography
PET: positron emission tomography
CA: contrast agent
IHC: immunohistochemistry
WB: western blotting
ELISA: enzyme-linked immunosorbent assay
FRET: Förster resonance energy transfer
VEGF: vascular endothelial growth factor
LOD: limit of detection
CA15-3: cancer antigen 15-3
CA125: cancer antigen 125
CEA: carcinoembryonic antigen
PDA: polydopamine
AuNPs: gold nanoparticles
mRNA: messenger RNA
ctDNA: circulating tumor DNA
PDT: photodynamic therapy
PS: photosensitizers
PTT: photothermal therapy
PTA: photothermal agents
^1^O_2_: singlet oxygen
RB: rose bengal
DOX: doxorubicin
siRNA: short interfering RNA
GSH: glutathione
DC: dendritic cell
ICD: immunogenic cell death
CTLA-4: cytotoxic T-lymphocyte-associated protein 4
GMP: good manufacturing practice
SARs: structure-activity relationships
